# Direct lipid interactions control SARS-CoV-2 M protein conformational dynamics and virus assembly

**DOI:** 10.1038/s41467-026-75229-9

**Published:** 2026-07-23

**Authors:** Mandira Dutta, Kimberly A. Dolan, Souad Amiar, Elijah J. Bass, Rokaia Sultana, Sean M. Braet, Karen J. Cárdenas-Martínez, Devika Sirohi, Ian K. Hicklin, Richard J. Kuhn, Ganesh S. Anand, Gregory A. Voth, Stephen G. Brohawn, Robert V. Stahelin

**Affiliations:** 1 Department of Chemistry, The University of Chicago, Chicago, IL USA; 2 Department of Molecular & Cell Biology, Department of Neuroscience, California Institute for Quantitative Biology (QB3), Biophysics Graduate Program, University of California Berkeley, Berkeley, CA USA; 3 Borch Department of Medicinal Chemistry and Molecular Pharmacology, Purdue University, West Lafayette, IN USA; 4 Purdue Institute of Inflammation, Immunology and Infectious Disease, Purdue University, West Lafayette, IN USA; 5 Department of Chemistry, Pennsylvania State University, University Park, PA USA; 6 Department of Biological Sciences, Purdue University, West Lafayette, IN USA; 7 Chicago Center for Theoretical Chemistry, Institute for Biophysical Dynamics, and James Franck Institute, The University of Chicago, Chicago, IL USA

**Keywords:** Viral infection, Cryoelectron microscopy, Computational models

## Abstract

M is the most abundant structural membrane protein in coronaviruses and is essential for the formation of infectious virus particles. SARS-CoV-2 M adopts two conformations, M_short_ and M_long_, and regulated transition between states is hypothesized to coordinate viral assembly and budding. However, the factors that regulate M conformation and roles for each state are unknown. Here, we discover a direct M-sphingolipid interaction that controls M conformational dynamics, interaction with other structural proteins, and virus assembly. We show M binds Golgi-enriched anionic lipids including ceramide-1-phosphate (C1P). Molecular dynamics simulations show C1P interaction promotes a long to short transition and energetically stabilizes M_short_. Cryo-EM structures show C1P specifically binds M_short_ at a conserved site bridging transmembrane and cytoplasmic regions. Disrupting M_short_-C1P interaction alters M subcellular localization, reduces colocalization with Spike and E, and reduces virus-like particle formation and cell entry. Together, these results show endogenous signaling lipids regulate M structure and support a model in which M_short_ is stabilized in the early endomembrane system to organize other structural proteins prior to viral budding.

## Abstract (web summary)

SARS-CoV-2 M protein binds the host lipid ceramide-1-phosphate, stabilizing its short conformation to organize viral structural proteins during assembly. Disrupting this interaction impairs virus-like particle formation and cell entry.

## Introduction

Coronaviruses are lipid envelope-encapsulated single-stranded RNA viruses with a genome encoding four structural proteins – spike (S), membrane (M), envelope (E), and nucleocapsid (N). The lipids that comprise the envelope are derived directly from host membranes at the site where viral budding occurs^1^. Viruses often utilize specific lipids to drive every step of the infection cycle – from viral entry^2–5^, to biosynthesis^6,7^, assembly and egress^8,9^ – and have been shown to modulate the composition of cellular membranes to facilitate these processes^10^. Lipids are known to play multiple roles in betacoronavirus infection. Mouse hepatitis virus and SARS-CoV-2 target cholesterol- and ceramide-rich microdomains for viral entry^5^ and depletion of cholesterol reduces S receptor binding in cells^11^. Coronavirus genome amplification and biosynthesis are marked by the formation of double-membrane vesicles enriched in triacylglycerols and cholesterol^6,12^. Coronavirus N proteins, whether free or bound to viral RNA, associate with lipid membranes, and specific interactions with anionic lipids may drive localization to sites of viral transcription and assembly^13–15^.

SARS-CoV-2 M is a highly conserved structural membrane protein that facilitates virus or virus-like particle assembly through direct protein-protein interactions with the other structural proteins S, E, and N^16,17^. Coronavirus M proteins adopt distinct conformations, M_short_ and M_long_, in viral envelopes, and cryo-EM structures of SARS-CoV-2 M have been captured in each state in complex with state-specific antibody fragments (Fabs)^16,18,19^. The transition between conformations has been speculated to be important for viral assembly or budding^16,19^. It is hypothesized that interactions between the M protein and specific lipids are critical for these conformational transitions. Recent AFM studies revealed a thinning of the membrane near the M protein dimer^20^. Despite the M protein’s critical roles in infectious viral particle assembly and release, and the growing evidence of the critical importance of lipids in these processes, potential roles for specific M-lipid interactions remain unstudied.

Here, we discover that M directly interacts with sphingolipids, including ceramide-1-phosphate (C1P), in a conformationally selective manner and show that the M-C1P interaction is critical for proper localization, structural protein interaction, and viral assembly.

## Results

### M binds anionic sphingolipids

**Fig. 1:**
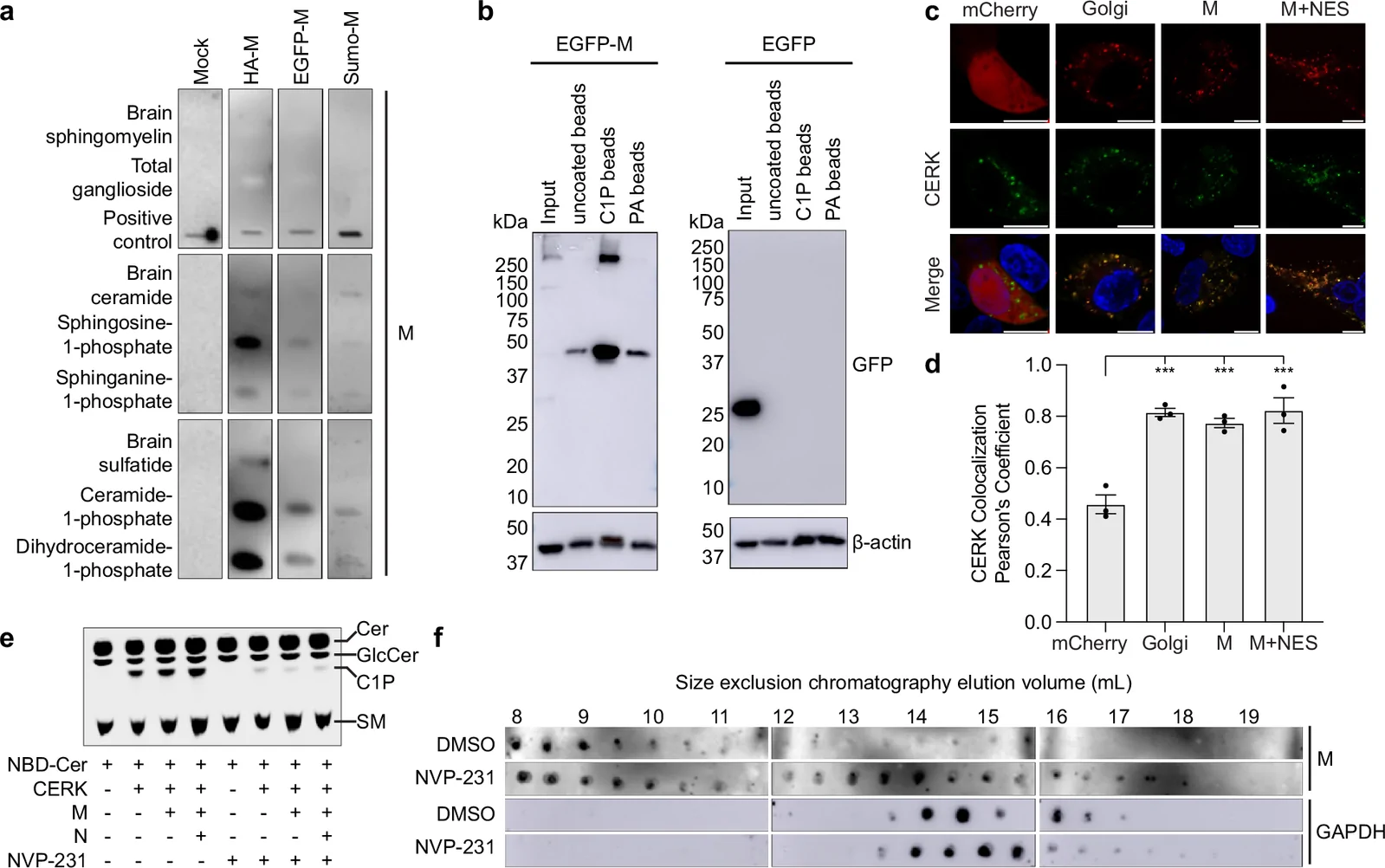
SARS-CoV-2 M protein interacts with C1P. **a** Sphingolipid snooper assay for M-lipid interactions. Western blot of immobilized lipids probed with N-terminal HA-tagged M, a 50:50 mixture of N-terminal EGFP-tagged and untagged M, N-terminal His-SUMO-TEV-M, or a control sample without M protein. All M constructs tested displayed strong binding to C1P and dihydro-C1P, as well as appreciable binding to anionic So1P and Sa1P. Two biological replicates were performed. **b** Bead pull-down assay of M-lipid interactions. Western blot of input EGFP-M (left) or EGFP (right) expressing cell lysate or eluant from control uncoated, C1P-coated, and PA-coated beads. EGFP-M protein was robustly pulled down by C1P-coated beads. Two biological replicates were performed. **c**, **d** Colocalization of SARS-CoV-2 M with CERK. **c** Representative images of HEK293 cells expressing GFP-CERK and mCherry, Golgi7-mCherry, mCherry-M, or mCherry-M and N, E, and S. Scale bar = 10 µm. **d** Quantification of CERK colocalization (mean ± SEM, *n* = 3 biological replicates with 6 cells per replicate, differences assessed with a one-way ANOVA with Tukey correction for multiple comparisons (***p = 0.0003, 0.0007, and 0.0002, respectively)). Source data are provided as a Source Data file. **e** Assay for C1P production. Thin-layer chromatography of samples from mock-, CERK-, CERK and M-, or CERK, M, and N-transfected HEK293 cells treated with 2.5 µM NBD-ceramide and either DMSO or 100 nM CERK inhibitor NVP-231. CERK increased C1P levels and was inhibited by NVP-231. (SM sphingomyelin, Cer ceramide, GlcCer glucosylceramide). **f** Assay for M oligomerization in cells. Western blot for M (upper) or GAPDH (lower) in size exclusion chromatography fractionated CERK, M, N, E, and S-expressing HEK293 cell lysate treated with DMSO or 100 nM NVP-231. Later-eluting fractions correspond to smaller species. Lower C1P levels altered the M protein oligomerization state, with an increased proportion of smaller species detected.

Virus assembly and budding often require lipid–protein interactions for assembly site formation and budding and egress of virus particles. To determine if M protein binds specific lipids, we screened purified M protein for binding to an array of sphingolipids. HA-tagged M protein robustly interacted with C1P and dihydro-C1P, with weaker binding to sphingosine-1-phosphate (So1P) and sphinganine-1-phosphate (S1P) (Fig. 1a). To control for construct-specific effects, we also tested His-SUMO-tagged M protein and a 50:50 mixture of EGFP-tagged and untagged M. Under all these conditions, M displayed the most robust binding to C1P (Fig. 1a). M did not bind to a host of other lipids tested (Supplementary Fig. 1a, b). To confirm M-C1P binding in a complementary assay, we compared M binding to C1P-coated beads, anionic phosphatidic acid (PA)-coated beads, and control uncoated beads. M protein displayed increased binding to C1P-coated beads over control and PA-coated beads (Fig. 1b).

Ceramide kinase (CERK) is the major producer of C1P in mammalian cells^21^. To assess the role of CERK in M assembly, we imaged M and other structural proteins under different conditions. M protein colocalized with the S and E proteins (Supplementary Fig. 2). M protein also colocalized with markers of the endoplasmic reticulum-Golgi intermediate compartment (ERGIC) and Golgi (Supplementary Fig. 3a–c), in contrast with an ER marker (Supplementary Fig. 3c) and formed assembly sites similar in vesicle diameter (Supplementary Fig. 3d–f) to authentic SARS-CoV-2^22^. A GFP-CERK construct displayed colocalization with SARS-CoV-2 assembly sites labeled with M protein (Fig. 1c). To assess CERK and M protein cellular localization, we expressed CERK, CERK and M, or CERK, M, N, E and S in HEK293 cells and imaged CERK, M, and a Golgi marker (Fig. 1c, d). CERK significantly colocalized with the Golgi marker and M under both conditions compared to mCherry alone.

We next probed CERK activity by assessing C1P content from cells using thin-layer chromatography. C1P production was increased upon CERK expression (Fig. 1e). Next, we treated cells with the CERK inhibitor NVP-231 to confirm a reduction in C1P levels, which was similarly lowered under all conditions with CERK expression (Fig. 1e). We then expressed M, S, E, and N with CERK in cells and fractionated lysates using size exclusion chromatography (Fig. 1f). Inhibition of CERK resulted in an increase in later-eluting, smaller M oligomers, demonstrating a reduction in higher-order oligomerization of M protein when C1P was depleted.

### C1P binds and stabilizes M_short_

**Fig. 2:**
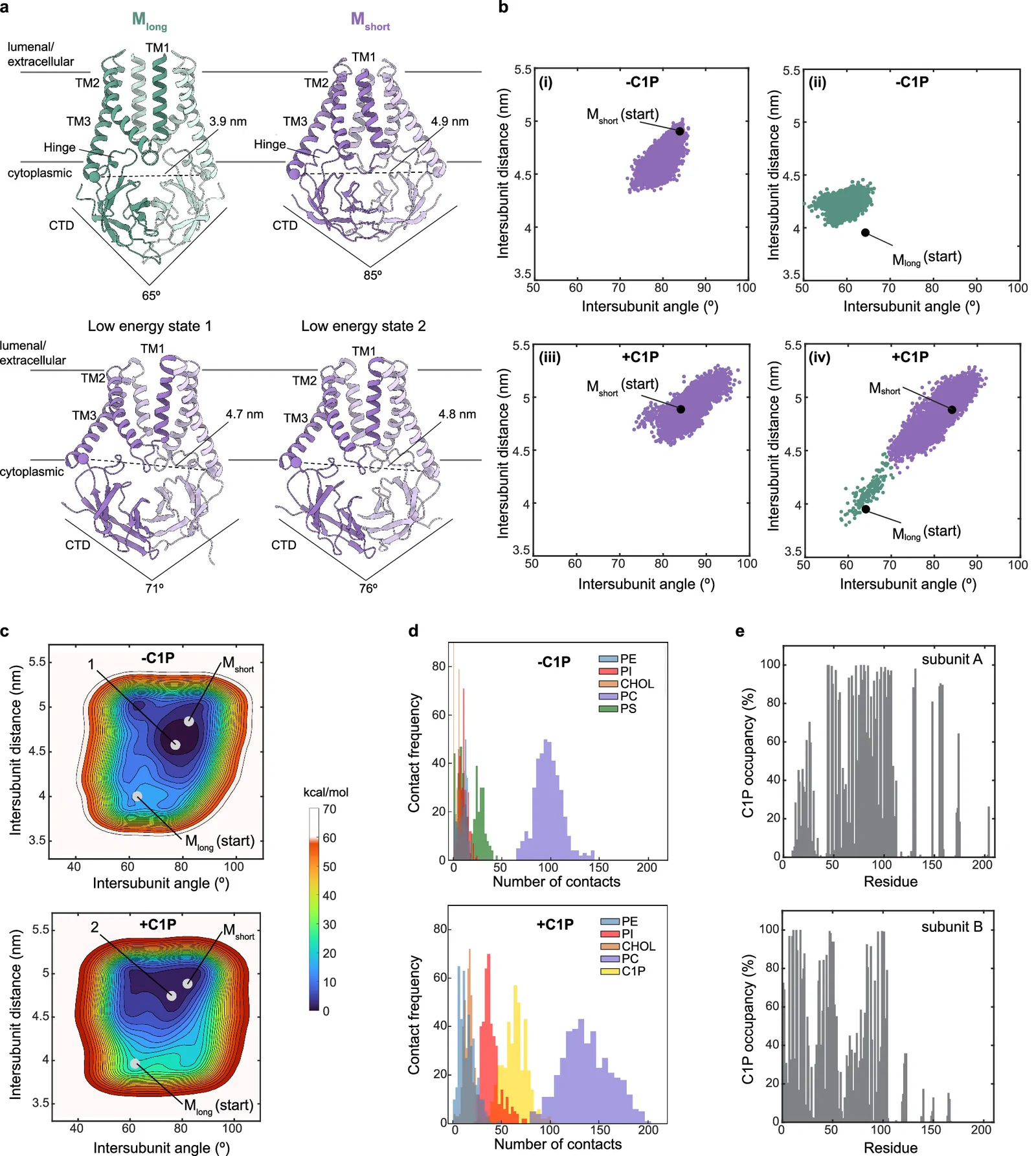
C1P energetically stabilizes M_short_. **a** Structures of M protein viewed from the membrane plane. Collective variables that report on intersubunit distance and angle used to monitor conformational state during simulations are illustrated on the structures. **b** M protein conformational trajectory during molecular dynamics simulations. Intersubunit angle versus intersubunit distance of the structure at each time sampled during 4 µs simulations is plotted. Simulations were performed without (i, ii) or with C1P (iii, iv) and initiated from M_short_ (i, iii) or M_long_ (ii, iv). Conformational switching was only observed from M_long_ to M_short_ in the presence of C1P. **c** Free-energy landscape calculated from simulations without (above) or with C1P (below). Position of M_short_, M_long_, and a low-energy state from each condition is indicated. **d** M-lipid contact frequency from simulations without (above) and with C1P (below). **e** M-C1P percentage occupancy by residue in subunit A (above) and subunit B (below). Source data are provided as a Source Data file.

We used molecular dynamics (MD) simulations to better understand the nature and consequences of M-C1P interactions. Atomistic models of M were constructed from M_short_ (7VGS) and M_long_ (7VGR) structures embedded in lipid membranes^19^. Membrane composition mimicked the composition of the ERGIC (45% PC, 20% PI, 15% PE, 10% Cholesterol) with either 10% C1P or 10% PS as a control anionic lipid^23,24^. The conformation of M was monitored during the 4 µs simulations by two collective variables that distinguish M_short_ from M_long_: (1) the angle between cytoplasmic C-terminal domains (CTDs) of each subunit and (2) the distance between hinge regions (Fig. 2a).

Strikingly, MD simulations initiated from M_long_ in the presence of C1P showed rapid conformational switching to M_short_-like conformations, which remained stable for the duration of the simulation (Fig. 2b). Conformational switching was dependent on C1P, as simulations initiated from M_long_ in the absence of C1P did not exhibit a transition to the short state. During the short equilibration prior to the production run in these simulations, the M_long_ structure slightly deviated from the initial model to sample a state resembling, but not identical to, the M_long_ conformation captured by cryo-EM (Fig. 2b, Supplementary Fig. 4j). This difference may be due to the Fab present in cryo-EM experiments, but absent in MD simulations, stabilizing the captured M_long_ conformation in the absence of C1P. Notably, conformational switching in the MD simulations was only observed from M_long_ to M_short_; M_short_ in the presence or absence of C1P remained in M_short_-like conformations throughout the simulations (Fig. 2b, Supplemental Movies 1–4). This is consistent with the stability of M_short_ we observed in prior simulations in the absence of C1P^18^.

These results suggest that C1P stabilizes M_short_. We computed the conformational free-energy landscape using well-tempered (WT) metadynamics^25^, employing the same collective variables of the intersubunit CTD angle and hinge distance as the pertinent coordinates (Fig. 2c). We reach two conclusions from this analysis. First, M_short_ is a lower free-energy state than M_long_, regardless of lipid composition. Minimum energy states in the absence (indicated by “1”) and presence of C1P (indicated by “2”) are distinct, but similarly M_short_-like (Fig. 2a). Second, C1P markedly stabilizes M_short_. We calculate the magnitude of stabilization to be –6.99 ± 1.00 kcal/mol (defined as $\Delta \Delta G=\Delta {G}_{+C1P}-\Delta {G}_{-C1P}$= (−6.99 ± 1.00 kcal/mol = ((−18.07 ± 0.62) – (−11.08 ± 0.79)) kcal/mol)). Stabilization is primarily due to a deeper free energy well around M_short_-like conformations.

C1P stabilization could be explained by direct binding to M_short_. We observed more interactions between residues in the CTD and lipid headgroups in MD simulations with C1P, with enrichment of C1P and PI contacts (Fig. 2d). C1P and PI lipids were localized around M_short_ in visualizations of lipid position (Supplementary Fig. 4a). Analysis of membrane structure during the last 2 μs of the simulations with C1P shows M subtly thins and curves the membrane, particularly around the CTD-inner leaflet interface (Supplementary Fig. 4b–e). To identify candidate residues involved in M-C1P interactions, we calculated residence time (Supplementary Fig. 4e) and percentage occupancy (Fig. 2e) of C1P around M by amino acid. A continuous patch of residues at the TM-CTD interface showed both high C1P occupancy (>80%) and among the longest C1P residence times. The patch includes residues at the bottom of TM1 (R44, F45, and I48), bottom of TM3, hinge (A104, R107, S108, M109, and W110), proximal CTD (L129 and R131), and nearby charged residues in the CTD (H148, H155, and R158).

To investigate mechanisms underlying C1P-mediated conformational transition from the M_long_ to M_short_, we first calculated the number of contacts between positively charged residues of the CTD and the negatively charged phosphate group of C1P over time (Supplementary Fig. 4f, g). In simulations initiated from M_long_ with C1P, the number of observed C1P interactions increases as the M conformation changes. The number of C1P contacts is correlated with conformational state: up to intersubunit angles of 70° and intersubunit distances of 4.3 nm (M_long_-like conformations), C1P recruitment is low (Supplementary Fig. 4f). Beyond these values (in M_short_-like conformations), a higher number of C1P molecules are recruited. In contrast, simulations initiated from the M_short_ structure in the presence of C1P show consistently high contacts throughout the simulation (Supplementary Fig. 4g). Our metadynamics simulations further confirm the enrichment of C1P lipid head groups in contact with M_short_ CTD (Supplementary Fig. 4g, h). Gradual transition from M_long_ to M_short_ driven by C1P recruitment is evident in trajectory movies of the simulations (Supplementary Movies 1–4). Together, these analyses suggest an induced-fit mechanism for C1P binding and promotion of the conformational transition from M_long_ to M_short_.

**Fig. 3:**
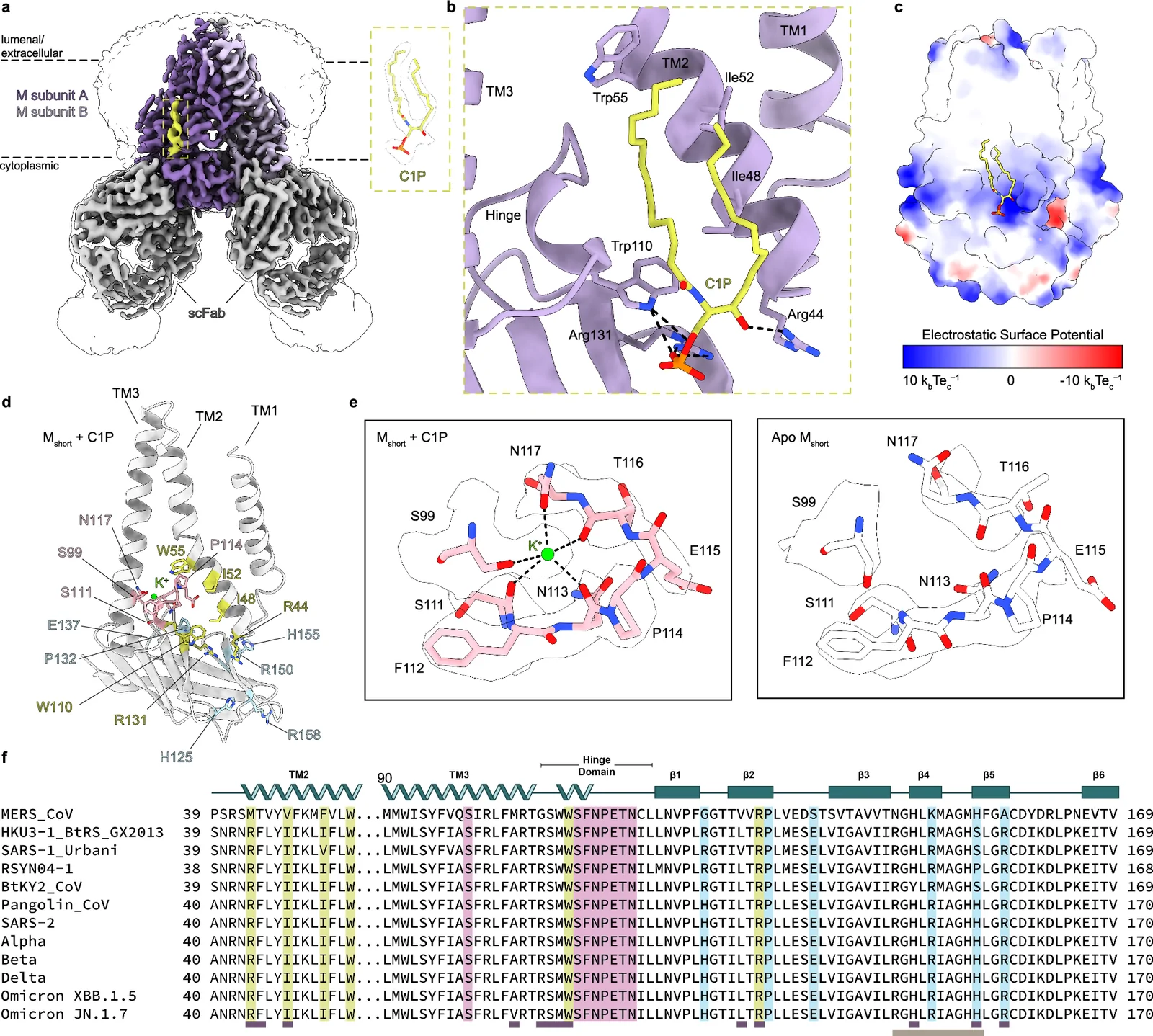
Cryo-EM structure of M_short_ bound to C1P. **a** 3.0 Å resolution cryo-EM map of SARS-CoV- 2 M in the short conformation viewed from the membrane. M subunits are purple, short conformation-selective Fabs (scFab) are gray, and C1P is yellow. An unsharpened map is shown transparent at low contour. **b** Zoomed in view of the C1P-binding site in one subunit. **c** Electrostatic surface view of M with C1P bound. **d** Single M_short_-C1P subunit with C1P-binding residues yellow, K^+^ binding residues pink, and residues displaying associated conformational changes blue. **e** Zoomed in view of the K^+^ binding site in M_short_-C1P (left) or apo M_short_ (right). Cryo-EM density is shown as a transparent surface. **f** Sequence alignment of M across coronaviruses with residues colored as in (**d**). Below the alignment, positions implicated in C1P interaction by MD are marked with dark bars, and the residue range mutated in M^ΔC1P^ is indicated by a light bar.

We next sought direct experimental structural insight into the molecular basis of M-C1P interaction. We prepared complexes of M with CTD-binding Fabs that stabilize either M_short_ or M_long_ conformations in the presence of 100 µM C1P (short conformation Fab (scFab) and long conformation Fab (lcFab)). To facilitate comparisons to previously reported apo M structures, buffer and detergent conditions were otherwise identical. We determined cryo-EM structures of M_short_ and M_long_ in the presence of C1P to 3.0 Å overall resolution (Fig. 3a, Supplementary Figs. 5 and 6, Supplementary Table 1).

The M_short_-C1P map shows lipid-like densities not visible in apo M_short_ maps. One density feature per subunit at the inner leaflet can be unambiguously modeled as C1P with a well-defined phosphomonoester headgroup and acyl chains (Fig. 3a). C1P binds to a site formed by the bottom of TM1-3, hinge region, and membrane-proximal aspect of the CTD (Fig. 3b). The C1P head group fits into an electropositive pocket and forms polar interactions with R44, W110, and R131 (Fig. 3c). C1P acyl chains pack into a cleft between TM2 and TM3, forming hydrophobic interactions with F45, I48, L51, I52, W55, M109, W110, and P114 on TM2 (Fig. 3b, d). Density corresponding to the inner acyl chain is weakly visible in the apo M_short_ map, suggesting partial occupancy by co-purified lipids or detergents in the absence of C1P. Notably, a similar binding mode for C1P at this site was consistently observed in our MD simulations (Supplementary Fig. 4i).

In contrast, we see no evidence for specific C1P binding to M_long_ (Supplementary Fig. 7a). M_long_ + C1P and apo-M_long_ structures are nearly indistinguishable (0.79 Å overall r.m.s.d.). Densities consistent with detergent or lipid acyl chains around the inner leaflet are similar in M_long_ + C1P and apo-M_long_ maps. The absence of C1P binding to M_long_ is explained by the reorganization of the M_short_-C1P-binding site. In M_long_, the two halves of the C1P-binding pocket (TM1/2 and TM3/hinge/CTD) spread apart, separating key residues R44 and R131 by >7 Å (Supplementary Fig. 7b).

M_short_-C1P is captured in a conformation that is similar, but not identical, to apo M_short_ structures captured in lipid nanodiscs or in complex with M_short_-specific Fabs (Supplementary Fig. 8a). Comparing the structures reveals four differences with potential functional consequences. First, C1P-binding compacts M, particularly in the TM region, where intersubunit TM2-TM3 distances are 1.5–3.5 Å shorter. Second, C1P binding increases overall rigidity of M. M_short_-C1P has more clearly resolved flexible regions (including in the hinge and TM linkers) and lower, less variable B-factors than apo M_short_, despite similar particle numbers, processing routines, and overall resolutions (Supplementary Fig. 8c). Third, C1P binding reveals an ion binding site bridging the hinge and bottom of TM3 (Fig. 3d, e). Electronegative groups, including S99 and N117 side chain hydroxyls and S111, N113, and T116 backbone carbonyls, surround a strong density feature not visible in the apo M_short_ map. We model a K^+^ in this site supported by buffer conditions (150 mM KCl), coordination geometry, and multiple validation tools. Fourth, a series of conformational changes propagates from the C1P-binding site and alters the CTD surface character. Hinge residue W110 is shifted towards the CTD upon C1P binding, necessitating a shift in neighboring P132 on the β2 strand (Supplementary Fig. 8b). Charged residues including H125 on β1, R158 on β5, and E137, R150 and H155 at the intersubunit CTD interface change rotameric state, altering the electrostatic character of the CTD surface and potentially influencing M interactions with other structural proteins during virus assembly (Supplementary Fig. 8d). Residues in M implicated in C1P binding are invariant across betacoronaviruses, including SARS-CoV-2 clinical isolates, suggesting the interaction is highly conserved (Fig. 3f). We conclude C1P specifically binds and stabilizes a distinct M_short_ conformation.

Notably, residues involved in C1P-binding and associated conformational changes have been recently implicated in the action of two anti-coronavirus inhibitors that prevent virus assembly and budding^26,27^. Both small molecules (CIM-834 and JNJ-9676) bind M_short_ (but not M_long_) in a pocket above the hinge between TM2 and TM3. This position overlaps the potassium ion binding site identified here and is proximal to the C1P-binding site (Supplementary Fig. 9a). The inhibitors are proposed to trap an M_short_-like conformation and prevent transition to M_long_, suggesting this conformational transition is critical to the viral life cycle. Two escape mutants were identified in M that drastically reduce sensitivity to the inhibitors (P132S, S99A). We reasoned these mutations could disrupt drug binding in part by promoting M_long_ and that other residues implicated in C1P binding would have similar effects. We used AlphaFold3 to test this hypothesis. Wild-type M was predicted to adopt an M_short_-like conformation (Supplementary Fig. 9b), consistent with our structural and MD results. In contrast, mutants at sites implicated in C1P binding or associated conformational changes (H155E, E137R, R150D, W110R, P132W, and S99D) are predicted to adopt increasingly M_long_-like conformations (Supplementary Fig. 9b). Among these structures, M_short_-C1P is the most compacted in a continuum from short to long conformations.

### M interconverts between conformational states, is predominantly M_short_-like in solution, and M_long_-like in VLPs

The nature and physiological relevance of SARS-CoV-2 M_long_ is uncertain because M_long_ and M_short_ were visualized in virions and VLPs prior to the emergence of SARS-CoV-2, and capturing M_long_ cryo-EM structures required the presence of stabilizing Fabs. To better understand the nature of M_short_-M_long_ transitions, we applied comparative amide hydrogen–deuterium exchange mass spectrometry (HDXMS) to directly observe the M conformational distribution in different conditions and over time. HDXMS is well suited to probing the conformational dynamics of viral protein complexes^28–30^, as slowly (s-min) interconverting conformations can be detected through bimodal mass spectral envelopes corresponding to EX1 hydrogen–deuterium exchange kinetics^31,32^. We analyzed M under different conditions and observed good coverage in hinge and CTD regions in all cases with 98 peptides for apo M in detergent, 49 peptides for M bound to C1P or Fabs in detergent, and 18 peptides for M embedded in VLPs, covering 69%, 63%, and 69% of the total M sequence, respectively (Supplementary Fig. 10a).

**Fig. 4:**
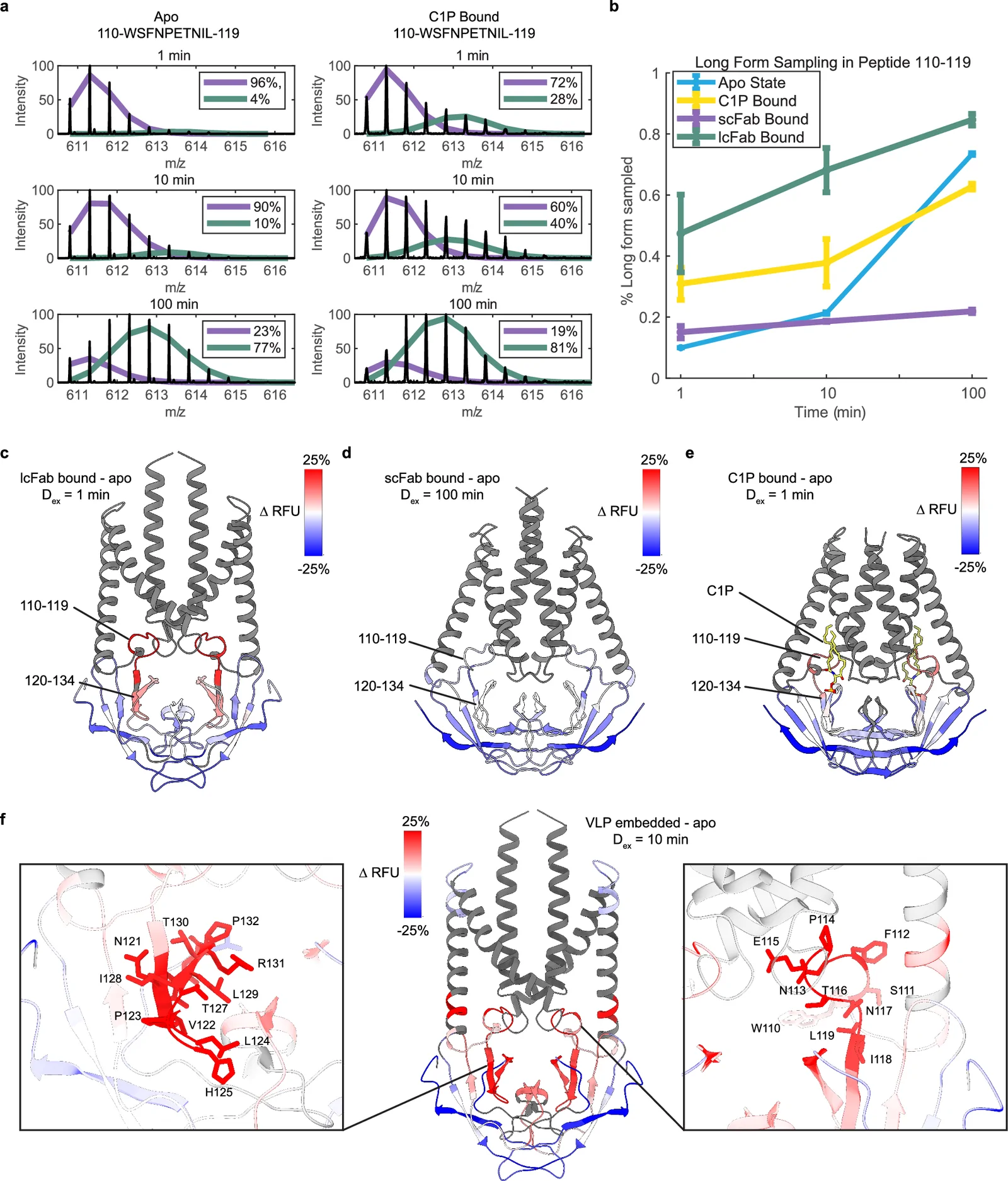
M_long_ is sampled by M in solution and is the dominant M conformation in VLPs. **a** Stacked mass spectral envelopes for hinge reporter peptide 110–119 corresponding from (left) apo M and (right) C1P-bound M, at 1 min, 10 min, and 100 min exchange (top to bottom). Bimodal spectra were deconvolved, and high- and low-exchanging population fits are shown in green and purple, respectively. **b** Plot of the percentage of the M_long_ conformation sampled over time in apo (cyan), C1P-bound (yellow), scFab-bound (purple), and lcFab-bound (green) samples. The percentage of M_long_ sampled was defined as the percentage of the high-exchanging population present in bimodal mass spectral envelopes. Mean ± sem across 3 technical replicates is plotted. Source data are provided as a Source Data file. **c** Deuterium exchange differences for lcFab-bound minus apo M after 1 min exchange mapped onto the lcFab-bound M_long_ structure (7VGR), **d** scFab-bound minus apo M after 100 min exchange mapped onto the scFab-bound M_short_ structure (7VGS), and **e** for C1P-bound minus apo M after 1 min exchange mapped onto the C1P-bound structure with C1P represented in yellow. Differences in deuterium exchange are mapped as a blue-white-red gradient from −25% to 25% relative fractional deuterium uptake. **f** Deuterium exchange differences for VLP-embedded minus apo M after 10 min exchange mapped onto the lcFab-bound M_long_ structure (7VGR). Differences in deuterium exchange are mapped as a blue-white-red gradient from −25% to 25% relative fractional deuterium uptake. Residues 110–119 and 120–134 are highlighted in insets.

We first probed differences between M_long_ and M_short_ by comparing hydrogen–deuterium exchange profiles of apo M with M in complex with either scFab or lcFab (short conformation Fab (scFab) and long conformation Fab (lcFab)) over time. Consistent with complex formation, M-lcFab and M-scFab samples showed stable decreased exchange relative to apo M in residues comprising the Fab-binding epitopes in the CTD (Supplementary Fig. 10b). Notably, we observed bimodal spectral envelopes for deuterium-exchanged reporter peptides spanning the hinge (110–119) and adjacent CTD β1 and β2 strands (120–134) with marked differences between samples (Fig. 4a–e, Supplementary Figs. 10b and 11a–e). M-scFab showed low exchange, while M-lcFab showed high exchange in these peptides at all time points (Fig. 4a–d). In contrast, apo M showed low exchange at short time points, similar to M-scFab, and accumulation of a high-exchanging population over time, similar to M-lcFab (Fig. 4a–d, Supplementary Fig. 11a–e). We conclude apo M is predominantly M_short_-like in solution, but samples M_long_-like conformations on the minute timescale.

We next tested C1P-bound M samples. We observed decreased deuterium exchange throughout the CTD in response to C1P binding, reflective of both the altered electrostatics in the intravirion beta sheets and a more compact overall M structure (Fig. 4a, b, e, Supplementary Fig. 10b, Supplementary Fig. 11a–e). Deuterium uptake in CTD β1 and β2 (120–134) was similar in apo- and C1P-bound M samples, consistent with C1P binding to M_short_-like conformations. Bimodal mass spectral envelopes were similarly observed in the hinge peptide (110–119) of C1P-bound M. The percentage of the high-exchanging population in C1P-bound M is higher than apo- or scFab-bound M, but lower than lcFab-bound M at 1 and 10 min timepoints. This indicates that while M_short_-like conformations remain the dominant population, C1P binding results in distinct conformations with increased accessibility of the hinge locus. This is consistent with results from MD and cryo-EM (Fig. 2b, Supplementary Fig. 8).

We then applied comparative HDXMS to apo M and M embedded in VLPs. We observed a distinct decrease in deuterium exchange within the M CTD (residues 170–205) (Fig. 4f, g, Supplementary Fig. 10c), which may be due to interactions between M and N or other components in VLPs. In contrast, residues spanning the hinge (110–119) and CTD β1 and β2 (120–134) showed large increases in deuterium exchange, similar to that observed in M_long_ conformations promoted by lcFab binding (Fig. 4f, g, Supplementary Figs. 10c and 11f, g). We conclude M_long_-like conformations are dominant within assembled virus-like particles.

**Fig. 5:**
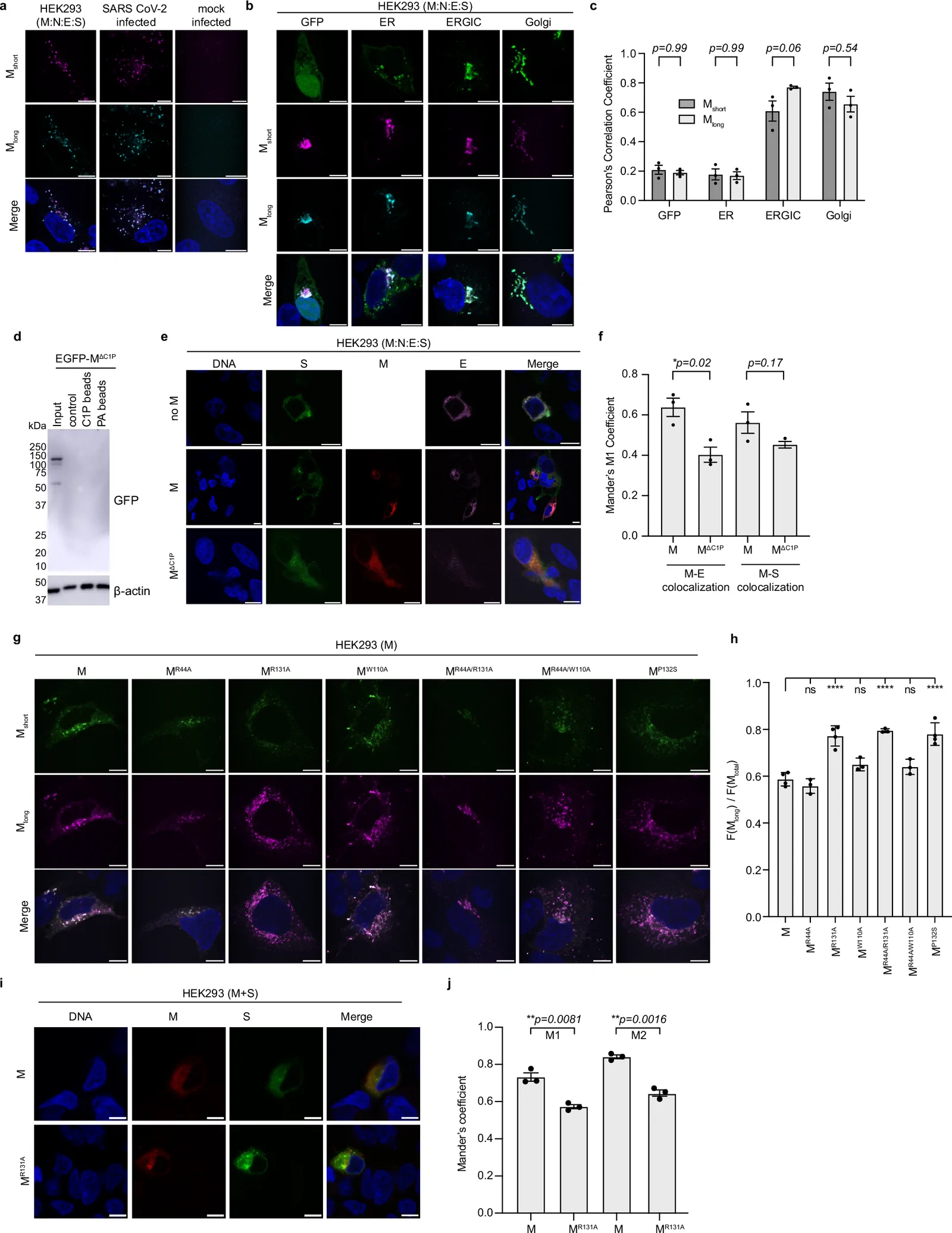
Disrupted M-C1P interaction alters M_short_-M_long_ conformational distribution and colocalization with structural proteins. **a** M_short_ and M_long_ immunolocalization in representative transfected (Three biological replicates with four technical replicates each), SARS-CoV-2-infected (*N* = three infections of SARS-CoV-2 under different post-infection time points), and mock-infected cells (*N* = three mock infections under different post-infection time points). M_short_ and M_long_ are observed in overlapping and distinct pools. The left panel and the middle panel are duplicated in Fig. S12a, d, respectively, for comparison across panels. Scale bar = 10 µm. **b** Colocalization of M_short_ and M_long_ with markers for ER (ER3), ERGIC (ERGIC53), or Golgi (Golgi7) in cells transfected with M, N, E, and S. Scale bar = 10 µm. **c** Quantification of colocalization experiments in (**b**) (mean ± SEM, *n* = 3 biological replicates with 6 cells per replicate). **d** Bead pull-down assay of M^ΔC1P^ mutant-lipid interactions. Western blot of input EGFP-M^ΔC1P^ expressing cell lysate and eluant from uncoated, C1P-coated, or PA-coated beads. Two biological replicates were performed. **e** Colocalization of M and M^ΔC1P^ with S and E in cells transfected with M, N, E, and S. Scale bar = 10 µm. **f** Quantification of colocalization experiments in (**e**) with fraction of M (M1) that overlaps with E and S, respectively, indicated (mean ± SEM, *n* = 3 biological replicates with at least 3 cells per replicate). **g** M_short_ and M_long_ immunolocalization in representative HEK293 cells transfected with the indicated M constructs. **h** Quantification of M_long_ proportion (M_long_ fluorescence units/(total M (M_long_+M_short_) fluorescence units) from experiments in (**g**) (mean ± SEM, *n* = 3 or 4 biological replicates with 6 cells per replicate). **i** HEK293 cells transfected with GFP-S and M (top) or M^R131A^ (bottom) and immunostained for M. Scale bar = 10 µm. **j** Quantification of colocalization experiments in (**i**) with fraction of S that overlaps with M (M1) and fraction of M that overlaps with S (M2) indicated (mean ± SEM, *n* = 3 biological replicates with at least 3 cells per replicate). Data are mean ± SEM with differences assessed with **c**, **h** one-way ANOVA with **c** Sidak or **h** Dunnett corrections for multiple comparisons or **f**, **j** unpaired two-tailed *t*-test with Welch’s correction (****p < 0.0001). Source data are provided as a Source Data file.

To assess whether M is present in long and short conformations in cells, we immunolocalized M_short_ and M_long_ with conformation-specific antibodies or fluorophore-labeled scFab and lcFab. A549-hACE2 cells were infected with SARS-CoV-2 (SARS-CoV-2/human/USA/WA-CDC-WA1/2020) and compared to mock-infected cells and tested for the presence of M_short_ and M_long_ (Fig. 5a and Supplementary Fig. 12d, e). Infected cells displayed detection of both M_long_ and M_short_ (Fig. 5a and Supplementary Fig. 12d), in contrast to no signal in mock-infected cells (Supplementary Fig. 12e). M_long_ and M_short_ displayed a high level of overlap with respect to cellular localization, but were also present in distinct pools evident by a line profile analysis (Supplementary Fig. 12f–h). M_short_ and M_long_ were also both detected in cultured cells transfected with M alone or with M, N, E, and S (Fig. 5a), predominantly at the Golgi and ERGIC (Fig. 5b, c and Supplementary Fig. 12a–c) in contrast to the ER or colocalized with soluble GFP. In the presence of N, E, and S, M_long_ had a slightly higher colocalization at the ERGIC than M_short_ (Fig. 5b, c). This is in line with the ERGIC as the site of assembly and budding of SARS-CoV-2, M_long_ being the more favorable conformation for budding of SARS-CoV-2, and M_long_ being more predominant in VLPs.

### M-C1P interaction is critical for structural protein trafficking and assembly of virus-like particles

We next asked whether disrupting M-C1P interactions has functional consequences relevant to virus assembly and budding. A cationic patch in the M CTD implicated in C1P interaction by MD (residues 146-RGHLRIAGHH-155) was mutated to replace positively charged arginine and histidine residues with alanine. This mutation resulted in loss of M binding to C1P, assessed with lipid-coated bead pull-down, and is referred to as M^ΔC1P^ (Fig. 5d). A second cationic-rich region (198-RYRIGNYK-205) that was previously shown to be a trans Golgi retention signal for MERS M protein^33^ was also mutated and is referred to as M^ΔTGR^. Similar to the previous report^33^, M^ΔTGR^ showed disrupted M localization and colocalization with S and E (Supplementary Fig. 13a, b). In a similar fashion, M^ΔC1P^ disrupted M localization (Supplementary Fig. 13a, b) and reduced colocalization with S and E (Fig. 5e, f).

Additional M mutants were assessed to probe the impact of disrupting C1P interaction and to validate the specificity of M_short_ and M_long_ antibody labeling. M^ΔTGR^ includes mutations in the M_short,_ but not the M_long_, antibody epitope, and is therefore expected to specifically disrupt M_short_ labeling. Indeed, only the M_l__ong_ antibody was able to detect this mutation (Supplementary Fig. 12i, j). M^P132S^ was recently identified as an M inhibitor escape mutation^26,27^ and is speculated to favor M_long._ As expected, M^P132S^ showed a significantly higher proportion of M_long_ (Fig. 5g, h). Similarly, M^P132W^, which we predict similarly promotes M_long_ based on AlphaFold predicted structures (Supplementary Figs. 9, and 12i, j), showed increased M_long_ labeling. Mutation of direct C1P-interacting residues in (R131A and W110A/R131A) likewise significantly increased the proportion of M_long_ to M_short_ in cells (Fig. 5g, h). In contrast, control mutations R150A and R158A, which do not directly contact C1P based on our structural and MD results, showed an M_long_ fraction indistinguishable from wild-type M (Supplementary Fig. 12). We further tested the consequence of the M^R131A^ mutation by assessing colocalization with S. M^R131A^ showed significantly reduced localization with S in co-transfection experiments (Fig. 5i, j).

**Fig. 6:**
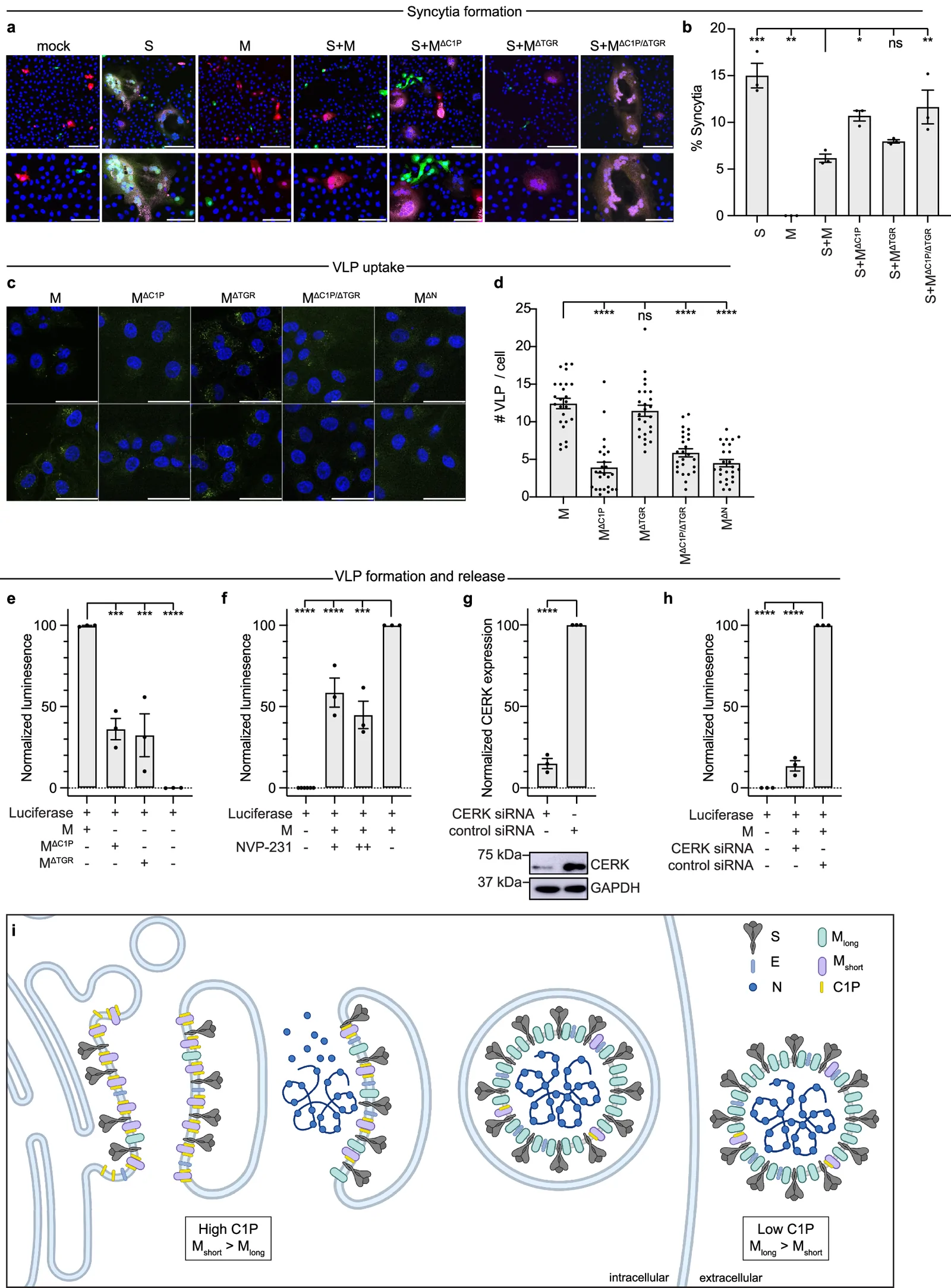
Functional impact of disrupted M-C1P interaction. **a**, **b** Cell syncytia formation assay. **a** Representative images of mCherry-expressing Vero E6 cells (red) combined with hACE2:GFP-expressing cells (green) and transfected with the indicated S and/or M constructs. Nuclei are blue, and Spike is magenta. Images are shown at two scales (scale bar top = 200 µm, bottom = 100 µm). **b** Quantification of syncytia formation assay. Expression of S alone, S and M^ΔC1P^, or S and M^ΔC1P/ ΔTGR^ significantly increased syncytia formation compared to cells expressing S and wild-type M (mean ± SEM, *n* = 3 biological replicates with 2 technical replicates, quantifying 5–10 fields per plate). **c**, **d** Virus-like particle (VLP) entry assay. M^ΔC1P^, M^ΔC1P/ΔTGR^, and M^ΔN^ (which reduces M:N-interaction) derived VLPs show reduced cell entry compared to VLPs formed with wild-type M or M^ΔC1P^. Three biological replicates with ~25 cells analyzed per replicate. Scale bar = 50 µm. **e**, **f**, **h** VLP budding assay. Luminescence is normalized to the signal from wild-type M (luc: negative control luciferase+T20 packing signal only). VLP formation was compared from cells transfected with Luc+T20, S, E, N, and **e** wild-type M, M^ΔC1P^, or M^ΔTGR^, **f** wild-type M, and treated with one (+) or two (++) doses of CERK inhibitor NVP-231 (500 nM at 24 and 43 h, respectively), and **h** wild-type M, and treated with siRNA targeting CERK or scrambled control siRNA. Three biological replicates with three technical replicates each were performed for (**e**–**h**). **g** CERK knockdown efficiency at 72 h assessed by western blot. CERK protein levels were normalized to GAPDH and expressed as a percentage relative to the non-targeting siRNA control (upper). Data are mean ± Sem (three biological replicates) with differences assessed with **b**, **e**, **f**, **h** one-way ANOVA with Dunnett correction, **d** Brown-Forsythe and Welch ANOVA, and **g** unpaired two-tailed *t*-test with Welch’s correction (****p < 0.0001, ***p = 0.0001, **p < 0.01 (*p* = 0.0028 S + M vs. M or 0.0067 S + M vs S + M^ΔC1P/ ΔTGR^), *p = 0.0231). Source data are provided as a Source Data file. **i** Model for lipid control of M conformational dynamics in coronavirus assembly (created in BioRender. Brohawn (2026) https://BioRender.com/m1wt9oo). M protein adopts M_short_ and M_long_ conformations. Ceramide kinase localization to ERGIC membranes results in high C1P concentrations. C1P binds and stabilizes M_short_ in the ERGIC to facilitate recruitment of E and S. Decreasing C1P along the secretory pathway and other potential factors trigger a conformational switch to M_long_ that promotes higher membrane curvature and oligomerization necessary for virus assembly and budding.

To test the hypothesis that the M-C1P interaction is important for proper M–S interactions in cells, we first employed a syncytia formation assay, which has been used to assess the trafficking of S to the plasma membrane of cells^34–36^. S expression alone resulted in robust syncytia formation that was significantly reduced by co-expression with M or M^ΔTGR^ (Fig. 6a, b). In contrast, co-expression of M^ΔC1P^ with S resulted in increased syncytia formation, consistent with disrupted S interaction (Fig. 6a, b). To test the functional relevance of disrupting M-C1P interactions, we assessed VLP entry for wild-type M and M^ΔC1P^-containing VLPs. Wild-type M containing VLPs were able to undergo cell entry (quantified by the number of GFP-positive puncta counted per cell) as were VLPs containing M^ΔTGR^ (Fig. 6c, d). In contrast, VLPs derived from M mutations that displayed altered S/M/E colocalization and reduced C1P binding (M^ΔC1P^) had a significant reduction in cell entry (Fig. 6c, d). This underscores the importance of M-C1P interaction in proper S localization and incorporation into VLPs. Finally, we utilized an established VLP formation assay^37,38^ to quantify the efficiency of VLP formation under different conditions by measuring the activity of co-packaged luciferase. First, we found a significant decrease in the budding efficiency of both M^ΔC1P^ and M^ΔTGR^ relative to wild-type M (Fig. 6e), suggesting reduced M-C1P interaction and interactions with other structural proteins negatively impact VLP assembly. We next assessed the consequence of reducing CERK activity, which is expected to lower C1P levels in cells. CERK inhibition with NVP-231 and CERK knockdown by siRNA both led to significant reductions in VLP formation (Fig. 6f, g). Notably, neither NVP-231 nor siRNA treatment resulted in appreciable cytotoxicity under the conditions tested (Supplementary Fig. 13c, d). Together, these results demonstrate the importance of M-C1P interaction in coordinating VLP budding.

## Discussion

Together, the data presented here support a model for lipid-regulated coronavirus assembly through specific binding and conformational control of SARS-CoV-2 M protein (Fig. 6i). We propose that C1P binding enriches M_short_ in ERGIC and Golgi membranes to facilitate its organization with other structural proteins S, E, and N in early steps of virus assembly. Transition to M_long_ promotes higher-order assembly and virus release and involves loss of C1P through dilution and/or additional promoting factors such as structural protein binding. Several lines of evidence support our model (Fig. 6i). M is in dynamic equilibrium, M_short_ and M_long_^16,19^, with M_short_ energetically favored (Fig. 2)^18^. Specific binding of C1P further stabilizes and compacts M_short_ (Figs. 1–3). C1P is synthesized and enriched in the ERGIC and Golgi membranes^39^, where M organizes the other structural proteins S, E, and N^40^ (Fig.1). Depleting C1P results in mislocalization and higher-order assembly of M (Fig. 1) and reduces budding (Fig. 6). Mutations that disrupt the M-C1P interaction negatively impact structural protein binding, VLP budding, and VLP entry (Fig. 6). Still, we note that there remain several unanswered questions. First, the most important factor(s) promoting transition from M_short_ to M_long_ are unknown. Notably, a recent crystal structure of coronavirus HKU5 M in complex with a C-terminal peptide of HKU5 N suggests preferential N binding to M in the long conformation ^41^, implying N interaction could be a key factor. Second, while we show that disrupting M-C1P interaction reduces VLP formation and subsequent entry, further quantitative assessments of M–N, M–E, and M–S interactions, as well as the timing of S incorporation and assembly, are needed to understand precisely how M-C1P interactions coordinate assembly and budding.

This model provides insight into the mechanism of action of the recently reported small-molecule antivirals JNJ-9676 and CIM-834, the first that directly target M protein^26,27^. Like C1P, both drugs specifically bind and stabilize an M_short_-like conformation, but the inhibitors apparently prevent conformational transition to M_long_. As a result, M is trapped in an early short-like conformation, preventing the formation of mature virus particles. Whether this is because the inhibitors are not diluted during progression through the endomembrane systems or because the complex is insensitive to additional unknown M_long_-promoting factors remains to be determined. One prediction of this model is that additional M_short_-stabilizing molecules, perhaps additionally co-opting the C1P-binding site, are likely to similarly prevent virus formation. In contrast, M_long_ stabilizing small molecules or antibodies, including those potentially patient-derived^42–46^, are predicted to inhibit virus disassembly and successful infection.

Lipid-enveloped viruses hijack host membranes to form sites of virus assembly and give rise to new virus particles. Herein, we demonstrate that SARS-CoV-2 M makes direct interactions with host cell C1P, which is critical for conformational stability of M_short_ and correct assembly of S. This illustrates SARS-CoV-2 uses specific host lipid interactions to mediate formation of virus particles akin to other virus families (e.g., filoviruses, retroviruses, paramyxoviruses, and flaviviruses^47–54^). We acknowledge that the in vitro and computational lipid systems employed have some limitations, including a lack of lipid asymmetry. While lipid asymmetry is likely lacking in a mature virion or VLP, it is fundamental to cellular membranes. Since the leaflet-specific composition of the ERGIC membrane remains incompletely characterized, including for lipids such as C1P, we employed symmetric ERGIC-like bilayers to provide a controlled framework for isolating lipid-dependent effects on M protein conformation.

A number of studies have demonstrated increased cellular levels of ceramide during SARS-CoV-2 infection^55,56^ where ceramide was shown to help facilitate viral entry^57,58^. Further, drugs that disrupted ceramide synthesis (e.g., amitriptyline) reduced SARS-CoV-2 entry into cells^57,59^ and reduced mortality in patients^60^. While the details of C1P in this process have not been explored in great detail, ceramide is the direct substrate of C1P generation, and increases in C1P have been shown in the sera of SARS-CoV-2-infected mice and in infected Vero E6 cells^61^. Thus, more detailed studies examining ceramide/C1P ratio changes in SARS-CoV-2 infection, and cellular models used herein, are warranted.

## Methods

### Molecular dynamics setup

The atomistic model of the M protein was built using the available cryo-EM structures of both the short (PDB ID: 7VGS) and long (PDB ID: 7VGR) conformations^19^. The CHARMM-GUI Membrane Builder^62–65^ was employed to prepare initial MD systems, each consisting of a single M protein in either the short or long conformation embedded in a lipid bilayer. We utilized a 20 × 20 nm lipid bilayer membrane, which is sufficiently large to observe the membrane response of a single M protein. Based on biochemical experiments, we constructed two membrane models that mimic the composition of the endoplasmic reticulum-Golgi intermediate compartment^23,24^. The first system is composed of a mixture of 1,2-dipalmitoleoyl-sn-glycero-3-phosphocholine (DYPC) (45%), 1-palmitoyl-2-oleoyl-inositol (POPI) (20%), 1,2-dipalmitoleoyl-sn-glycero-3-phosphoethanolamine (DYPE) (15%), 1-palmitoyl-2-oleoyl-sn-phosphatidylserine (POPS) (10%), and Cholesterol (10%). The second replaces POPS with N-behenoyl-ceramide-1-phosphate (C1P). The lipid bilayer was constructed as a symmetric membrane, with identical lipid composition in both leaflets. We selected this membrane model to closely replicate the ERGIC membrane properties, ensuring an optimal balance of fluidity, curvature, and bilayer thickness^66–68^. Previously, we utilized this model to investigate lipid–protein interactions involving the SARS-CoV-2 M, N, and E proteins^15,69^.

We solvated the M/lipid-membrane system in a periodic rectangular box filled with TIP3P model water molecules, ensuring that the transmembrane region of the M protein was positioned close to the bilayer core. A multistep minimization and equilibration of the system was carried out following the protocols provided on CHARMM-GUI^70^. We used 0.15 M KCl to neutralize the system. We ran four conditions: M_short_ with C1P, M_short_ without C1P, M_long_ with C1P, and M_long_ without C1P. Each condition was run in triplicate for twelve total MD simulations.

### Simulation details

All atomistic MD simulations were performed using GROMACS 2019 with the CHARMM36m force field^71,72^. The simulations maintained a temperature of 310.15 K using a Nosé-Hoover thermostat^73,74^ with a coupling time constant of 1.0 ps. During initial relaxation, the pressure was maintained at 1 Bar with a Berendsen barostat^75^. Periodic boundary conditions were applied with a time step of 2 fs. For the production run, the Parrinello-Rahman barostat was employed semi-isotropically due to the presence of a membrane^76,77^. A compressibility factor was set at 4.5 × 10^–5^ with a coupling time constant of 5.0 ps. Van der Waals interactions were computed using a switching function between 1.0 and 1.2 nm, and long-range electrostatics were calculated using the Particle Mesh Ewald (PME) method^78^. Hydrogen bonds were constrained using the LINCS algorithm^79^. We performed a 4 µs production run for each replica using Frontera (Texas Advanced Computing Center) and Midway2 (Research Computing Center at the University of Chicago) computing facilities.

### Free-energy calculations

We used metadynamics to explore the free-energy landscape^80^. The Hamiltonian of the system includes a history-dependent Gaussian bias potential, which is a function of a finite set of collective variables (CVs). In this study, we employed the well-tempered metadynamics (WT-MetaD)^25^ technique to investigate the switching between the short and long conformational states of the M protein. In WT-MetaD simulations, the Gaussian bias height is progressively lowered to avoid overfilling the free-energy basins and to achieve convergence^81^.

$$V\left(s,{t}_{n}\right)={\sum }_{i=0}^{n}\frac{h{e}^{-{\left(s-{s}_{{t}_{i}}\right)}^{2}}}{2{\sigma }^{2}{e}^{-\frac{V\left({s}_{{t}_{i}},{t}_{i=1}\right)}{\Delta T}}}$$

where h is the Gaussian height, σ is the Gaussian width, and ${s}_{{t}_{i}}$ are the sequence of points in CV space at time *t*_*i*_. ΔT controls the rate of height decrease.

From manual inspection of superimposed M_short_ and M_long_ structures, we defined two collective variables (CVs) to monitor conformational switching during simulations (Fig. 2a). The first reports the angle between CTDs and is calculated between S108 (subunit A), S184 (subunit A), and S108 (subunit B). This angle in M_short_ and M_long_ structures is 85° and 65°, respectively. The second reports on intersubunit separation and is defined as the distance between R107 in subunit A and subunit B. This distance in M_short_ and M_long_ structures is 4.9 nm and 3.9 nm, respectively (Fig. 2a). We selected the Cα atom of each residue for angle and distance calculations.

In this work, Gaussians were deposited every 500 steps with an initial height set to 0.3 kJ mol^−1^. A bias factor of 15 was used to reduce the Gaussian height. The value was set to 0.1. We conducted WT-metaD simulations using PLUMED 2.8.2 patched with GROMACS 2019 for both the model-C1P and control systems, using the long conformation as the starting structure. The error bars were computed using block averaging, as implemented in PLUMED^82^.

### Analysis

Distances, angles, and the number of contacts were calculated using the GROMACS module^71^. Charged contacts (Supplementary Fig. 4f, g) were analyzed using the MDAnalysis Python package, considering positively charged residues in the CTDs and negatively charged phosphate groups of C1P within a 0.6 nm cutoff. Supplementary Fig. 4f was plotted using MATLAB. Lipid distribution around the M protein, membrane thickness, and mean curvature were also analyzed using the MDAnalysis Python package^83^. Membrane thickness was computed as the distance between the planes formed by phosphate headgroups of the upper and lower leaflets. The residence time of interactions between lipid molecules and protein residues, as well as the occupancy of lipids around the protein, were determined using PyLipID^84^. For movie visualization, we selected eight C1P lipids from the cytoplasmic leaflet in each frame that were closest to the protein, maintaining a constant number of atoms per frame to ensure proper rendering in VMD and ChimeraX. The resulting movies show the redistribution of C1P lipids and their interactions with the M protein. Final visualizations were generated using VMD^85^ and ChimeraX.

### Protein cloning, expression, and purification

The coding sequence for SARS-CoV-2 M protein (Uniprot P0DTC5) was synthesized (IDT, Newark, NJ) and cloned into a vector based on the pACEBAC1 backbone (MultiBac; Geneva Biotech, Geneva, Switzerland) with an added C-terminal PreScission protease (PPX) cleavage site, linker sequence, superfolder GFP (sfGFP), and 7xHis tag to generate the expression construct (SARS-CoV-2 M)-SNS-LEVLFQGP-SRGGSGAAAGSGSGS-sfGFP-GSS-7xHis^18^. Bacmid was generated by transposition in transformed DH10MultiBac cells according to the manufacturer’s instructions.

Sf9 cells were cultured in ESF 921 medium (Expression Systems, Davis, CA), and P1 virus was generated from cells transfected with Escort IV reagent (MilliporeSigma, Burlington, MA) according to the manufacturer’s instructions. P2 virus was generated by infecting cells at a density of 2 million cells/mL with P1 virus at an MOI ~ 0.1. Infection was monitored by fluorescence, and the virus was harvested at 72 h. P3 virus was generated in a similar manner to expand the viral stock. The P2 or P3 viral stock was then used to infect Sf9 cells at 4 million cells/mL at an MOI ~ 2–5. At 72 h, infected cells containing expressed M-sfGFP protein were harvested by centrifugation at 2500 × *g* for 10 min and frozen at –80 °C.

Infected Sf9 cells from 1 L of culture (~15 mL of cell pellet) were thawed in 100 mL of Lysis Buffer containing 50 mM HEPES pH 8.0, 150 mM KCl, 0.5 mM EDTA, 2 mM MgCl_2_, 2 mM DTT. Protease inhibitors (Final Concentrations: E64 (1 µM), pepstatin A (1 µg/mL), soy trypsin inhibitor (10 µg/mL), benzamidine (1 mM), aprotinin (1 µg/mL), leupeptin (1 µg/mL), AEBSF (1 mM), and PMSF (1 mM)) were added to the lysis buffer immediately before use. Benzonase (4 µl) was added after the cell pellet thawed. Cells were then lysed by sonication and centrifuged at 150,000 × *g* for 45 min. The supernatant was discarded, and residual nucleic acid was removed from the top of the membrane pellet using DPBS. Membrane pellets were resuspended in High Salt Lysis buffer (50 mM HEPES pH 8.0, 1 M KCl, 1 mM EDTA, 2 mM DTT) plus protease inhibitors, followed by ultracentrifugation at 150,000 × *g* for 45 min. The supernatant was discarded, membrane pellets were washed again with DPBS, then transferred into a Dounce homogenizer containing extraction buffer (50 mM HEPES, 150 mM KCl, 1 mM EDTA, 1% lauryl maltose neopentyl glycol (LMNG, Anatrace, Maumee, OH), 0.1% cholesteryl hemisuccinate Tris salt (CHS, Anatrace, Maumee, OH), pH 8). A stock solution of 10% LMNG, 1% CHS was dissolved and clarified by bath sonication in 200 mM HEPES, pH 8, prior to addition to the buffer to the indicated final concentration. Membrane pellets were homogenized in extraction buffer, and this mixture (150 mL final volume) was gently stirred at 4 °C for 1.5 h. The extraction mixture was centrifuged at 33,000 × *g* for 45 min and the supernatant, containing solubilized membrane protein, was bound to 7.5 mL of Sepharose resin coupled to anti-GFP nanobody for 1.5 h at 4 °C. The resin was then collected in a column and washed with 100 mL of buffer 1 (20 mM HEPES pH 8.0, 150 mM KCl, 1 mM EDTA, 0.005% LMNG, 0.0005% CHS), 150 mL of buffer 2 (20 mM HEPES pH 8.0, 500 mM KCl, 1 mM EDTA, 0.005% LMNG, 0.0005% CHS), and 25 mL of buffer 1. The resin was then resuspended in 7.5 mL of buffer 1 with 0.5 mg of PPX protease and rocked gently in a capped column for 2 h. M protein was then eluted with an additional 15 mL of buffer 1, spun concentrated to ~1 mL (Amicon Ultra 30 kDa cutoff, Millipore, Tullagreen, Ireland), and loaded onto a Superose 6 Increase column (GE Healthcare, Chicago, IL) on an NGC system (Bio-Rad, Hercules, CA) equilibrated in buffer 1. Peak fractions containing M protein were stored overnight at 4 °C prior to the preparation of Fab complexes.

Coding sequences for complementarity determining regions (CDRs) from conformationally selective antibody fragments^19^ or were grafted into rabbit IgG (short conformation selective) or mouse IgG2a (long conformation selective) Fabs or full antibody sequences, synthesized, and cloned in a pTwist CMV BetaGlobin WPRE Neo vector with C-terminal 7xHis tags on the heavy chain (Twist Biosciences). Fab or antibody sequences were cotransfected using PEI in suspension HEK293T GNTI- cells for expression according to the manufacturer’s instructions. Concentrated antibody-containing supernatant was used directly for immunolocalization. Fab-containing supernatant was subjected to purification and chemical labeling. Supernatants were thawed on ice, buffered with HEPES pH 8.0 at a final concentration of 100 mM, and centrifuged at 21,000 × *g* for 10 min to pellet any precipitated material. Clarified supernatant was incubated with 3 mL His-Pure Ni NTA resin (ThermoFisher Scientific, Rockford, IL) for 1.5 h at 4 °C. The Ni NTA resin was collected in a column and washed with 30 mL each of Cholate buffer (40 mM HEPES pH 8.0, 300 mM KCl, 50 mM Na-cholate, 10 mM Imidazole) and Imidazole Wash Buffer (40 mM HEPES pH 8.0, 300 mM KCl, 20 mM Imidazole). The Fabs were eluted in 4 column volumes of Ni NTA elution buffer (40 mM HEPES, pH 8.0, 300 mM KCl, 200 mM Imidazole). The eluate was spin concentrated to ~1 mL with Amicon Ultra spin concentrator 30 kDa cutoff (Millipore, Tullagreen, Ireland) and loaded onto a Superdex 200 increase (S200) column (GE Healthcare, Chicago, IL) on an NGC system (Bio-Rad, Hercules, CA) equilibrated in SEC buffer (20 mM HEPES pH 8.0, 150 mM KCl, 1 mM EDTA). Fractions containing complete Fabs were pooled, concentrated, and stored at 4 °C for future use.

Fab targeting M_short_ was labeled with Alexa Fluor (AF) 594, and Fab targeting M_long_ was labeled with either AF594 or AF647 in separate batches. All labeling was performed by diluting the Fabs to between 0.5 and 1.25 mg in 500 µL prior to the addition of 1 M NaHCO3 at a pH of 8.3 to 100 mM final. The relevant AF was added at a weight ratio of 1:10 dye:Fab, mixed, and covered for 1 h. Labeled Fab was separated from free dye by running the reaction through a 5 mL HiTrap Desalting column in PBS and collecting the earliest eluting peak. Degree of labeling (DOL) was calculated by recording the absorbance at 280 nm and absorbance at 590 nm (for AF594) or 650 nm (for AF647). DOL of the short conformation Fab-AF594 was 1.0, DOL of the long conformation Fab-AF647 was 2.1, and DOL of the long conformation Fab-AF594 was 3.3. Labeled Fabs were flash frozen in liquid nitrogen and kept at −80 °C until use.

### Site-directed mutagenesis

Plasmids for cellular studies of M, N, E, and S were previously described in Plescia et al.^17^ All mutations of M were generated by Gene Universal Inc. (Newark, DE, USA).

### M-Fab complex formation and purification

The peak fraction from size exclusion chromatography containing pure homodimeric M protein was collected, incubated with purified lcFab or scFab at a 1:2.2 M monomer:Fab molar ratio for 45 min on ice, and run on a Superose 6 increase column in Buffer 1 to separate excess Fabs. Pooled peak fractions containing M:Fab complexes were spin concentrated with an Amicon Ultra 50 kDa cutoff spin concentrator to 2.2 mg/mL (scFab) and 2.7 mg/mL (lcFab). Concentrated complex samples were cleared by a 10-min 21,000 × *g* spin at 4 °C, then d18:1/16:0 ceramide-1-phoshate (C1P; N-palmitoyl-ceramide-1-phosphate ammonium salt, (Avanti Polar Lipids, Alabaster, AL)) was added to each sample to a final concentration of 100 µM from a 1 mM stock in 1:1 MeOH:CHCl_3_. Samples were then incubated on ice for 30 min prior to grid preparation.

### Cryo-EM sample preparation and data collection

3.4 µl samples of M:lcFab or M:scFab in LMNG/CHS and C1P were applied to freshly glow discharged Holey Carbon, 300 mesh R 1.2/1.3 gold grids (Quantifoil, Großlöbichau, Germany) (M:scFab) or C-Flat Au 300 mesh 1.2/1.3 (Electron Microscopy Sciences, Hatfield, PA) (M:lcFab) then plunge frozen in liquid ethane using a FEI Vitrobot Mark IV (ThermoFisher Scientific) with a ~5 s wait time set to 4 °C, 100% humidity, blot force 1, and 3 s blot time.

Grids were clipped, and cryo-EM micrographs were collected on a Titan Krios G3i microscope operated at 300 kV. Fifty-frame videos with a total electron dose of 50 e−/Å^2^ were recorded on a Gatan K3 Summit direct electron detector. The M:scFab dataset was collected in super resolution mode with a pixel size of 0.4055 Å (0.8110 Å physical pixel size), and the M:lcFab dataset was collected in counting mode with a pixel size of 0.848 Å. Videos were recorded around a central hole position via image shift in an 11 × 11 hole pattern with two targets per hole. Target defocus range was −0.5 to −1.8 µm. See Supplementary Table 1 for detailed data collection statistics.

### Cryo-EM data processing

Data processing for both samples was done in cryoSPARC v4.4.1^86^. For M:scFab in LMNG/CHS/C1P 6741, movie stacks were collected, motion-corrected, binned to 0.8110 Å pixel^−^^1^ using Patch Motion Correction, and CTF-corrected using Patch CTF Estimation (Supplementary Fig. 5). Micrographs with an estimated CTF resolution below 7 Å were discarded, leaving 5105 for further processing. Initial particle picking was performed using cryoSPARC’s reference-free blob picker and curated from 404,953 particles down to 29,857 ‘good’ particles by 2D classification.

Topaz training, picking, and extraction yielded 351,588 particles, which were then iteratively 2D classified, resulting in a set of 163,911 ‘good’ particles. A two-class ab initio reconstruction was performed to further sort out 44,905 particles that did not contribute to a high-resolution 3D map. Subsequent non-uniform refinement (C1, 2 extra passes, 14 Å initial resolution) produced a map with an overall 2.94 Å resolution showing clear lipid densities on both leaflets. This map and corresponding 119,006 particles were subjected to local CTF refinement, then global CTF refinement, followed by polishing via Reference Based Motion Correction, which further improved the resolution to 2.7 Å with improved lipid densities. A round of 3D classification (2 classes, 3 Å target resolution, 10 O-EM epochs, 0.95 class similarity) and subsequent C2 non-uniform refinement on the class with the clearest lipid densities produced a final 3.03 Å resolution map (44,726 particles) where a C1P molecule could be unambiguously modeled (Supplementary Fig. 5, Supplementary Table 1). The same non-uniform refinement with C1 symmetry produced a 3.26 Å resolution map with no apparent differences between subunits or lipid binding, so the C2 map was chosen for building an atomic model as the C1P densities were more complete and clearly defined.

The M:lcFab LMNG/CHS/C1P dataset was processed in a similar manner, with 9910 initial micrographs culled to 8432 using a 5 Å cutoff. Topaz was trained on 136,478 particles, yielding a stack of 561,632 particles, which was cleaned, refined, and polished as described for the scFab dataset, yielding 301,406 ‘shiny’ particles. A round of 3D classification yielded a class of 190,251 particles with 3.01 Å final resolution after non-uniform refinement with C2 symmetry (Supplementary Fig. 6, Supplementary Table 1). Further attempts at 3D classification and heterogeneous refinement failed to separate these particles into subsets with unique characteristics or improved map quality.

### Modeling, refinement, and analysis

Modeling of the M:scFab complex in LMNG/CHS/C1P was performed in Coot^87^ using cryoSPARC sharpened cryo-EM maps, the SARS-CoV-2 M protein short conformation structure (PDB ID: 8CTK), and an AlphaFold3^88^ model of the scFab variable domains rigid body fitted into the map in Coot as initial models. The C1P ligands (3-letter code 1PX) were added in Coot. The model was real-space refined in Phenix^89^ and validated using Molprobity^90^. Mutant models were generated with AlphaFold3.

We assessed putative metal coordination using several computational tools. First, AlphaFold3^88^ structure predictions of M dimers with two cations (potassium, sodium, calcium, and magnesium) all show ion occupancy of the putative binding site with minimal distortions to the overall fold. Second, Metric Ion Classification (a deep learning tool for assigning ions in cryo-EM maps^91^) and CheckMyMetal (CMM, an ion validation tool that accounts for ligand chemistry, geometry, and refined B-factor statistics^92^) both predict metal over water binding to this site. We therefore separately modeled each ion in the structure, refined the model in Phenix^89^, and compared validation statistics in CMM. The site was predicted in all cases to be most likely occupied by potassium, and K+ is included in the final models.

### Lipid-binding assays

HEK293 cells were used to express and purify M protein for lipid-binding overlay assays. M was cloned untagged or with an N-terminal HA epitope, N-terminal EGFP, or N-terminal His-SUMO-TEV- M. Eight µg of DNA was diluted into Opti-MEM, mixed with PEI in the ratio of 3:1 (PEI:DNA), and incubated for 10 min at room temperature. The PEI-DNA mixture was added to cells plated in a 60 mm dish at 80% confluency. 5 h post-transfection, the media were changed to DMEM + 10 % FBS. The cells were then incubated for 24 h at 37 °C and 5% CO_2_. Media was then removed from the dish, and cells were washed twice with PBS and lysed with lysis buffer (10 mM HEPES pH 7.4, 150 mM NaCl, 0.25% Igepal CA-630, 5 µM PMSF, 5 mM EDTA, and Halt protease inhibitor cocktail). Cells were incubated in lysis buffer at 4 °C for 10 min. Cells were then scraped from the dish and transferred to a prechilled centrifuge tube. The cell lysate was incubated for 30 min at 4 °C with frequent 30–40 sec vortexing. The cell lysate was then centrifuged at 16,000 × *g* for 10 min at 4 °C to separate the soluble and insoluble fractions. The supernatant was transferred to a microcentrifuge tube, and small aliquots were used to determine the protein concentration with the BCA method. SDS-PAGE followed by Western blot was performed on the purified cell lysates to confirm M protein expression and check expression levels for different conditions/replicates. An anti-M antibody was used for M protein detection, and an anti-actin antibody was used for control blots on cell lysates.

Lipid strips or sphingolipid snoopers were used to detect binding of M protein to different lipids. 0.5 µg of cell lysate was added as a control to one corner of each membrane to ensure M detection in each blot. The membrane was allowed to fully dry after cell lysate blotting. Once dry, the membrane was saturated with 1% milk in TBS-T for 1 h at room temperature. After 1 h, the solution was replaced with 4 µg/ml of total cell lysate in 1% milk in TBS-T and incubated overnight at 4 °C. The membrane was then washed three times for 10 min each with TBS-T at room temperature. The membrane was incubated with anti-SARS-CoV-2 M protein antibody diluted 1:5000 in 1% milk in TBS-T for 1 h at room temperature. The membrane was then washed three times for 10 min each with TBS-T at room temperature. The membrane was then incubated for 1 h at room temperature with secondary antibody (goat-anti-rabbit) diluted at 1:5000 in 1% milk in TBS-T for 1 h at room temperature. M protein interactions with different lipid classes were resolved using ECL Clarity reagents and an imager (Amersham Imager 600) to visualize M protein location on each membrane.

To determine the ability of M protein to interact with lipid-coated beads, a pull-down assay was performed using control beads, C1P beads, and PA beads (Echelon Biosciences, Inc., Salt Lake City, UT). Pull-down assays in Fig. 1 and Fig. 5 were performed in parallel. M or M^ΔC1P^ was expressed in HEK293 cells for 24 h. Cells were collected, lysed as described above, and protein was quantified with the BCA assay. Bead slurry was washed three times (10 mM HEPES pH 7.4, 150 mM NaCl, 0.25% Igepal), collected by centrifugation (1000 × *g*, 5 min), and incubated with cell lysates for 3 h at room temperature. Beads were washed 5 times, and proteins were eluted with 80 µL of 2× Laemmli sample buffer at 100 **°**C for 5 min. SDS-PAGE analysis was performed on the bead pull-down samples, which were followed by a Western blot to detect the M protein in each sample.

### C1P cellular assays

TLC plates were prepared with 20 cm height and width and incubated with 50 of 1:1 chloroform:methanol (v/v) in a glass tank. The TLC plate was secured in a plate holder for stability, and the tank lid was closed until the solution ascended to 0.5 cm from the top of the plate (usually 2–3 h, depending on room temperature). The TLC plate was allowed to dry for 5 min in a fume hood. The TLC plate was then activated for 30 min in an oven at 100 °C. Lipid samples were prepared in chloroform (5–10 µl), sonicated, spun down, and loaded (0.5–2 µl of each sample) ~1 cm from the bottom of the TLC plate. Spots were dried prior to loading the plate in a saturated tank. Plates were sprayed with primuline (0.005% (w/v) in 4:1 acetone:water (v/v)), dried in the fume hood, and imaged at 345 nm (Amersham Imager 600). To assess M protein chromatography profiles with and without inhibition of CERK, cells were treated with vehicle (DMSO) or 100 nM NVP-231 and lysed for analysis of M protein size using a Superdex^TM^ 200 Increase small-scale SEC column (Cytiva Life Sciences, Marlborough, MA). Fractions were collected every 1 mL and a portion of each fraction was spotted onto nitrocellulose to determine M protein content in each fraction via detection of M using Western blot.

### Syncytia formation assay

A syncytia formation assay was modified from ref. ^34^. Approximately 0.8 × 10^5^ Vero E6 cells were seeded per well in a 12-well plate and grown for 24 h. Different respective plasmid constructs, including mCherry, S, M, M^▵C1P^, M^▵TGR^, M^▵C1P/TGR^, hACE2, and GFP, were used to transfect cells using PEI reagent and maintaining the total amount of plasmid (2 μg). Cells were transfected with mCherry alone (mock), S with mCherry, M with mCherry, S and M with mCherry, S and M mutants with mCherry, hACE2, and GFP independently. After 6 h, DMEM was replaced with fresh media. At 24 h post-transfection, cells were trypsinized, and cells from each condition with mCherry expression were mixed with cells transfected with hACE2 and GFP and plated in 35 mm glass-bottom plates. After 24 h, cells were stained for nuclei detection with Hoechst for 15 min at 37 °C and followed by washing with 1× PBS and fixed with 4% PFA. After this, immunofluorescence was done for S protein detection, where cells were incubated with mouse anti-S (1:5000 dilution, Genetex; Cat # GTX632604) for 1 h and then washed with 1X PBS, incubated 1 h with goat-anti-mouse (AlexaFluor 647) secondary antibodies (1:10,000 dilution, Abcam, catalog # ab150115). After washing, cells were stored in PBS before capturing images with a confocal microscope. Vero E6 cells expressing mCherry were used to quantify syncytia formation after combining with hACE2:GFP-expressing cells. 24 h post-transfection of S, S and M, or S with different M mutations, cells were imaged (nuclei: Hoechst 33342 λex 350 λem 461; GFP λex 488 λem 510; mCherry λex 587 λem 610; Spike: AlexaFluor 647 conjugated goat-anti-mouse λex 650 λem 668) and syncytia formation was quantified as the percentage of nuclei within fused cells. Three biological replicates, including two technical replicates, were done for each condition. 5–10 fields were captured for a single plate using a 20× air objective on a Nikon A1si inverted microscope, and the syncytia efficiency was determined as (S/T) × 100, where S is the number of nuclei in the syncytia, and T is the total number of nuclei counted. For analysis, all channels were changed to 8 bit gray, and the DNA channel was duplicated. A threshold was created including all the nuclei, and a watershed feature was used to avoid merged nuclei, and then an analyzed particle count was used to quantify the number of nuclei by setting the particle size (over 50.2 µm).

### VLP budding and entry assays

VLP production was performed and modified from Plescia et al.^17^. Transfections of plasmid DNAs of M:N:S-GFP:E at a 6:3:8:8 ratio in 50 μl Opti-MEM, mixed with PEI in a 3:1 ratio (PEI:DNA), and incubated for 10–15 min at room temperature, and added to cells. Cells were placed at 37 °C and 5% CO_2_, and 5–6 h post-transfection, the media was changed to DMEM + 10% FBS. Transfected cells were incubated at 37 °C and 5% CO_2_. 48 h post-transfection, supernatants were collected in a chilled Falcon tube with 1× Halt^TM^ protease inhibitor cocktail. Cells were washed with 1× PBS and centrifuged 10 min 4 °C at 1000 × *g*. The supernatant was transferred to a new Falcon and centrifuged 10 min, 4 °C at 1000 × *g*, and filtered through 0.45 µm filter into a new tube. Supernatants were deposited on a 20% sucrose cushion. VLPs were centrifuged at 95,050p × *g* for 3 h at 4 °C. The resulting VLPs were used for entry assays and quantified for protein content using a BCA assay. VLP entry assays were performed using equal amounts of VLPs for each condition. Cells subjected to VLPs were spinoculated for 45 min at 4 °C and allowed to incubate for 1 h at 37 °C. Plates were then rinsed with PBS, and nuclei were stained with Hoechst 33342 for 15 min at 37 °C prior to fixation with 4% paraformaldehyde (PFA) and confocal imaging. Quantification of the VLP entry signal was performed by calculating the ratio of VLPs (GFP^+^ puncta) to the total number of cells.

### CERK-M colocalization assay

HEK293 cells were transfected with GFP-CERK and mCherry, Golgi7-mCherry (Human beta-1,4-galactosyltransferase 1 amino acids 1–82 C-terminally tagged with mCherry through a seven amino acid linker (KDPPVAT)), M-mCherry, or M-mCherry with N, E, and S using Lipofectamine 2000. 24 h post-transfection, the cells were stained with 100 nM Hoechst 33342 for 20 min at 37 °C and then imaged with confocal microscopy. Images were analyzed in ImageJ using the Just Another Colocalization Plugin (JACoP). Each condition included three biological replicates with at least six images per replicate.

### M-fab colocalization immunofluorescence assay

HEK293 cells were transfected with M alone or M in combination with N, E, and S alongside fluorescent cell marker GFP, pEmerald-ER3, pEmerald-ERGIC53, pEmerald-Golgi7, pEmerald-LAMP1, or GFP-CERK using Lipofectamine 2000. 24 h post-transfection, the cells were stained with 100 nM Hoechst 33342 for 20 min at 37 °C, washed with PBS, and fixed with 4% paraformaldehyde at room temperature for 10 min. The cells were then washed three times with PBS before blocking and permeabilization with 3% BSA + 0.2% Triton-X100 in PBS for 30 min at room temperature. scFab-AlexaFluor 594 and lcFab-AlexaFluor 647 were diluted to 1 µg/mL in 1% BSA + 0.2% Triton-X100 in PBS and incubated with the cells for 1 h at room temperature. The cells were washed three times with PBS before confocal imaging. Each condition included three biological replicates with at least six images per replicate.

### Quantification of M conformation ratios

HEK293 cells were transfected with WT M or M mutants using Lipofectamine 2000 and were fixed 24 h post-transfection with 4% PFA and stained with Hoechst 33342. Cells were labeled with rabbit IgG anti-M_short_ and mouse IgG2a anti-M_long_ antibodies at a 1:100 dilution, followed by respective secondary antibodies, donkey anti-mouse AlexaFluor 488 (1:1000 dilution, Abcam, catalog # ab150105) and donkey anti-rabbit AlexaFluor 647 (1:1000 dilution, Abcam, catalog # ab150075). Each condition included at least three biological replicates with at least six images per replicate. Individual cells were manually segmented in Fiji/ImageJ by drawing individual cell regions of interest (ROIs), which were adjusted as needed to exclude neighboring cells. The integrated fluorescence intensity for the M_short_ and M_long_ channels was measured separately within each individual cell. Background signal for each channel was corrected by subtracting the mean integrated intensity measured from mock-transfected controls processed in parallel under identical staining and imaging conditions to account for imaging background and non-specific antibody staining. To account for differences in total M expression between cells, the Mlong integrated intensity for each cell was normalized to the total M signal (M_short_ + M_long_) measured within the same cell. The fraction of Mlong was calculated as M_long_/(M_short_ + M_long_).

### M colocalization with E and S

About 70% confluent HEK293 cells in 35 mm glass-bottom plates were transfected with 0.5 μg of each SARS-CoV-2 mCherry-M/mCherry-M^ΔC1P^ /no M, N, GFP-S, Flag-E using Lipofectamine 2000. After 24 h of transfection, cells were stained with 5 μg /ml Hoechst 33342 for 15 min at 37 °C followed by fixing with 4% PFA. After washing with 1× PBS three times, immunofluorescence was done for Flag-E protein detection, where cells were incubated with anti-flag mouse (1:5000 dilution, eBioscience; catalog # 14-6681-82) for 1 h and then followed by washing with 1× PBS, incubated 1 h with goat-anti-mouse (AlexaFluor 647) secondary antibodies (1:10,000 dilution, Abcam, catalog # ab150115). After washing, cells were stored in PBS before capturing images in confocal microscopy. Images were analyzed in ImageJ using the Just Another Colocalization Plugin (JACoP). Each condition included three biological replicates with at least three images per replicate.

### Budding assay

HEK293T cells in a 6-well plate were cotransfected with SARS-CoV-2 structural proteins (S, N, and M or M variants – IRES – E) and a Luc T20 plasmid at a 1:1:1:1 ratio for a total of 2.5 µg of DNA, diluted in 500 µL of Opti-MEM. The transfection mixture was prepared using a 3:1 PEI:DNA ratio, incubated for 15 min, and then added dropwise to HEK293T cells cultured in 1 mL of DMEM supplemented with 10% FBS and 1% Pen/Strep. After 5 h of incubation, the medium was replaced with 1.3 mL of fresh medium. At 48 h post-transfection, the VLP-containing supernatant was collected and passed through a 0.45 µm syringe filter. To assess the effect of CERK inhibition, 500 nM NVP-231 was added either once at 24 h post-transfection or twice at 24 and 43 h post-transfection, and supernatant was collected and filtered at 48 h.

To assess the effect of CERK knockdown, HEK293T cells seeded in a 24-well plate were transfected in Opti-MEM with 10 nM CERK targeting siRNA or control scrambled siRNA (MedChemExpress HY-R502534). Briefly, 0.25 µL of SiRNA diluted in Opti-MEM was mixed with 1 µL of lipofectamine diluted in Opti-MEM and incubated for 25 min at room temperature to allow complex formation. 100 µL of the siRNA-lipofectamine mixture was then added to each well. After 5 h, the medium was replaced with supplemented DMEM. 48 h later, cells were transfected with plasmids encoding SARS-CoV-2 structural proteins (S, N, and M– IRES – E) and a Luc T20 plasmid at a 1:1:1:1 ratio for a total of 500 ng of DNA per well, diluted in 100 µL of Opti-MEM. The transfection mixture was prepared using a 1.5:1 lipofectamine:DNA ratio, incubated for 20 min, and then added dropwise to HEK293T cells cultured in 400 µL of DMEM supplemented with 10% FBS and 1% Pen/Strep, and supernatant was collected and filtered at 48 h. CERK knockdown was confirmed by Western blotting. Cell lysates were collected 72 h post-siRNA transfection. Protein separation was achieved with 10% SDS-PAGE, and proteins were transferred onto PVDF membranes. Membranes were blocked with 5% milk in TBS-T for 30 min before immunostaining with primary antibodies, rabbit anti-CERK (1:5000 dilution, Abcam, catalog # ab155061) or mouse anti-GAPDH (1:10,000 dilution, Invitrogen, catalog # AM4300) overnight at 4 °C. Following washing, blots were incubated with secondary antibodies, goat-anti-rabbit HRP (1:10,000 dilution, Abcam, catalog # ab205718) or sheep anti-mouse HRP (1:10000 dilution, Abcam, catalog # ab6808) for 1 h at room temperature. Blots were imaged using ECL Clarity reagents and quantified using ImageJ software.

Luc-T20 and SARS-CoV-2-M-IRES-E plasmids were a gift from Jennifer Doudna (Addgene plasmid # 177941 and # 177938)^37,38^. Luciferase activity was measured in triplicate by mixing VLP-containing media with Steady-Glo® Luciferase Assay System (Promega) reagent at a 1:1 ratio, for a total volume of 80 µL per well in an opaque white 96-well plate, followed by a 5-min incubation with gentle rocking. Luminescence was quantified using a Synergy NEO2 plate reader with a gain setting of 110 and a 20-s integration time.

To confirm that treatment with 500 nM NVP-231 or CERK knockdown did not affect cell viability, an MTT assay was performed (CyQUANT™ MTT Cell Proliferation Assay Kit). For NVP-231 treatment, cells were seeded and treated in 96-well plates. For CERK knockdown experiments, cells were maintained in 24-well plates as described above. Cell viability was assessed after 48 h of NVP-231 treatment and at 48 h and 96 h post-siRNA transfection.

At the indicated time points, the culture medium was replaced with fresh supplemented DMEM, and MTT solution (12 mM stock) was added to each well (10 µL per 100 µL medium in 96-well plates; 50 µL per 450 µL medium in 24-well plates). Cells were incubated for 3 to 4 h at 37 °C/5%CO_2._ The medium was then carefully removed, and DMSO was added to dissolve the formazan crystals. After thorough resuspension and a 10 min incubation at room temperature, absorbance was measured at 540 nm using a microplate reader.

### SARS-CoV-2 virus assays

SARS-CoV-2/human/USA/WA-CDC-WA1/2020 (courtesy of Dr. Michael S. Diamond, Washington University in St. Louis) was used for all biosafety level 3 (BSL-3) experiments using an A549-hAce2 cell line (from BEI Resources, NIAID, NIH: Human Lung Carcinoma Cells (A549) Expressing Human Angiotensin-Converting Enzyme 2, NR-53821). A549-hAce2 cells were seeded in a black-walled 96-well plate, and all procedures from this point onwards, from SARS-CoV-2 infection to cell fixation, were carried out in BSL-3 using approved protocols. For SARS-CoV-2 infection experiments for antibody staining, samples were arranged into octuplicates where half the wells (4 wells) were mock infected and half the wells (4 wells) were subjected to SARS-CoV-2. Cells were infected at a Multiplicity of Infection (MOI) of 1 by overlay of 50 µl of SARS-CoV-2 inoculum per well (virus diluted in media containing 5% FBS). Mock-infected wells were overlaid with 50 µl of media containing 5% FBS. The infection was allowed to progress for 24 h, when infected cells exhibited mild cytopathic effect (CPE). The cells were fixed as follows: The media was removed, and the cells were washed once with PBS. The cells were incubated with freshly diluted 4% Formaldehyde (1–4 dilution in PBS of Pierce 16% w/v Methanol-free Formaldehyde, ampule-sealed, Catalog # 28908) for 45 min. PBS was added to the wells after dumping the PFA and washing the cells thrice with PBS. The outside of the plate was disinfected with 70% ethanol, and the plate cover was replaced with a fresh one prior to removal from the Biosafety Level 3 laboratory for further processing and imaging. The cells were blocked and permeabilized by submerging the plate in 3% BSA + 0.2% Triton-X100 in PBS for 30 min at room temperature. scFab-AlexaFluor 594 and lcFab-AlexaFluor 647 were diluted 1 µg/mL in 1% BSA + 0.2% Triton-X100 in PBS with 100 nM Hoechst 33342 and incubated with the cells for 1 h at room temperature. The cells were then washed three times with PBS before proceeding to confocal microscopy.

### Cellular colocalization analysis

Confocal microscopy images were analyzed in ImageJ/Fiji using a custom macro script, which performed the following steps: background subtraction from each fluorescence channel using the image median, contrast enhancement with 0.1% saturation for improved visualization, and automatic thresholding using the “Default dark” method. Colocalization was then assessed using the Just Another Colocalization Plugin (JACoP) to calculate Pearson’s correlation coefficients and Manders’ overlap coefficients.

### Deuterium exchange

A summary of HDXMS experiments is provided in Supplementary Table 2.

Labeling buffer was prepared by diluting 10x HEPES buffer in D_2_O (99.9%) (1× buffer: 20 mM HEPES, 150 mM KCl, 1 mM EDTA, pH 8.0). Deuterium labeling was carried out for M protein in detergent as well as M protein mixed with scFab, lcFab, and C1P for 1, 10, and 100 min labeling at 20 °C using a PAL-RTC (Leap) autosampler. M protein was mixed with scFab or lcFab in a 1:1 ratio for a final concentration of 15 μM M dimer for 60 min on ice prior to labeling. M protein (15 μM dimer) was mixed with excess (100 μM) C1P for 30 min on ice prior to labeling. 3 μL of M protein or M protein complex was mixed with 57 μL of labeling buffer for a final D_2_O concentration of 85.4%. After labeling, an equivalent volume of prechilled quench solution (1.5 M guanidine hydrochloride, 0.25 M TCEP, 2.4 mM DDM) was added to the labeling reaction to bring the pH to 2.5.

Deuterium labeling was also carried out on the M protein embedded in VLPs. Labeling buffer was prepared by diluting 20× TNE (20 mM Tris, 100 mM NaCl, 1 mM EDTA) buffer in D_2_O (99.9%). 20 μL of VLP sample was mixed with 40 μL of labeling buffer prior to quenching. After labeling, 3 volumes of labeling reaction were mixed with 1 volume of prechilled quench solution (4 M guanidine hydrochloride, 0.4 M TCEP, 2.4 mM DDM). For comparison, apo M protein in detergent was also labeled after diluting 3 μL of the sample (15 μM dimer) with 17 µL of 1× TNE buffer prior to mixing with 40 µL of labeling buffer to match the final D_2_O concentration of 63.3% in labeled VLPs.

Undeuterated reference samples for VLPs and M protein were prepared by mixing an equivalent sample with 1× buffer in H_2_O prior to quenching. All reference states and labeling timepoints were collected in triplicate.

### Mass spectrometry and peptide identification of recombinant M protein and M complexes

Approximately 15–20 pmol of each protein were digested online using a nepenthesin-2/pepsin mixed bed column (2.1 × 20 mm) (Affipro, AP-PC-006) at 15 °C. Peptides were trapped for 3 min in a C18 trap column (ACQUITY BEH C18 VanGuard Pre-column, 1.7 µM, Waters, Milford, MA) at a flow rate of 100 μL/min. Peptides were subsequently eluted in an acetonitrile gradient (5–35%) over 7 min in 0.1% formic acid on a reverse-phase C18 column (AQUITY UPLC BEH C18 Column, Waters, Milford, MA) at 40 μL/min. All fluidics were controlled by nanoACQUITY Binary Solvent Manager (Waters, Milford, MA). Electrospray ionization mode was utilized, and ionized peptides were sprayed onto a SYNAPT XS mass spectrometer (Waters, Milford, MA) acquired in HDMS^E^ Mode. Spectra were recorded over a range of 50–2000 m/z with the following settings: capillary voltage: 2.8 kV; trap collision energy: 6 V; sampling cone: 30 V; transfer CE ramp 20–45 V; source temperature: 80 °C; and desolvation temperature: 175 °C. Ion mobility settings of 600 m/s wave velocity and 197 m/s, transfer wave velocity were used with collision energies of 4 and 2 V for trap and transfer, respectively. 100 fmol/μL of [Glu^1^]-fibrinopeptide B ([Glu^1^]-Fib) with a flow rate of 5 µL/min was used to inject as lockspray reference mass.

Peptides corresponding to M protein were identified from mass spectra corresponding to undeuterated controls through searches against an M protein database using PROTEIN LYNX GLOBAL SERVER (PLGS) version 3.0 (Waters, Milford, MA) software in HDMS^E^ mode for non-specific protease cleavage. PLGS search parameters were changed to include no fixed or variable modifier reagents and no N-linked glycosylation.

Deuterium exchange was quantified using DynamX v3.0 (Waters, Milford, MA) with cutoff filters of: minimum intensity = 1500; minimum peptide length = 4; maximum peptide length = 30; minimum products per amino acid = 0.11; minimum consecutive products = 1; and precursor ion error tolerance <10 ppm. Only peptides that fulfilled the above-described criteria in 2 of the 3 undeuterated samples were included in the final peptide list.

### Mass spectrometry and peptide identification of M protein in virus-like particles

HEK293 cells were used to produce VLP samples for deuterium exchange analysis. HEK293 cells were seeded in 100 mm round dishes and transfected (at ~70–80% cell confluency) with pcDNA3-M, pcDNA3-N, pCMV-E, and pCMV-S at a ratio of 1:1:1:2 using a 3:1 ratio of polyethyleneimine hydrochloride (PEI) to DNA plasmid. The media was collected 48 h post-transfection and mixed with 0.1× HaltT^M^ Protease Inhibitor Cocktail (Thermo Fisher Scientific). The samples were centrifuged at 1000 × *g* for 10 min at 4 °C, and the supernatant was collected and transferred to a new centrifugation tube for further centrifugation at 2000 × *g* for 10 min at 4 °C. The supernatant was collected and loaded onto a 20% sucrose cushion in TNE buffer (50 mM Tris-HCl, 100 mM NaCl, 0.5 mM EDTA, pH 7.4) for centrifugation at 100,000 × *g* in a Beckman Type 70 Ti rotor for 3 h at 4 °C. The VLP pellet was dried and gently resuspended in TNE buffer and stored at –20 °C prior to shipping. When shipped (typically the next day), samples were shipped at 4 °C, flash frozen, and stored at –80 °C prior to the deuterium exchange analysis.

VLP samples containing M, E, N, and S proteins were digested online at 15 °C using an AffiPro pepsin column (AffiPro, AP-PC-001). All chromatographic components were housed in the UPLC system’s cooling chamber, maintained at 0.0 ± 0.1 °C throughout the measurements. Peptides were trapped and desalted on a VanGuard Pre-Column trap (2.1 mm × 5 mm, ACQUITY UPLC BEH C18, 1.7 μm (Waters, Milford, MA)) for 3 min at a flow rate of 100 μL/min. Elution was achieved using a 5%–35% acetonitrile gradient over 10 min at 100 μL/min, with separation on an ACQUITY UPLC HSS T3 column (1.8 μm, 1.0 mm × 50 mm (Waters, Milford, MA)). The system operated at an average back pressure of ~12,950 psi under initial conditions (5% acetonitrile, 95% water, 0.1% formic acid) at 0 °C. Deuterium level determination had an error margin of ±0.25 Da in this experimental setup. To avoid peptide carryover, a wash solution (1.5 M guanidinium chloride, 0.8% formic acid, and 4% acetonitrile) was injected over the pepsin column after each analytical run.

Mass spectra were acquired on a Waters SELECT SERIES Cyclic IMS instrument coupled with a UPLC I-Class system and HDX manager. Spectra were recorded over an m/z range of 50–2000 with the following settings: capillary voltage, 3.0 kV; trap collision energy, 4 V; sampling cone, 40 V; transfer CE ramp, 15–50 V; source temperature, 80 °C; and desolvation temperature, 450 °C. Ion mobility separation was performed with a single pass using a sequence of 10 ms injection, 3 ms separation, and 34 ms ejection/acquisition. For post-acquisition mass accuracy correction, [Glu^1^]-Fib (Sigma, Burlington, MA) was infused at 250 fmol/μL at 5 μL/min as a lock mass.

To analyze cyclic IMS data, PLGS and DynamX were modified as described^93^.

### Hydrogen–deuterium exchange analysis

Peptides were manually analyzed for quality across technical replicates using DynamX v3.0 (Waters, Milford, MA). The average number of deuterons exchanged in each peptide was calculated by subtracting the centroid mass of the undeuterated reference spectra from each deuterated spectrum. Mass spectral envelopes were acquired from DynamX v3.0 and plotted in MATLAB 2023b (The MathWorks Inc, Natick, MA, USA). Spectra showing bimodal hydrogen–deuterium exchange kinetics were quantified using HX-express3 v39^94^ with iterative fitting applied^95^. Heatmaps representing relative fractional deuterium uptake and deuterium exchange differences were generated in Dynamx v3.0 and mapped onto M protein structures (PDB IDs: 7VGR and 7VGS) in ChimeraX 1.8^96^.

Figures were prepared using VMD, PyMOL, ChimeraX, Prism, Python, MATLAB, and Adobe Illustrator software.

### Reporting summary

Further information on research design is available in the Nature Portfolio Reporting Summary linked to this article.

## Supplementary information

Supplementary Information

Description of additional supplementary information

Supplementary movie 1

Supplementary movie 2

Supplementary movie 3

Supplementary movie 4

Reporting summary

Transparent Peer Review file

## Source data

Source data

## Data Availability

The final cryo-EM maps and atomic models of M:scFab + C1P and M:lcFab + C1P generated in this study have been deposited in the Electron Microscopy Data Bank under accession codes EMD-45919 [https://www.ebi.ac.uk/emdb/EMD-45919] and EMD- 45921 and in the PDB under accession codes 9CTU and 9CTW, respectively. HDXMS data have been deposited in the ProteomeXchange Consortium via the PRIDE partner repository with accession number PXD079401 [https://www.ebi.ac.uk/pride/archive/projects/ PXD079401]. Source data are provided as a Source Data file. Source data are provided with this paper.

## Code availability

The initial structures, GROMACS input files for the atomistic simulations, and PLUMED scripts used in this study are available at https://zenodo.org/records/20647068. The analysis scripts used for data processing are available at: https://github.com/mandirad/Direct-lipid-interactions-control-SARS-CoV-2-M-protein-conformational-dynamics-and-virus-assembly.
