# Supplementary material for: Direct lipid interactions control SARS-CoV-2 M protein conformational dynamics and virus assembly
Source: Nat Commun. 2026 Jul 23;17:8968. doi: 10.1038/s41467-026-75229-9 (PMC13503762; doi:10.1038/s41467-026-75229-9)
Supplement: Supplementary file 1 [file 41467_2026_75229_MOESM1_ESM.pdf]

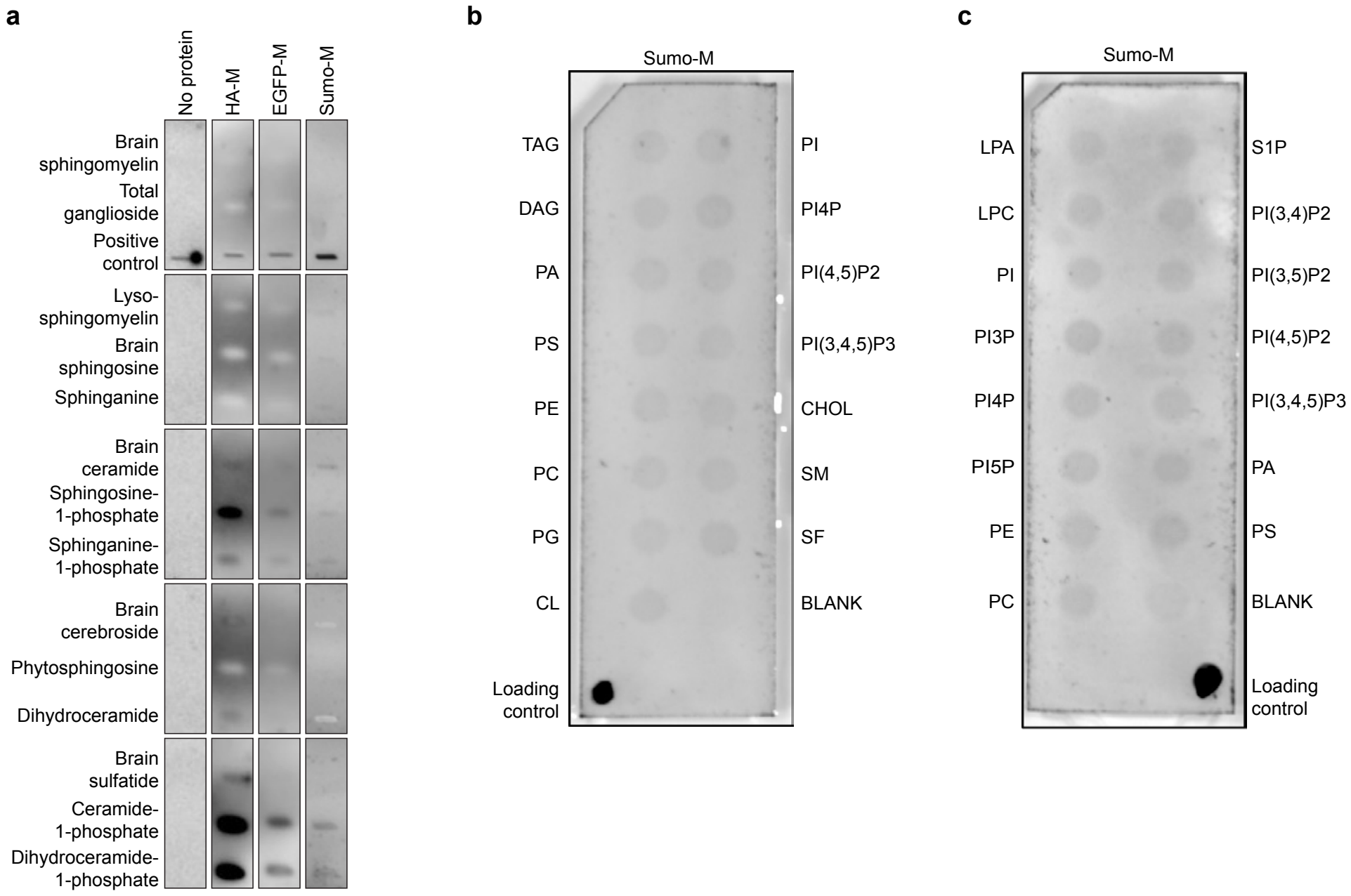

Supplementary Figure 1. M protein-lipid interactions.
(a) Sphingolipid, **(b)** lipid, and **(c)** phosphatidyl inositide snooper assays for M-lipid interactions. **(a)** Western blots of immobilized lipids probed with N-terminal HA-tagged M, a 50:50 mixture of N-terminal EGFP-tagged:untagged M, N-terminal His-SUMO-TEV- M, or a control without M protein. All M constructs tested displayed strong binding to C1P and dihydroC1P and weaker binding to anionic So1P and Sa1P. **(b,c)** Western blots of immobilized lipids probed with Sumo-tagged M. No clear evidence of binding was observed. All western blots were performed with anti-M antibody and secondary conjugated to HRP. Portions of **(a)** are reproduced from Fig. 1a.

a

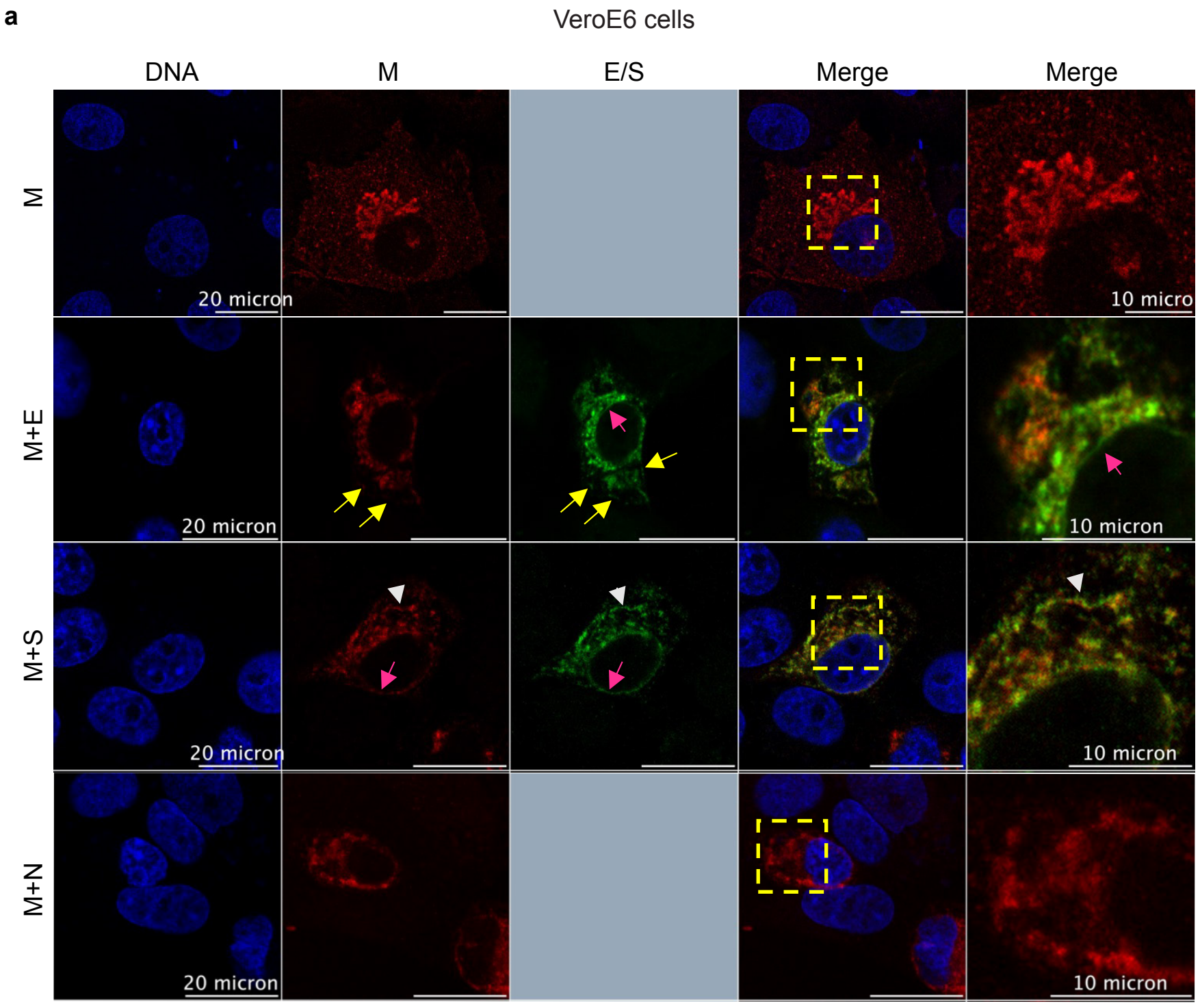

Supplementary Figure 2. SARS-CoV-2 M colocalizes with S and E proteins.
Vero E6 cells were used to assess M protein localization when co-expressed with E, S, or N. The insets and arrows show regions of colocalization. Scale bar = 20 µm, inset scale bar (right column) = 10 µm.

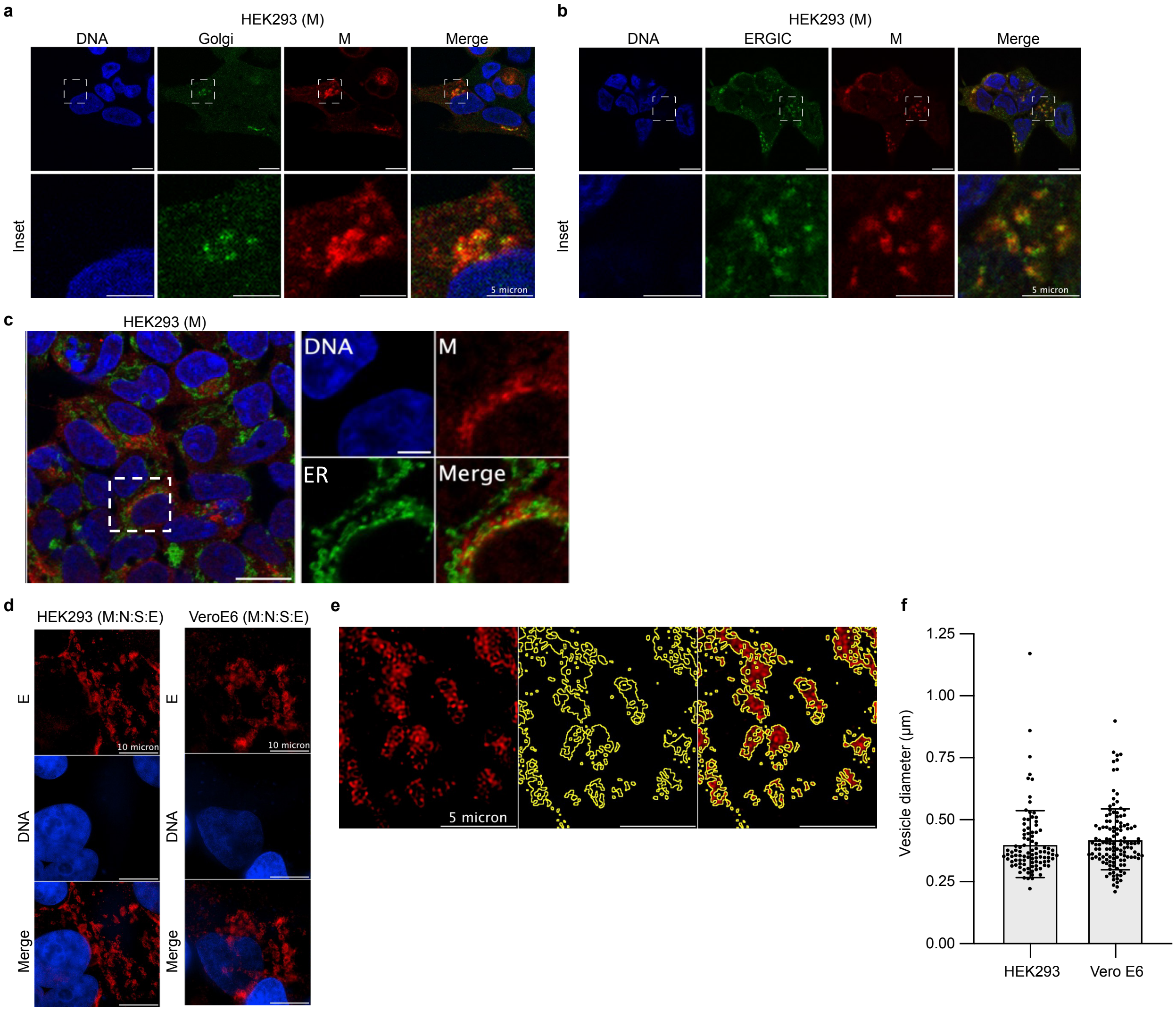

Supplementary Figure 3. SARS-CoV-2 M colocalizes with Golgi and ERGIC markers and SARS-CoV-2 structural proteins co-expressed in mammalian cells exhibit similar vesicle morphology to SARS-CoV-2 infected cells.

(a) M protein (red) expressed in HEK293 cells colocalized with the Golgi marker GORASP2 (green). **(b)** M protein (red) expressed in HEK293 cells colocalized with the ERGIC marker ERGIC-53 (green). **(c)** M protein (red) expressed in HEK293 cells did not appreciably colocalize with an ER marker (green). **(d)** SARS-CoV-2 structural proteins (S, M, Flag-E, and N) were co-expressed in HEK293 or Vero E6 cells and imaged with structure illuminated microscopy using a Nikon N-STORM/N-SIM/TIRF imaging system. Immunofluorescence with labelled anti-Flag antibody for detection of the Flag-E protein (red). **(e)** A zoomed in portion of **(d)** is shown to demonstrate how vesicle diameter was quantified. **(f)** Quantification of vesicle diameter performed using ImageJ. Similar average vesicle diameters illuminated with E-Flag antibody were found in HEK293 and Vero E6 cells. Difference assessed with unpaired t test with Welch's correction, $p=0.276$.

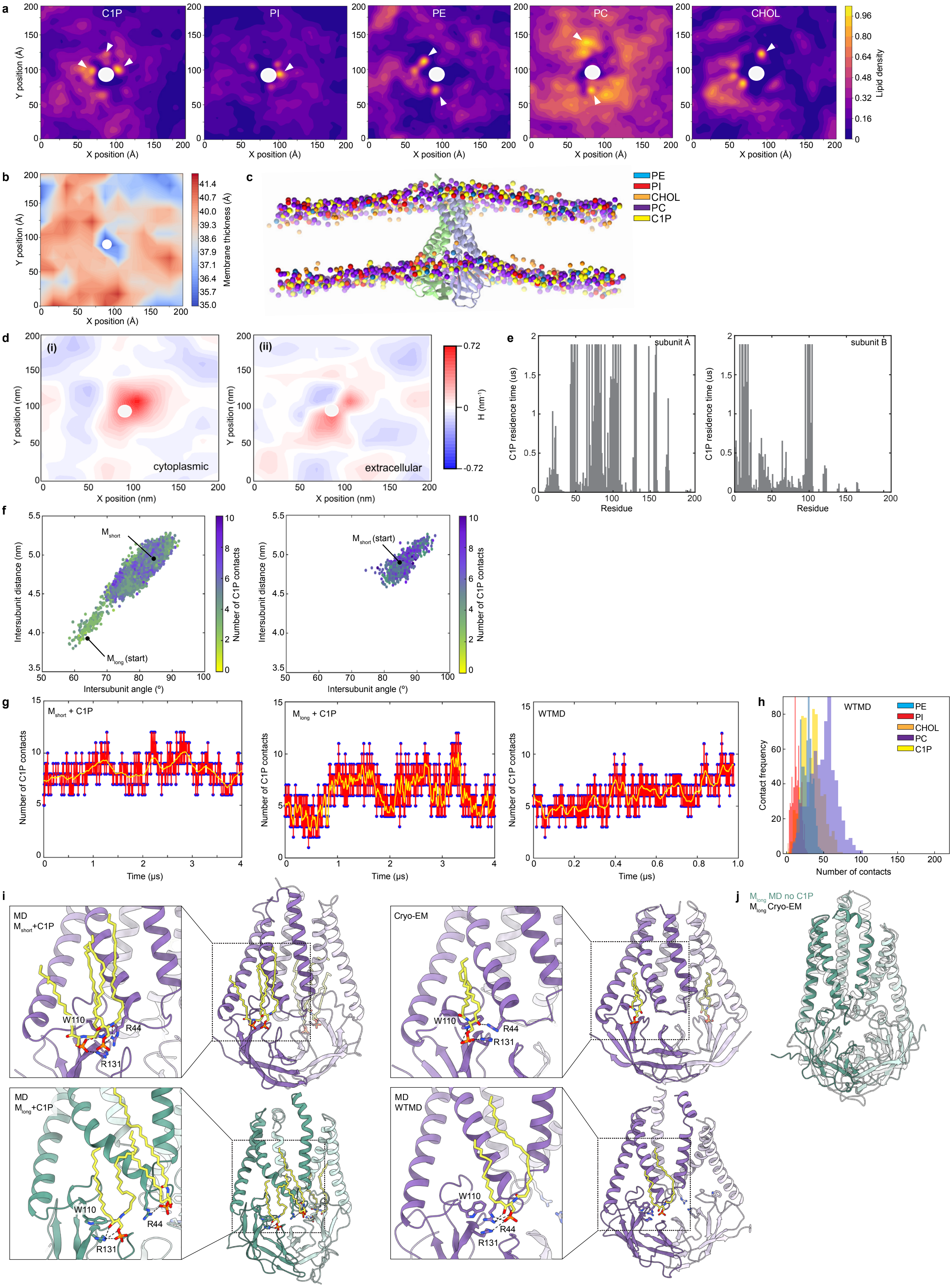
a
C1P
PI
PE
PC
CHOL
Y position (Å)
X position (Å)
Lipid density
b
Membrane thickness (Å)
c
PE
PI
CHOL
PC
C1P
d
(i)
(ii)
cytoplasmic
extracellular
Y position (nm)
X position (nm)
H (nm⁻¹)
e
subunit A
subunit B
C1P residence time (us)
Residue
f
M short
M long (start)
M short (start)
Intersubunit distance (nm)
Intersubunit angle (°)
Number of C1P contacts
g
M short + C1P
M long + C1P
WTMD
Number of C1P contacts
Time (μs)
h
WTMD
Contact frequency
Number of contacts
i
MD
M short+C1P
W110
R44
R131
Cryo-EM
MD
M long+C1P
MD
WTMD
j
M long MD no C1P
M long Cryo-EM

Supplementary Figure 4. Lipid density maps, membrane response, C1P residence time, contact analysis, and binding modes from molecular dynamics simulations.

(a) Spatial density distribution of C1P, POPI, DYPE, DYPC, and cholesterol in the M_{short}+C1P simulation box. Arrowheads indicate regions of higher lipid accumulation. **(b)** Membrane thickness calculated from the last 2 μs of trajectories for the M_{short}+C1P system. **(c)** Side view of the simulated M_{short} system showing membrane thinning around the M protein. **(d)** Mean curvature of the membrane mapped onto the XY plane for inner **(i)** and outer **(ii)** leaflets, showing small deformations around M from a nearly flat surface. White circles in all plots indicate the location of the M protein. **(e)** C1P residence time per residue in subunits A and B in M_{short}+ C1P simulation. **(f)** Intersubunit angle versus intersubunit distance calculated at each time point colored by the number of contacts between negatively charged phosphate groups of C1P and positively charged CTD residues of the M protein dimer. **(g)** Number of C1P contacts between negatively charged phosphate groups of C1P and positively charged CTD residues versus time in M_{short}+C1P, M_{long}+C1P, and WT MD systems. Red lines represent averages across three independent simulations and yellow lines show corresponding running averages. **(h)** M-lipid contact frequency from WTMD simulations with C1P. **(i)** Comparison of M-C1P binding modes observed in cryo-EM structure and snapshots from MD simulations. **(j)** Overlay of M_{long} structure from cryo-EM and MD simulation without C1P.

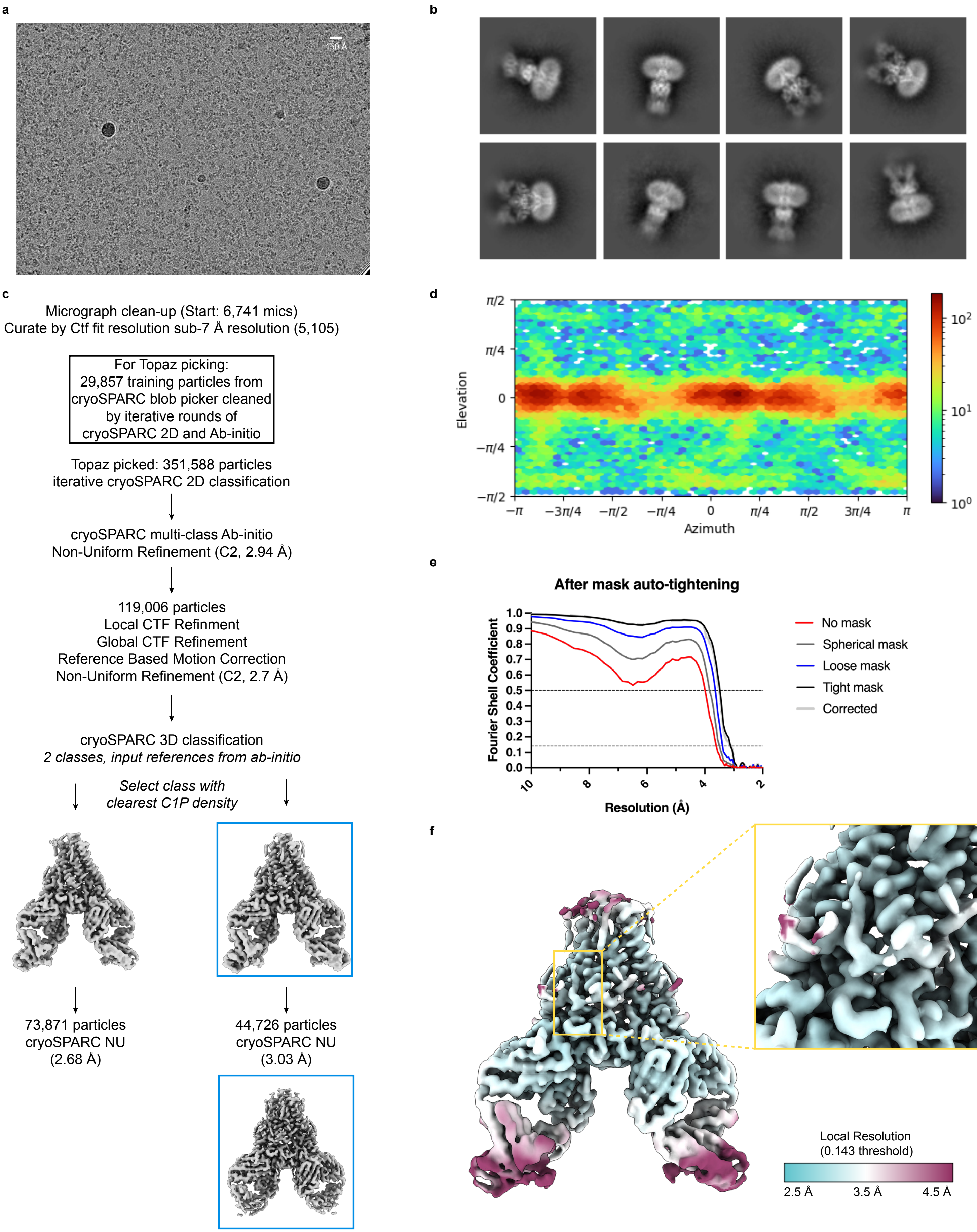

Supplementary Figure 5. Cryo-EM data, processing pipeline, and validation for M_{short}-C1P.
(a) Representative micrograph, **(b)** selected 2D class averages, **(c)** cryo-EM data processing pipeline, **(d)** view angle distribution, **(e)** Fourier shell correlation (FSC) versus resolution between half maps from final non-uniform refinement, and **(f)** local resolution drawn on final sharpened map with inset zoomed in on C1P binding site.

a

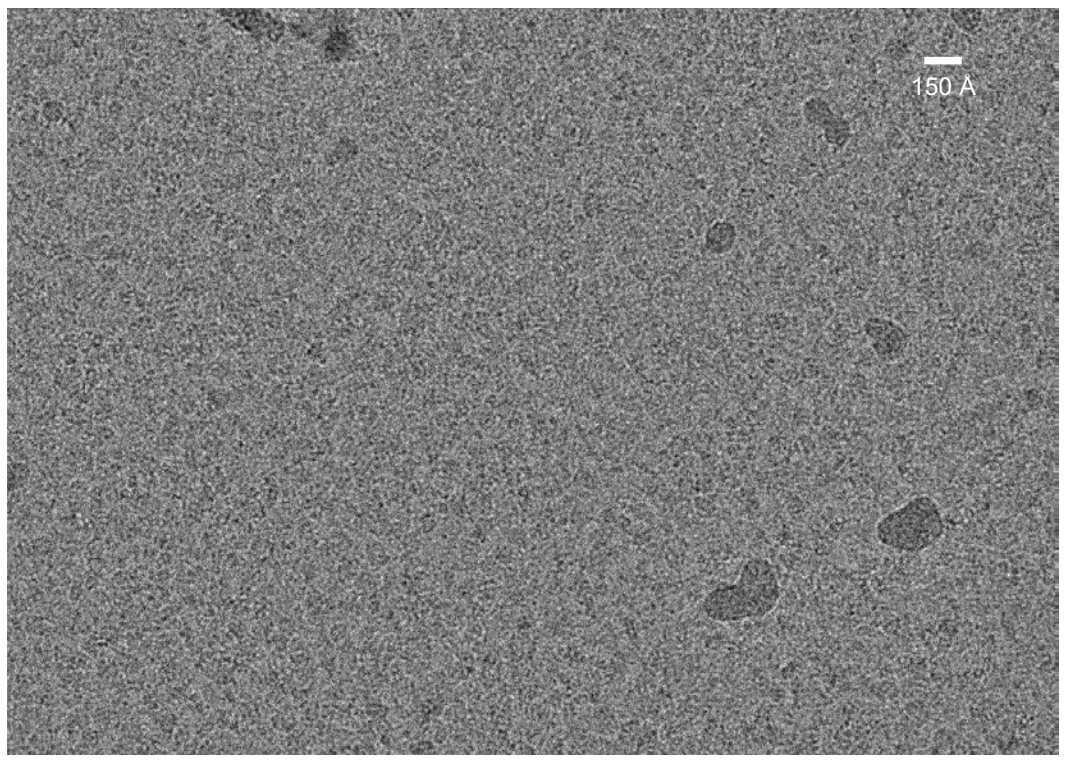

b

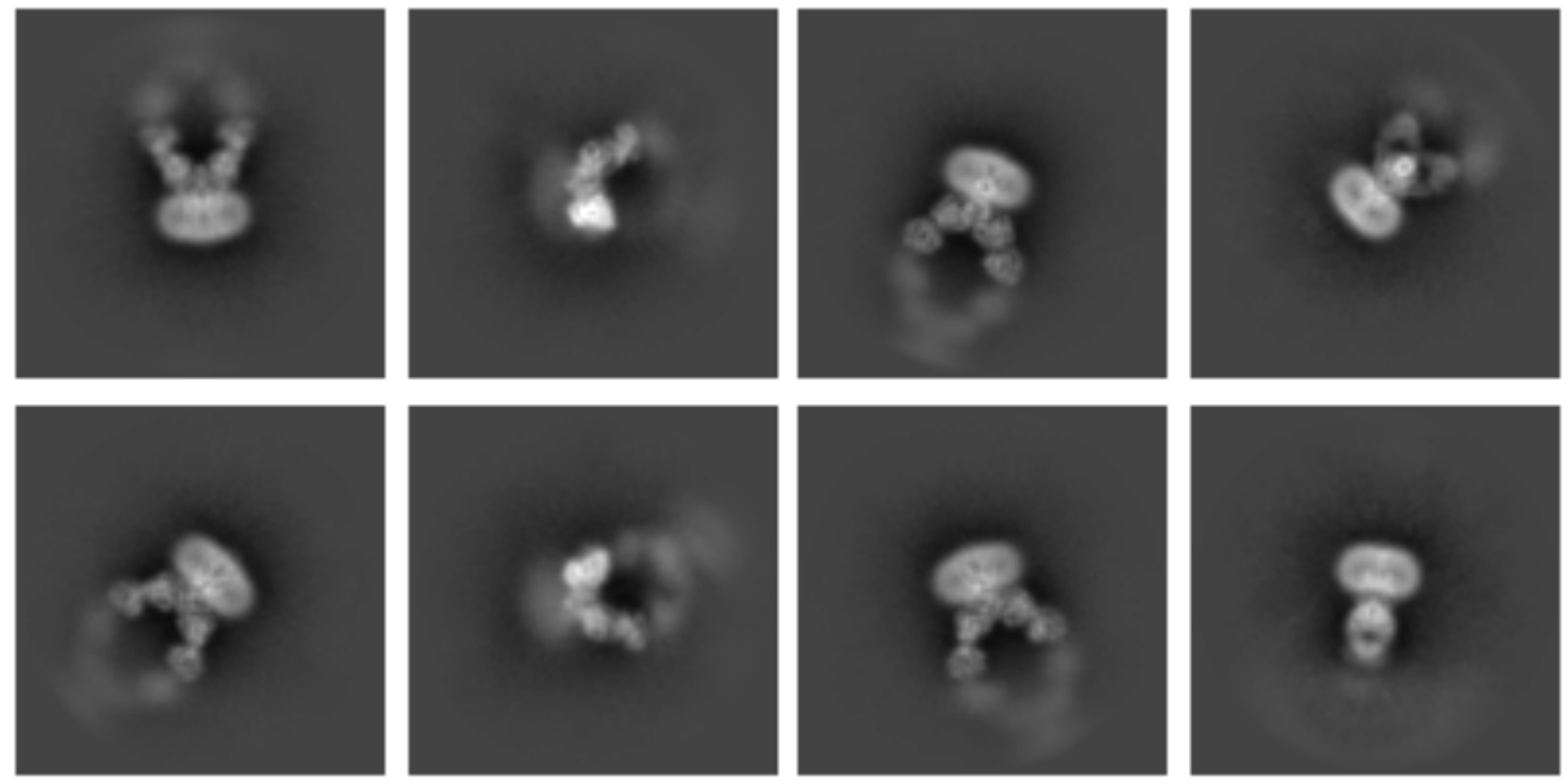

c

Micrograph clean-up (Start: 9,910 mics)
Curate by Ctf fit resolution sub-5 Å resolution (8,432)

For Topaz picking:
136,478 training particles from cryoSPARC blob picker cleaned by iterative rounds of cryoSPARC 2D and Ab-initio

Topaz picked: 561,632 particles
iterative cryoSPARC 2D classification

↓

cryoSPARC multi-class Ab-initio
Non-Uniform Refinement (C1, 3.47 Å)

↓

301,406 particles
Local CTF Refinment
Global CTF Refinement
Reference Based Motion Correction
3D classification

↓

190,251 particles
Non-Uniform Refinement (C2, 3.01Å)

d

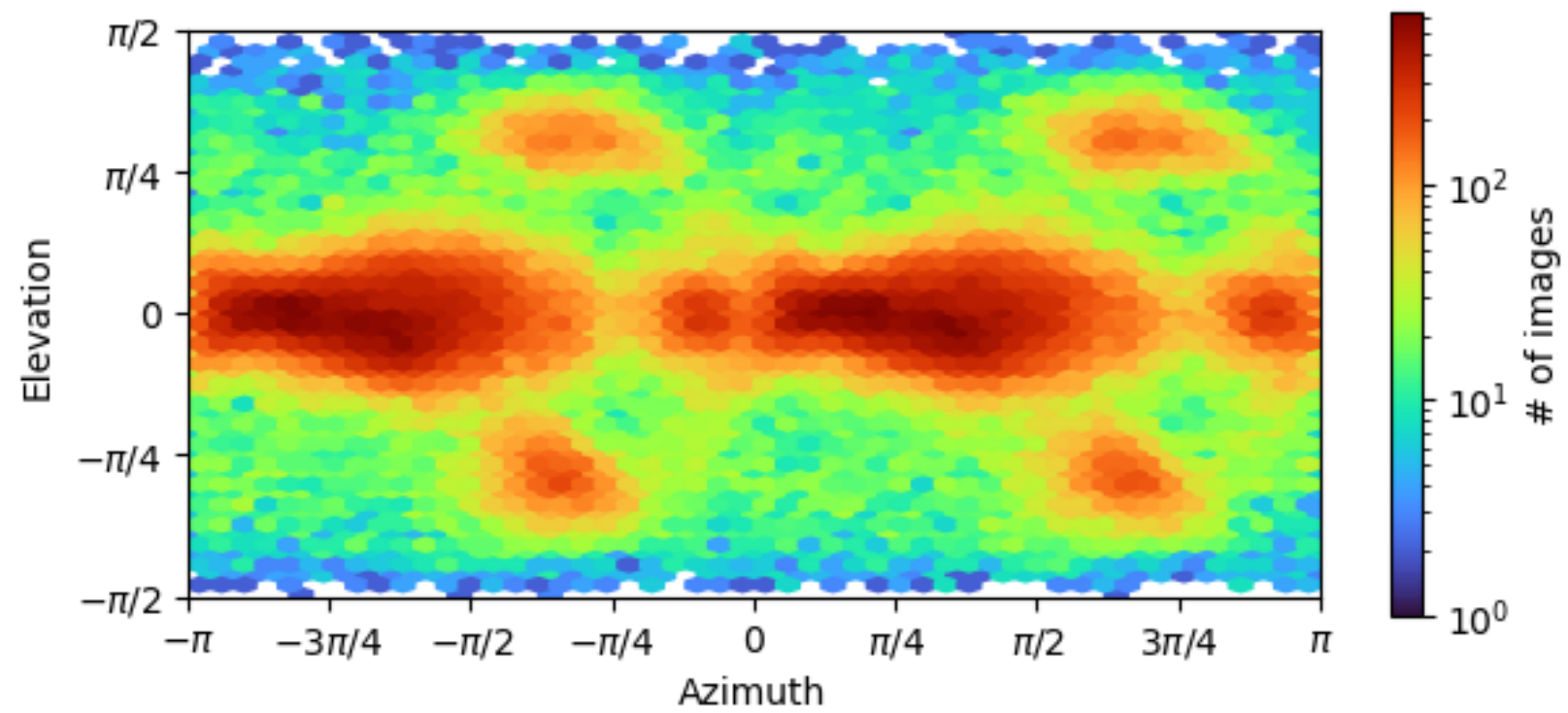

e

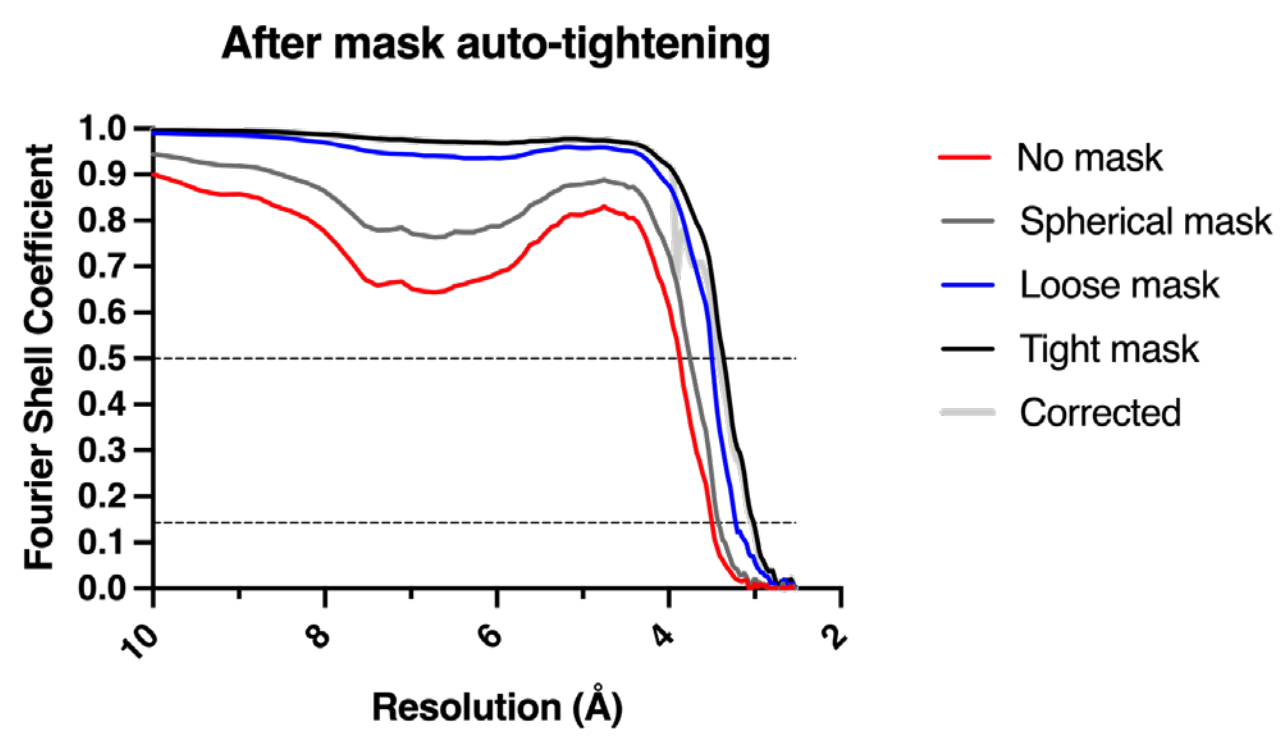

f

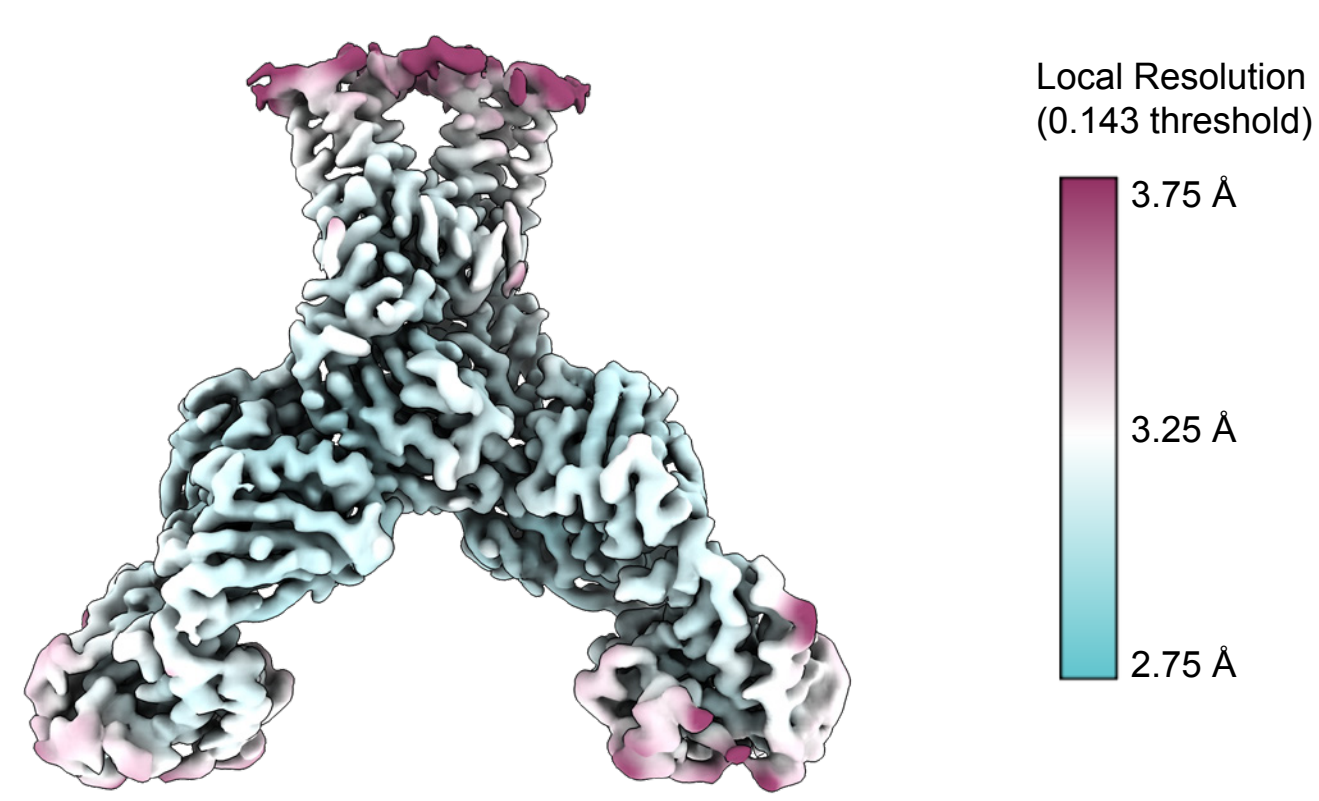

Supplementary Figure 6. Cryo-EM data, processing pipeline, and validation for M_{long} in the presence of C1P.
(a) Representative micrograph, **(b)** selected 2Dclass averages, **(c)** cryo-EM data processing pipeline, **(d)** view angle distribution, **(e)** Fourier shell correlation (FSC) versus resolution between half maps from final non-uniform refinement, and **(f)** local resolution drawn on final sharpened map.

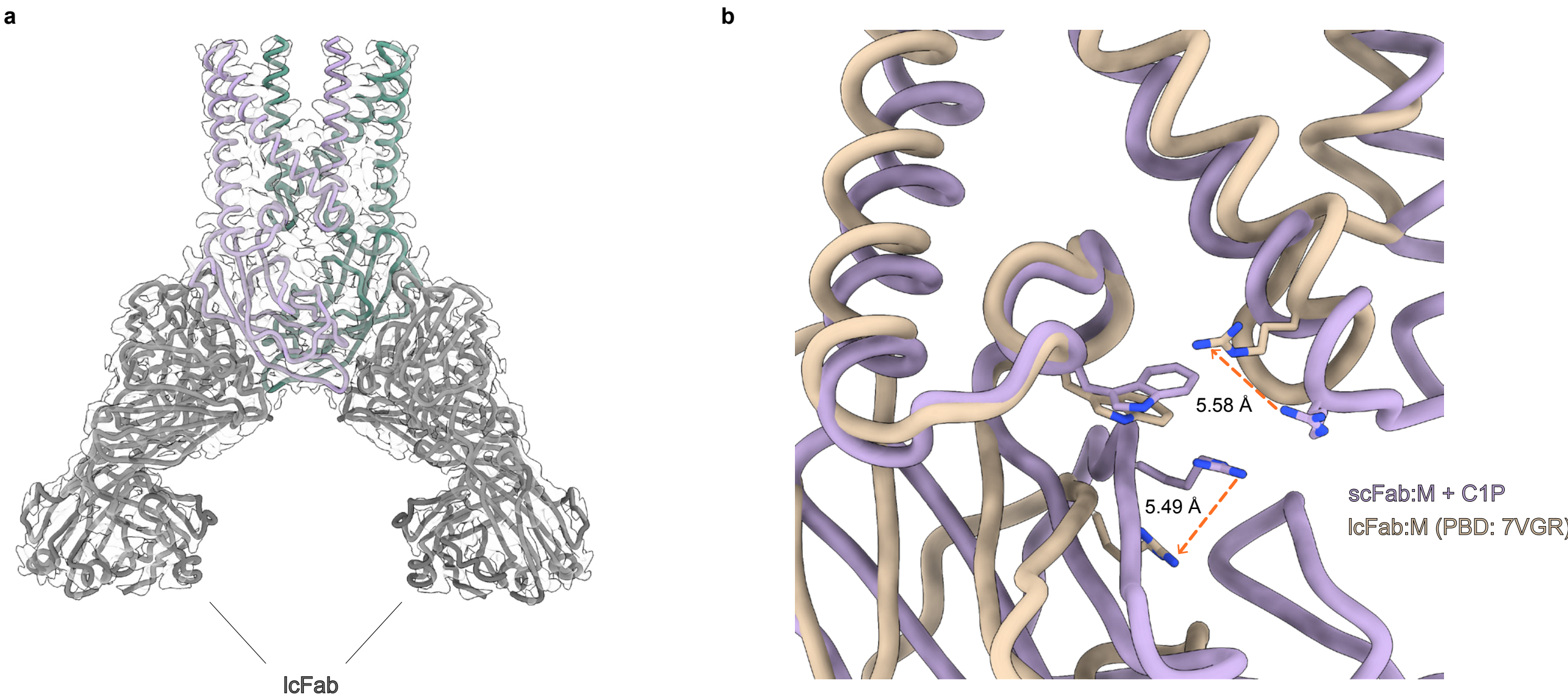

Supplementary Figure 7. Structure of M_{long} in the presence of C1P.
(a) Final model M in the long conformation viewed from the membrane. M subunits are purple and green and long conformation-selective Fabs are gray. 3.0 Å resolution sharpened map is shown as a transparent surface. **(b)** Overlay of M_{long} + C1P and M_{short}-C1P structures highlighting rearrangement of the C1P binding site observed in M_{short} including relative displacement of key C1P-interacting residues.

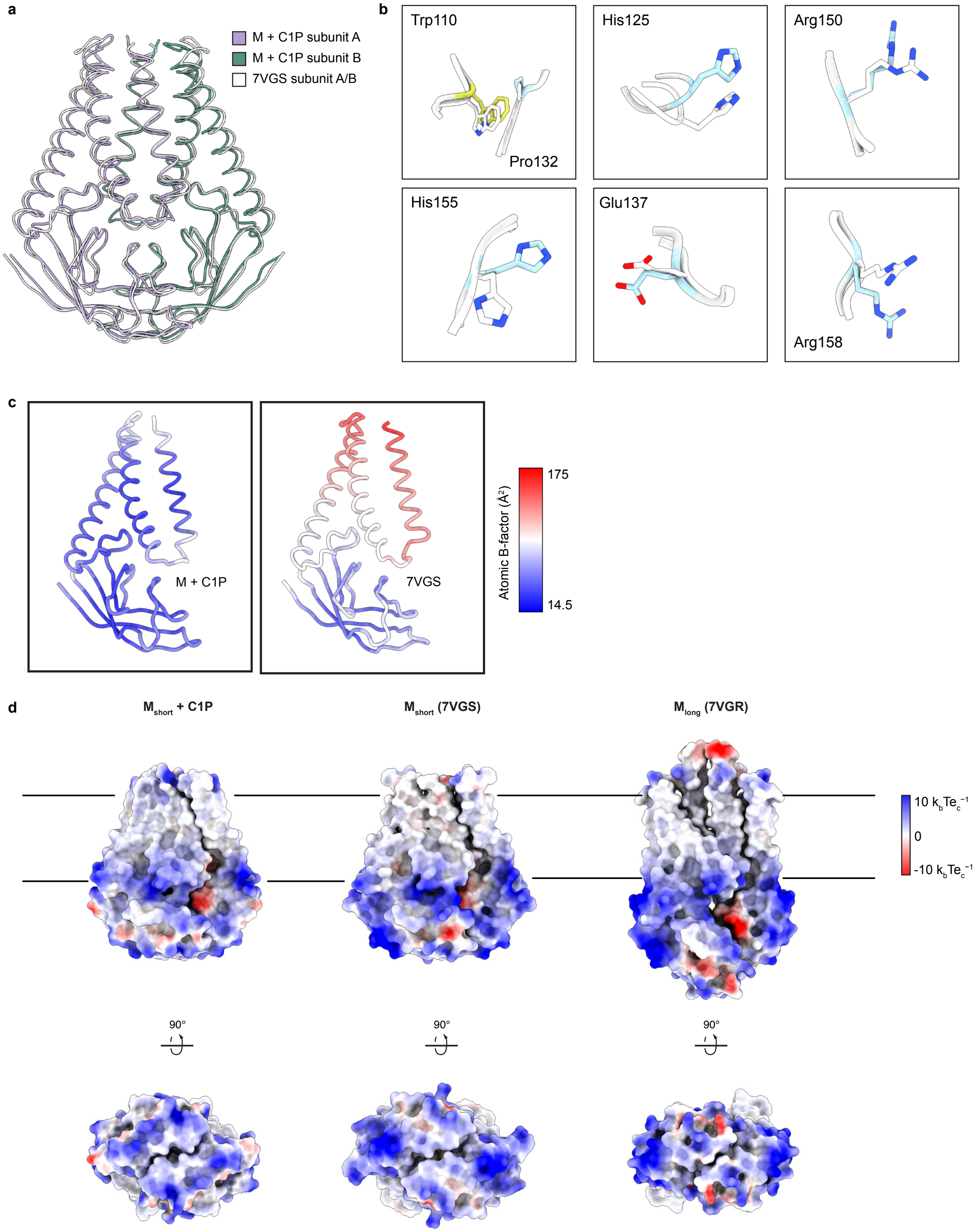

Supplementary Figure 8. C1P binding compacts and stabilizes M_{short}.
(a) Overlay of M_{short}+C1P (colored purple and green) and apo M_{short} illustrating compaction of transmembrane region. **(b)** Overlay of selected hinge and CTD residues from M_{short}+C1P (blue) and apo M_{short} structures. **(c)** Single subunit from M_{short}+C1P (left) and apo M_{short} (right) structures colored by refined B-factor. **(d)** M_{short}+C1P (left), apo M_{short}, and M_{long} electrostatic surface potential.

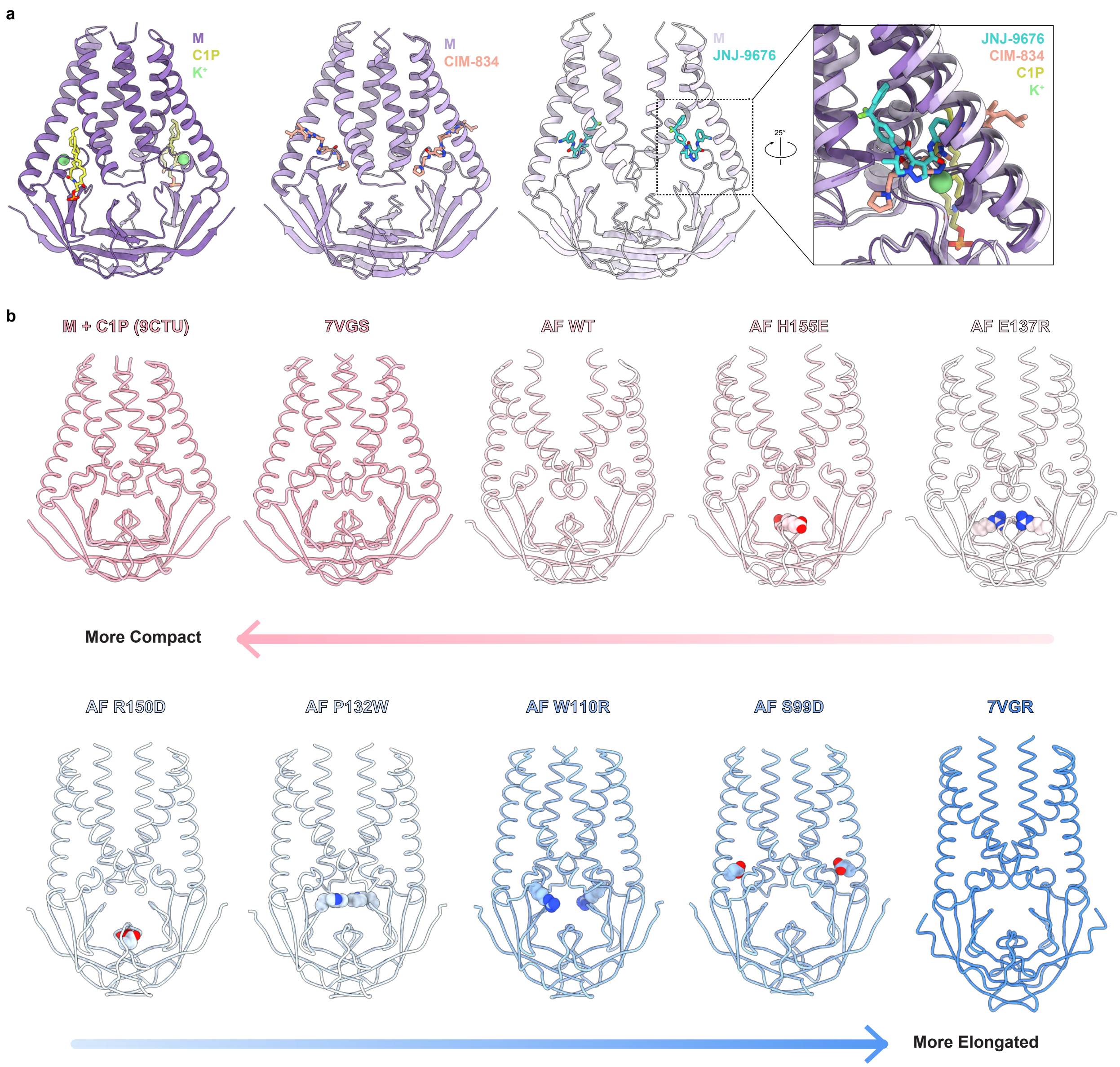

Supplementary Figure 9. Comparison of M ligand binding sites and mutations predicted to shift conformational landscape.
(a) Comparison of ligand binding sites in M. From left to right: M_{short}:C1P (PDB: 9CTU), M_{short}:CIM-834 (PDB:9EXA), M_{short}:JNJ-9676 (PDB:8W2E), and zoomed overlay. **(b)** Comparison of experimentally determined (M_{short}:C1P, apo M_{short}, and M_{long}) and Aplhafold3-predicted (AF wild-type M, H155E, E137R, R150D, P132W, W110R, and S99D) structures. Mutated sites are drawn as spheres. Mutations were hypothesized to destabilize M_{short} based on M_{short}-C1P and M_{short}-JNJ-9676 structures. Models are arranged and colored from pink (most compact) to white (intermediate between short and long conformations) to blue (most elongated).

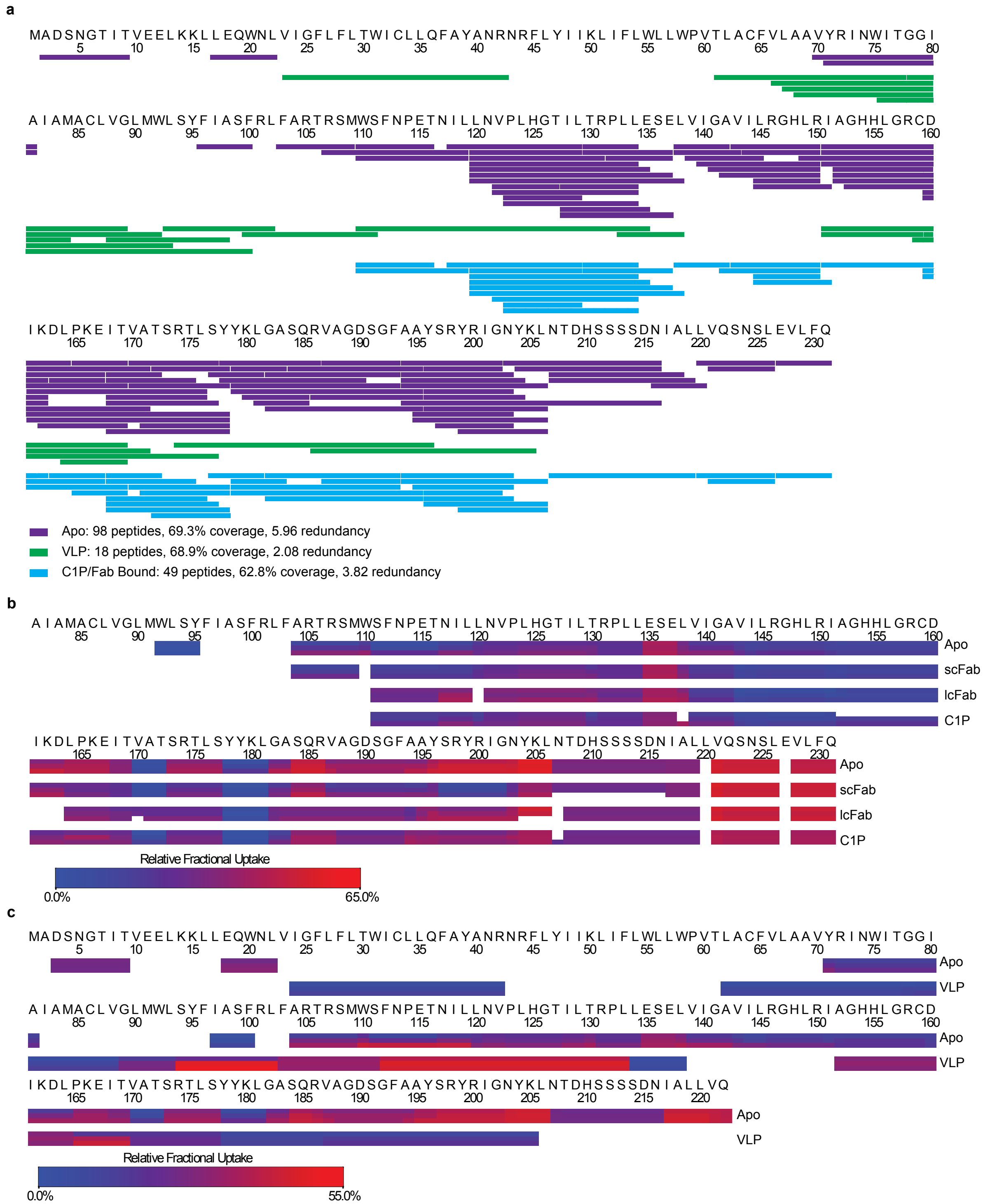

Supplementary Figure 10. Peptide coverage and deuterium exchange heatmaps.
(a) Sequence coverage by pepsin/nepenthesin peptide fragments from apo (purple), VLP-embedded (green), and C1P/- or Fab-bound (cyan) M protein. **(b)** Deuterium exchange heatmap (Dex = 1, 10, 100 min) comparing apo, scFab-bound, lcFab-bound, and C1P-bound M (top to bottom). **(c)** Deuterium exchange heatmap (Dex = 1, 10, 100 min) comparing apo (top) and VLP-embedded (bottom) M protein.

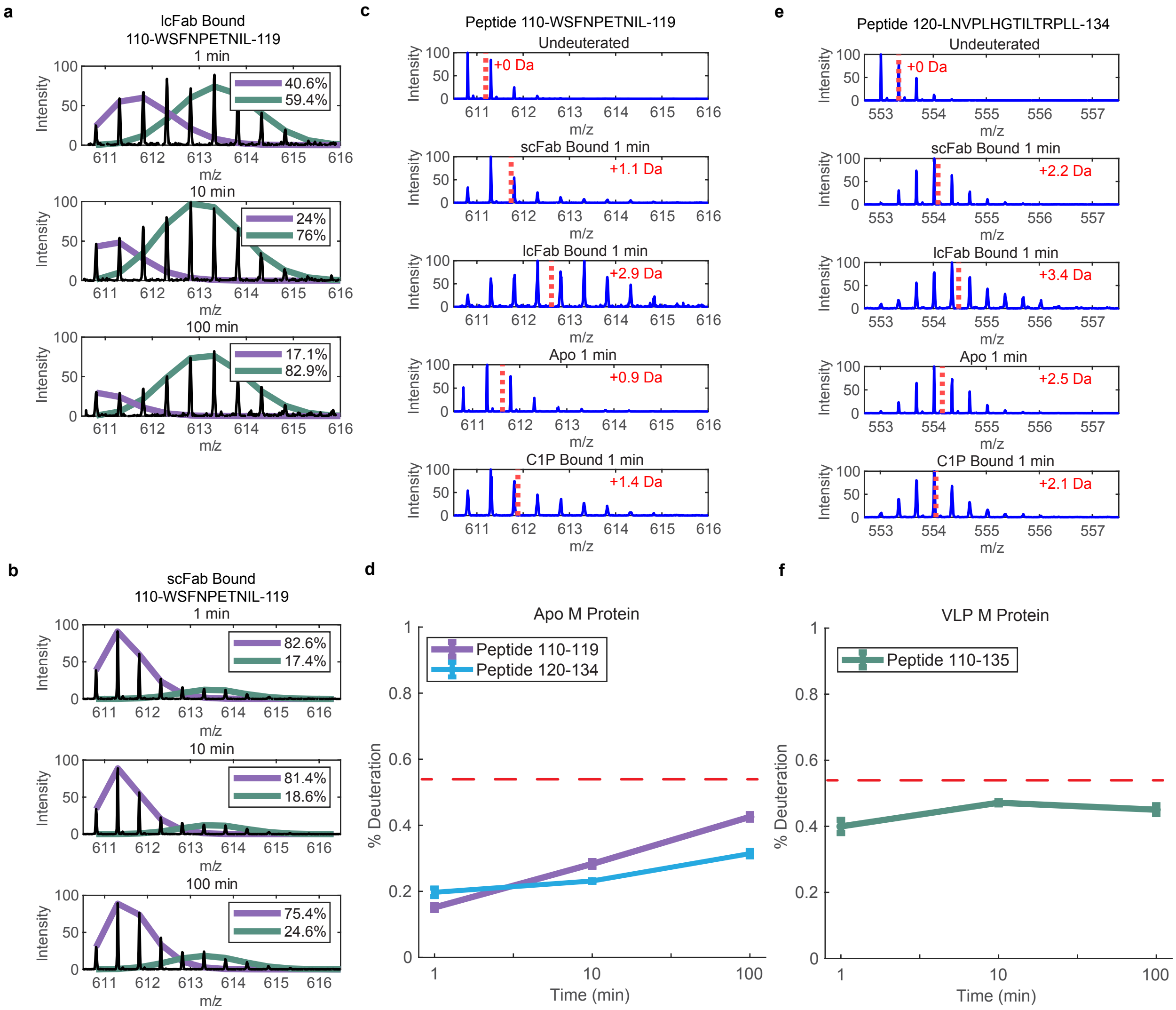

Supplementary Figure 11. Mlong sampling in apo, Fab-bound, C1P-bound, and VLP samples.
(a,b) Stacked mass spectral envelopes for hinge reporter peptide 110-119 corresponding from **(a)** scFab-bound M protein and **(b)** lcFab-bound M samples at 1 min, 10 min, and 100 min exchange (top to bottom). Bimodal spectra were deconvolved and high and low exchanging population fits are shown in green and purple, respectively. **(c)** Stacked mass spectral envelopes for hinge peptide 110-119 from an undeuterated reference, scFab-bound, lcFab-bound, apo, and C1P-bound M sample (top to bottom) after 1 min exchange. **(d)** Deuterium uptake plot showing peptide 110-119 (purple) and 120-134 (cyan) from apo M. **(e)** Stacked mass spectral envelopes for peptide 120-134 from an undeuterated reference, scFab-bound, lcFab-bound, apo, and C1P-bound M sample (top to bottom) after 1 min exchange. **(f)** Deuterium uptake plot for peptide 110-135 (green) from M in VLP samples. Maximum uptake (55%) is denoted by a red dashed line.

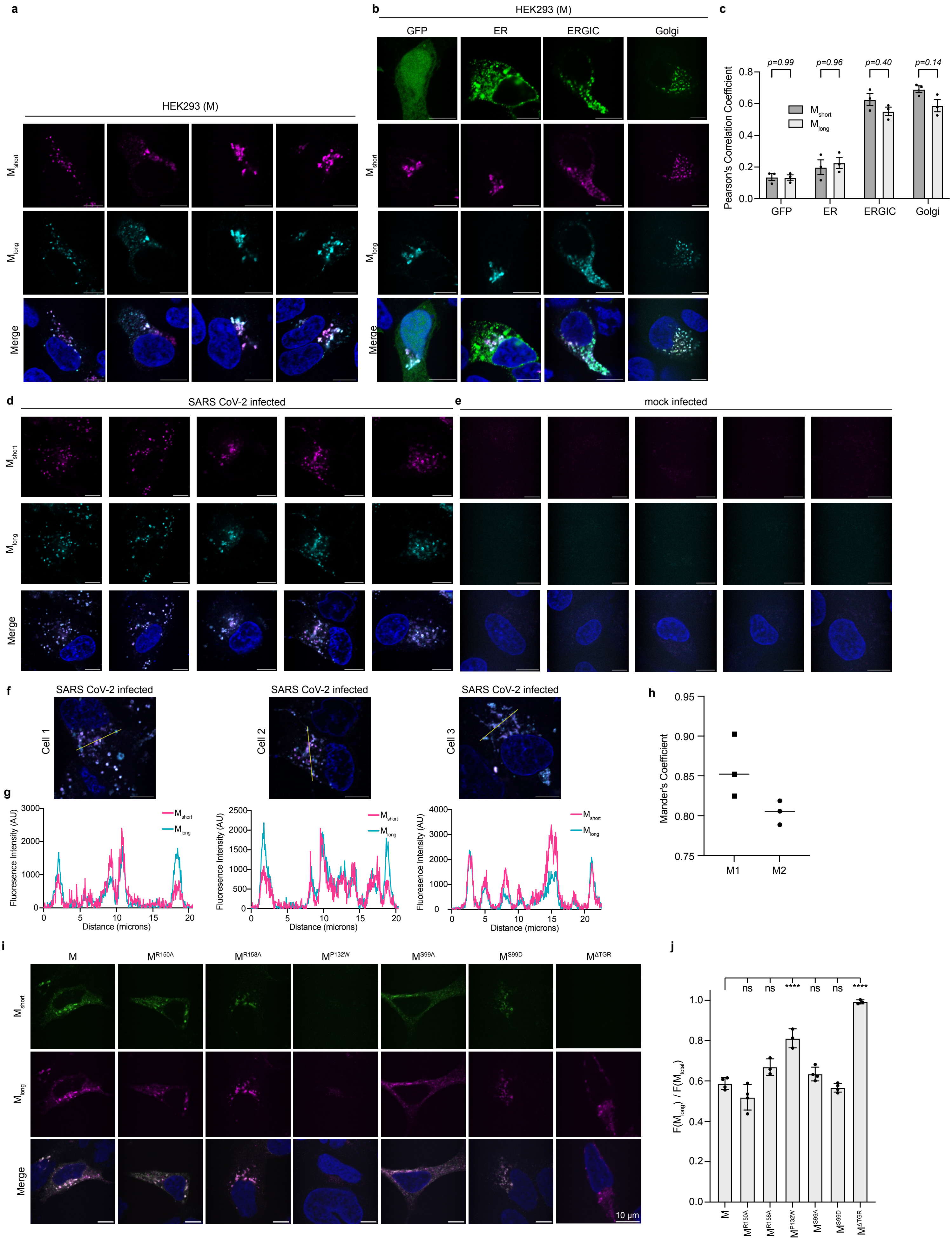
a
HEK293 (M)
Mshort
Mlong
Merge
b
HEK293 (M)
GFP
ER
ERGIC
Golgi
c
Pearson's Correlation Coefficient
p=0.99
p=0.96
p=0.40
p=0.14
Mshort
Mlong
GFP
ER
ERGIC
Golgi
d
SARS CoV-2 infected
e
mock infected
f
SARS CoV-2 infected
Cell 1
Cell 2
Cell 3
g
Fluorescence Intensity (AU)
Distance (microns)
h
Mander's Coefficient
M1
M2
i
M
MR150A
MR158A
MP132W
MS99A
MS99D
MΔTGR
10 µm
j
ns
****
F(Mlong) / F(Mtotal)

Supplementary Figure 12. Localization of M_{short} and M_{long} in cells.
(a) M_{short} and M_{long} immunolocalization in HEK293 cells transfected with M. One panel is duplicated from Figure 5a for comparison across panels. Scale bar = 10 μm. **(b)** Co-localization of M_{short} and M_{long} with markers for ER (ER-3), ERGIC (ERGIC-53), or Golgi (Golgi7) in cells transfected with M. Scale bar = 10 μm. **(c)** Quantification of colocalization experiments in **(b)**. Data are mean ± s.e.m. with differences assessed by one-way Anova with Sidak correction. **(d,e)** M_{short} and M_{long} immunolocalization in (d) SARS-CoV-2 infected (one panel is duplicated from Figure 5a for comparison across panels) and **(e)** mock infected A549-hACE2 cells. Scale bar = 10 μm. (f) M_{short} and M_{long} immunolocalization in SARS-CoV-2 infected A549-hACE2 cells. Scale bar = 10 μm. **(g)** Intensity profiles of M_{short} and M_{long} across yellow lines in (f). (h) Fraction of M_{short} that overlaps with M_{long} (M1) and fraction of M_{long} that overlaps with M_{short} (M2). **(i)** Representative HEK293 cells transfected with indicated M constructs and immunostained for M_{short} and M_{long}. (j) Quantification of M_{long} proportion (M_{long} fluorescence units/(total M (M_{long}+M_{short}) fluorescence units).

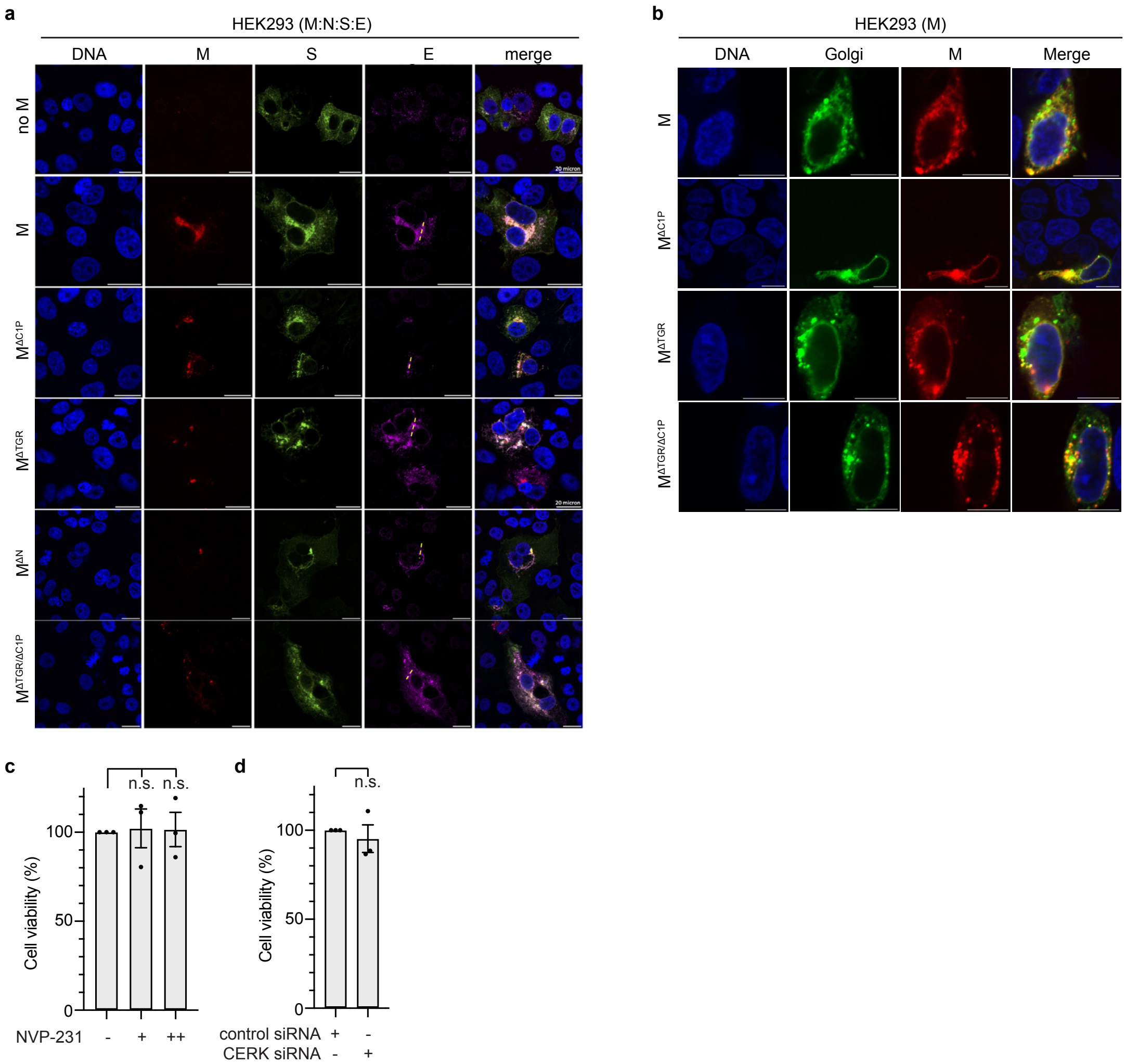

Supplementary Figure 13. M construct immunolocalization and assesment of cell viability upon CERK inhibition or knockdown.

(a) HEK293 cells transfected with S, E, N, and M constructs used in this study and immunostained for M, S, and E. Scale bar = 20 µm. **(b)** HEK293 cells transfected with mCherry-tagged M constructs and a Golgi marker (mEmerald-Golgi7). Scale bar =10 µm. **(c)** Cell viability following NVP-231 treatment assessed by MTT assay at 48 h post-treatment. Cells treated with 500 nM NVP-231, administered either once at 24 hours (+) or twice at 24 and 43 hours (++), compared to untreated cells receiving vehicle (-). Data are mean ± sem from three biological replicates (n = 3) and differences were assessed using one-way ANOVA followed by Dunnett's multiple comparisons test. No statistically significant differences were observed (p=0.9758 and 0.9874) **(d)** Cell viability following CERK knockdown evaluated by MTT assay at 96 h post-transfection. Viability is shown as percentage relative to non-targeting siRNA control (set to 100%). Data are mean ± sem from three biological replicates (n = 3) and differences were assessed using an unpaired t test with Welch's correction. No statistically significant difference was observed (p=0.6078).

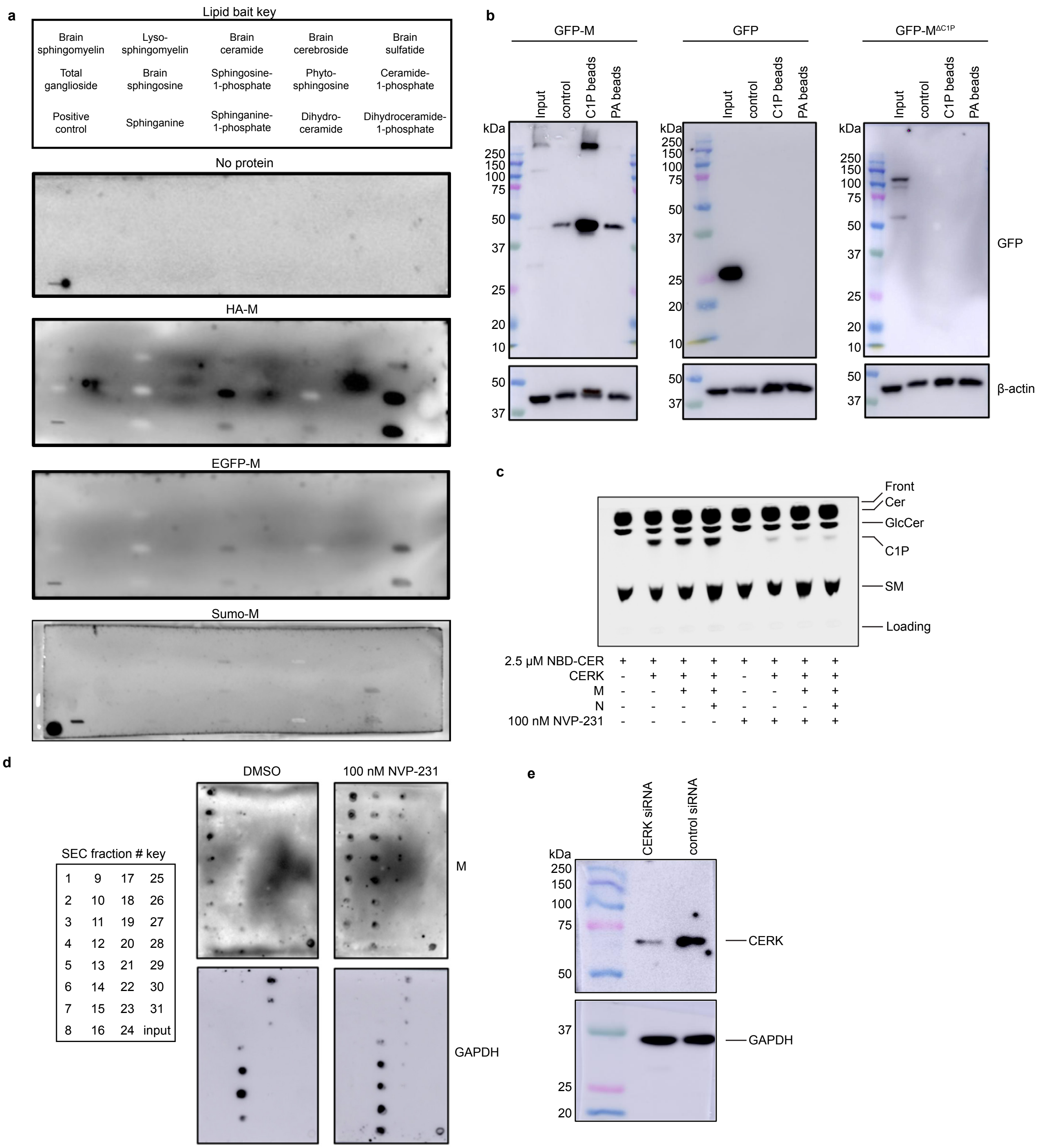

Supplementary Figure 14. Uncropped blot and thin-layer chromatography images.
(a) Sphingolipid snooper blot **(Fig. 1a)**, **(b)** lipid-coated bead pull down blots **(Figs. 1b, 5d)**, **(c)** CERK inhibition by NVP-231 assessed by TLC **(Fig. 1e)**, **(d)** blot of size chromatography fractionated lysates from DMSO- or NVP-231- treated cells expressing M, N, E, and S **(Fig. 1f)**, and **(e)** blot of cell lysates after treatment with CERK-targeting or control siRNA **(Fig. 6g).**

Supplementary Table 1 - Cryo-EM data collection, refinement, and validation statistics

	**M:scFab + C1P**	**M:lcFab + C1P**
PDB ID	9CTU	9CTW
EMDB ID	45919	45921
**Data collection**		
Total movies	6,741	8,432
Magnification	105,000x	
Voltage (kV)	300	
Electron exposure ($e^-/Å^2$)	50	
Defocus range (um)	-0.5 to -1.8	
Pixel size ($Å^2$)	0.4055 (super res)	0.848
**Processing**		
Initial particle images (no.)	351,588	561,632
Final particle images (no.)	44,736	190,251
Map resolution Masked (Å, FSC = 0.143)	3.03	3.01
Symmetry imposed	C2	C2
**Refinement**		
Model resolution (Å, FSC = 0.143 / 0.5)	3.0/3.4	2.9/3.1
Map-sharpening B factor ($Å^2$)	-108.4	-119.0
Composition		
Number of atoms	9714	9754
Number of protein residues	1240	1256
Number of ligands	4	0
RMS deviations		
Bond lengths (Å)	0.003	0.003
Bond angles (Å)	0.532	0.510
Validation		
MolProbity score	1.63	1.25
Clashscore	9.58	2.07
Ramachandran plot		
Favored (%)	97.39	96.06
Allowed (%)	2.61	3.78
Disallowed (%)	0	0.16
Rotamer outliers (%)	1.03	0.74
Mean B factor ($Å^2$)		
Protein	95.42	55.62
Ligand	73.27	-

Supplementary Table 2. HDXMS experimental summary

Dataset	Apo M	M:C1P	M:scFab	M:lcFab	Apo M (VLP conditions)	VLP embedded M
Labelling Buffer	20 mM HEPES, 150 mM KCl, 1 mM EDTA, pH 8.0<br>10x stock diluted into D_2O				20 mM Tris, 100 mM NaCl, 1 mM EDTA, pH 7.4<br>20x stock diluted into D_2O	
Quench	1.5 M guanidine hydrochloride, 0.25 M TCEP, 2.4 mM DDM				4 M guanidine hydrochloride, 0.4 M TCEP, 2.4 mM DDM	
Labelling Reaction Conditions	57 µL of labelling buffer was added to 3 µL of soluble M. After labelling, 60 µL of quench buffer was added to reduce the final pH to 2.5 at 0°C.				40 µL of labelling buffer was added to 20 µL of sample (either concentrated VLP solution or diluted soluble M). After labelling, 20 µL of quench buffer was added to reduce the final pH to 2.5 at 0°C.	
HDXMS time course (min)	1, 10, 100					
Number of peptides	98	49	49	49	98	18
Sequence coverage	69.3%	62.8%	62.8%	62.8%	69.3%	68.9%
Peptide redundancy	5.96	3.82	3.82	3.82	5.96	2.08
Reproducibility	Standard Deviation of 0.75% across technical replicates	Standard Deviation of 1.20% across technical replicates	Standard Deviation of 0.65% across technical replicates	Standard Deviation of 1.49% across technical replicates	Standard Deviation of 0.81% across technical replicates	Standard Deviation of 0.63% across technical replicates
Replicates	Three technical replicates					
