# Supplementary material for: Direct lipid interactions control SARS-CoV-2 M protein conformational dynamics and virus assembly
Source: Nat Commun. 2026 Jul 23;17:8968. doi: 10.1038/s41467-026-75229-9 (PMC13503762; doi:10.1038/s41467-026-75229-9)
Supplement: Supplementary file 2 [file 41467_2026_75229_MOESM2_ESM.pdf]

Description of additional supplementary files

Title: Supplementary Movie 1. Trajectory of simulation initiated with M_{short} without C1P.
Description: Example trajectory movie with M subunits in shades of purple and residues R44, W110, and R131 shown as sticks.

Title: Supplementary Movie 2. Trajectory of simulation initiated with M_{long} without C1P.
Description: Example trajectory movie with M subunits in shades of green and residues R44, W110, and R131 shown as sticks.

Title: Supplementary Movie 3. Trajectory of simulation initiated with M_{short} with C1P.
Description: Example trajectory movie with M subunits in shades of purple, residues R44, W110, and R131 shown as sticks, and C1P molecules near M yellow.

Title: Supplementary Movie 4. Trajectory of simulation initiated with M_{long} with C1P.
Description: Example trajectory movie with M subunits in shades of green, residues R44, W110, and R131 shown as sticks, and C1P molecules near M yellow.
