# Supplementary material for: Direct lipid interactions control SARS-CoV-2 M protein conformational dynamics and virus assembly
Source: Nat Commun. 2026 Jul 23;17:8968. doi: 10.1038/s41467-026-75229-9 (PMC13503762; doi:10.1038/s41467-026-75229-9)
Supplement: Supplementary file 3 [file 41467_2026_75229_MOESM7_ESM.pdf]

nature portfolio

Corresponding author(s): Steve Brohawn, Robert Stahelin, Gregory Voth

Last updated by author(s): Jun 10, 2026

Reporting Summary

Nature Portfolio wishes to improve the reproducibility of the work that we publish. This form provides structure for consistency and transparency in reporting. For further information on Nature Portfolio policies, see our Editorial Policies and the Editorial Policy Checklist.

Statistics

For all statistical analyses, confirm that the following items are present in the figure legend, table legend, main text, or Methods section.

n/a	Confirmed	
☐	☒	The exact sample size (*n*) for each experimental group/condition, given as a discrete number and unit of measurement
☐	☒	A statement on whether measurements were taken from distinct samples or whether the same sample was measured repeatedly
☐	☒	The statistical test(s) used AND whether they are one- or two-sided<br>*Only common tests should be described solely by name; describe more complex techniques in the Methods section.*
☒	☐	A description of all covariates tested
☐	☒	A description of any assumptions or corrections, such as tests of normality and adjustment for multiple comparisons
☐	☒	A full description of the statistical parameters including central tendency (e.g. means) or other basic estimates (e.g. regression coefficient) AND variation (e.g. standard deviation) or associated estimates of uncertainty (e.g. confidence intervals)
☐	☒	For null hypothesis testing, the test statistic (e.g. F, t, r) with confidence intervals, effect sizes, degrees of freedom and P value noted<br>*Give P values as exact values whenever suitable.*
☒	☐	For Bayesian analysis, information on the choice of priors and Markov chain Monte Carlo settings
☒	☐	For hierarchical and complex designs, identification of the appropriate level for tests and full reporting of outcomes
☐	☒	Estimates of effect sizes (e.g. Cohen's *d*, Pearson's *r*), indicating how they were calculated

Our web collection on statistics for biologists contains articles on many of the points above.

Software and code

Policy information about availability of computer code

Data collection	Cryo-EM data were collected with SerialEM v4.1. Molecular dynamics was performed with GROMACS v2019 with CHARMM36 force field. Confocal microscopy images were collected with NIS-Elements.
Data analysis	Cryo-EM data were processed with cryoSPARC v4.4.1. Alphafold3 was used to predict protein structures. Structures were built in Coot v0.9.8.8.91 and refined with Phenix v1.21-5207. Structures were analyzed with Metric Ion classification v1 and Check My Metal v2.1. Molecular dynamics analysis used MDAnalysis v2.1.0, PyLipID v1.5.14, MEMBPLUGIN v1.1, VMD v1.9.4, and PyMOL v1.20. Confocal microscopy images were analyzed with ImageJ v1.53d and 1.54c. Figures were generated with Illustrator v29, ChimeraX v1.6.1, and Graphpad Prism v10.3.0. Peptides were identified from mass spectra using Protein Lynx Global Server v3.0, deuterium exchange was quantified uusing DynamX v3.0, and bimodal mass spectral envelope deconvolution was completed using HX-express3 v39.

For manuscripts utilizing custom algorithms or software that are central to the research but not yet described in published literature, software must be made available to editors and reviewers. We strongly encourage code deposition in a community repository (e.g. GitHub). See the Nature Portfolio guidelines for submitting code & software for further information.

Data

Policy information about availability of data

All manuscripts must include a data availability statement. This statement should provide the following information, where applicable:

- Accession codes, unique identifiers, or web links for publicly available datasets
- A description of any restrictions on data availability
- For clinical datasets or third party data, please ensure that the statement adheres to our policy

The final cryo-EM maps and atomic models of M:scFab + C1P and M:lcFab + C1P generated in this study have been deposited in the Electron Microscopy Data Bank under accession codes EMD-45919 [https://www.ebi.ac.uk/emdb/EMD-45919] and EMD- 45921 [https://www.ebi.ac.uk/emdb/EMD-45921] and in the PDB under accession codes 9CTU [https://doi.org/10.2210/pdb9ctu/pdb] and 9CTW [https://doi.org/10.2210/pdb9ct/pdb], respectively. HDXMS data have been deposited in the ProteomeXchange Consortium via the PRIDE partner repository with accession number PXD079401 [https://www.ebi.ac.uk/pride/archive/projects/PXD079401]. Source data are provided as a Source Data file.
The initial structures, GROMACS input files for the atomistic simulations, and PLUMED scripts used in this study are available at https://zenodo.org/records/20647068. The analysis scripts used for data processing are available at: https://github.com/mandirad/Direct-lipid-interactions-control-SARS-CoV-2-M-protein-conformational-dynamics-and-virus-assembly.

Research involving human participants, their data, or biological material

Policy information about studies with human participants or human data. See also policy information about sex, gender (identity/presentation), and sexual orientation and race, ethnicity and racism.

Reporting on sex and gender	Not applicable to this study.
Reporting on race, ethnicity, or other socially relevant groupings	Not applicable to this study.
Population characteristics	Not applicable to this study.
Recruitment	Not applicable to this study.
Ethics oversight	Not applicable to this study.

Note that full information on the approval of the study protocol must also be provided in the manuscript.

Field-specific reporting

Please select the one below that is the best fit for your research. If you are not sure, read the appropriate sections before making your selection.

☒ Life sciences ☐ Behavioural & social sciences ☐ Ecological, evolutionary & environmental sciences

For a reference copy of the document with all sections, see nature.com/documents/nr-reporting-summary-flat.pdf

Life sciences study design

All studies must disclose on these points even when the disclosure is negative.

Sample size	No sample size calculation was performed. Sample sizes were selected based on norms in the field.
Data exclusions	No data were excluded from analysis.
Replication	All attempts are replication were successful.
Randomization	There was no feasible way to randomize data in this study.
Blinding	There was no feasible way to blind experimenters to data in this study.

Reporting for specific materials, systems and methods

We require information from authors about some types of materials, experimental systems and methods used in many studies. Here, indicate whether each material, system or method listed is relevant to your study. If you are not sure if a list item applies to your research, read the appropriate section before selecting a response.

Materials & experimental systems

n/a	Involved in the study
☐	☒ Antibodies
☐	☒ Eukaryotic cell lines
☒	☐ Palaeontology and archaeology
☒	☐ Animals and other organisms
☒	☐ Clinical data
☒	☐ Dual use research of concern
☒	☐ Plants

Methods

n/a	Involved in the study
☒	☐ ChIP-seq
☒	☐ Flow cytometry
☒	☐ MRI-based neuroimaging

Antibodies

Antibodies used	Abcam ab49763 Anti-DDDDK tag antibody [M2], ThermoFisher Scientific AM4300 GAPDH Monoclonal antibody (6C5), Abcam ab1190 Anti-HA tag antibody (HRP) (ab1190), Rockland 100-401-A55 SARS-CoV Membrane Protein Antibody. Anti-Mshort and anti-Mlong Fabs were expressed, purified, labeled, and used as described in the Methods.
Validation	Antibodies were validated by the manufacturer or in control experiments presented in the manuscript.

Eukaryotic cell lines

Policy information about cell lines and Sex and Gender in Research

Cell line source(s)	HEK293 ATCC CRL-1573, Vero E6 ATCC CRL-1586, COS-7 ATCC CRL-1651
Authentication	Cell lines were authenticated by the manufacturer and not further authenticated.
Mycoplasma contamination	Mycoplasma detection done with ThermoFisher M7006 Molecular Probes MycoFluor Mycoplasma detection kit
Commonly misidentified lines (See ICLAC register)	*Name any commonly misidentified cell lines used in the study and provide a rationale for their use.*

Plants

Seed stocks	*Report on the source of all seed stocks or other plant material used. If applicable, state the seed stock centre and catalogue number. If plant specimens were collected from the field, describe the collection location, date and sampling procedures.*
Novel plant genotypes	*Describe the methods by which all novel plant genotypes were produced. This includes those generated by transgenic approaches, gene editing, chemical/radiation-based mutagenesis and hybridization. For transgenic lines, describe the transformation method, the number of independent lines analyzed and the generation upon which experiments were performed. For gene-edited lines, describe the editor used, the endogenous sequence targeted for editing, the targeting guide RNA sequence (if applicable) and how the editor was applied.*
Authentication	*Describe any authentication procedures for each seed stock used or novel genotype generated. Describe any experiments used to assess the effect of a mutation and, where applicable, how potential secondary effects (e.g. second site T-DNA insertions, mosiacism, off-target gene editing) were examined.*
