# Supplementary material for: Direct lipid interactions control SARS-CoV-2 M protein conformational dynamics and virus assembly
Source: Nat Commun. 2026 Jul 23;17:8968. doi: 10.1038/s41467-026-75229-9 (PMC13503762; doi:10.1038/s41467-026-75229-9)
Supplement: Supplementary file 4 [file 41467_2026_75229_MOESM8_ESM.pdf]

nature portfolio

Peer Review File

Direct lipid interactions control SARS-CoV-2 M protein conformational dynamics and virus assembly

Corresponding Author: Professor Stephen Brohawn

This manuscript has been previously reviewed at another journal that is not operating a transparent peer review scheme. The manuscript was considered suitable for publication without further review at Nature Communications.

This file contains all reviewer reports in order by version, followed by all author rebuttals in order by version.

Open Access This Peer Review File is licensed under a Creative Commons Attribution 4.0 International License, which permits use, sharing, adaptation, distribution and reproduction in any medium or format, as long as you give appropriate credit to the original author(s) and the source, provide a link to the Creative Commons license, and indicate if changes were made.
In cases where reviewers are anonymous, credit should be given to 'Anonymous Referee' and the source.
The images or other third party material in this Peer Review File are included in the article's Creative Commons license, unless indicated otherwise in a credit line to the material. If material is not included in the article's Creative Commons license and your intended use is not permitted by statutory regulation or exceeds the permitted use, you will need to obtain permission directly from the copyright holder.
To view a copy of this license, visit https://creativecommons.org/licenses/by/4.0/

We thank the reviewers for providing critical analysis of the most recent version of our manuscript.

Referee #1 (Remarks to the Author):

The authors have provided additional M mutants demonstrating that both antibodies can bind both of these variants, with the exception of MΔTGR (Golgi retention signal for MERS M). However, antibody binding to Mshort and Mlong has still not been validated using CERK siRNA or treatment with NVP-231, as previously suggested. Such experiments would provide a more direct validation of the antibody specificity.

Response: We would like to emphasize that antibody specificity is already rigorously supported by multiple independent lines of evidence in our study, including (i) structure-guided epitope definition using previously published cryo-EM structures of conformation-specific Fabs (PMID: 35931673), (ii) mutation of residues within the structurally defined Mshort antibody epitope (MΔTGR), which selectively abolishes Mshort antibody labeling while preserving Mlong detection, as expected, and (iii) additional conformational mutants (e.g., P132S, C1P-binding mutants) that shift the equilibrium toward Mlong and are correspondingly detected by the Mlong antibody. These results provide orthogonal and mechanistically grounded validation of antibody specificity. Importantly, these reagents and their conformational selectivity have also been independently validated in the literature by others, including in two papers published in Nature last year (PMIDs: 35931673, 40140563, 40140569), all clearly referenced in our manuscript. Specificity is expected and obvious from structures reported by four separate groups - each antibody recognizes an epitope that is exposed in only one conformation. The requested CERK perturbation experiments would not directly test antibody specificity, but rather alter the biological system under study, and therefore do not constitute a more direct validation than the structural and mutational approaches already employed.

The authors have also included new data using a VLP luciferase release assay, which improves the manuscript.

Response: Thank you for noting this important experiment.

It remains unclear how the intracellular antibody labeling supports the model proposed in Fig. 6i. Importantly, the presented fluorescence microscopy data do not have sufficient spatial resolution to distinguish virions that have already been released into the ERGIC lumen from budding particles at the ERGIC membrane. Therefore, the current data may not be sufficient to conclusively support the authors interpretation. Additional antibody validation using purified released SARS-CoV-2 virions and VLPs analyzed by confocal microscopy would help clarify this point. This experiment using the specific antibodies is particularly important because the central claim of the study is that extracellular virions contain predominantly Mlong but not Mshort, whereas budding virions contain higher levels of Mshort. This interpretation would also need to be reconciled with previous cryo-EM studies of murine hepatitis virus (MHV), which reported that both Mlong and Mshort populations are present within virions (Neuman BW, JSB, 2010).

Response: The reviewer’s concern regarding interpretation of intracellular immunofluorescence data and the distinction between budding versus released virions appears to apply a standard that exceeds the resolution limits of conventional fluorescence microscopy. We agree that fluorescence imaging cannot definitively distinguish luminal virions from budding intermediates; however, our conclusions do not rely on this distinction alone. Rather, they are supported by a convergence of independent approaches, including HDX-MS, cryo-EM structures, molecular dynamics simulations, and functional VLP assays, all of which consistently support distinct roles for Mshort and Mlong conformations. We have also tempered the language in the revised manuscript to reflect these limitations.

Third, the request for additional validation using purified virions or VLPs analyzed by confocal microscopy represents a substantial expansion of scope beyond the original and revised reviewer requests. Notably, the central findings regarding conformational distribution are already supported by HDX-MS measurements of M embedded in VLPs, which directly demonstrate enrichment of Mlong-like conformations in assembled particles. These data provide a more direct and quantitative assessment than fluorescence-based approaches.

It is also not clear how the authors validated that MΔTGR assumes only the Mlong conformation, as now suggested based on the immunofluorescence data. Were additional approaches such as cryo-EM or HDX-MS

performed to support this conclusion? Without further validation, it is difficult to interpret this new result as evidence supporting the proposed model.

Response: The ΔTGR mutation includes M residues in the structurally defined binding epitope for the M_{short} antibody, but not part of the M_{long} antibody binding epitope, as stated in the manuscript. The ΔTGR mutation was not used to assess if the ΔTGR mutation favored M_{long} or M_{short} per se, but was used to validate the structurally defined binding mode of the M_{short} antibody.

The authors state that M does not colocalize with the ER; however, no data supporting this claim are shown. In contrast, Supplementary Fig. 3c includes a Golgi marker rather than an ER marker.

Response: This is an important point and we are sorry for the lack of clarity in our original writing. The text and figure legend text were correct, but we mislabeled the last version of Figure S3c. It said Golgi, but should have been ER, showing lack of colocalization. We have now corrected the label in Figure S3c. Further, ER labeling in the presence of M was performed in the last version of the manuscript, but not clearly referenced in the text. We have now revised the text to specifically cite Figures 5b-c and S12b-c, which show little ER localization of M compared to a GFP control (in contrast to M colocalization with the Golgi and ERGIC markers).

The analysis presented in Fig. S3F is unclear. It is not evident how particle diameter can be derived from the presented 2D contours. The authors should consider performing 3D segmentation or, at minimum, reporting particle area rather than diameter.

Response: After the 3rd round of review, we understand the concern but left as apparent diameter for the following reason. We originally did vesicle diameter to compare to the vesicle area of SARS-CoV-2 infection (reference 22). Since this reference did not measure vesicle area, we left our original diameter assessment in as it directly compared with the authentic SARS-CoV-2 infection for vesicle diameter (S, E, M and N expression compared to SARS-CoV-2 infection). This VLP model aslo fits with previous work by the Stahelin (Plescia et al. 2021 J. Biol. Chem.) and other laboratories (Reviewed in Sultana and Stahelin 2025 Virology).

It is also unclear how the fraction of Mlong shown in Fig. 5H was determined. A more detailed description of the analysis would be helpful. In the Methods section, it is not specified whether individual cells were segmented or how exactly the ratio was calculated and normalized. The current description states only that “the integrated fluorescence intensity for the Mshort and Mlong channels was measured in Fiji/ImageJ,” which is insufficient to reproduce the analysis.

Response: Thanks for noting this. We have revised the methods section to include more specific details on the method for determining the fraction of Mlong.

Finally, the statement that "MΔTGR includes mutations in the Mshort but not the Mlong antibody epitope and is therefore expected to specifically disrupt Mshort labeling" requires clarification. Both conformations should contain the same amino acid sequence. The authors should provide precise information about the epitopes recognized by the Mlong and Mshort antibodies.

Response: We believe the reviewer misunderstands the statement, our description of this mutation, and/or the nature of the antibody-M interactions. These epitopes have been structurally characterized in cryo-EM structures of M-Fab complexes by four groups, including ours in this paper. The others are cited in the manuscript (PMIDs: 35931673, 40140563, 40140569). The Mshort and Mlong antibodies bind three-dimensional epitopes that comprise different M amino acids. The MΔTGR mutation includes residues that interact with the M_{short} antibody, but not the M_{long} antibody. The ΔTGR mutation was not used to assess if the ΔTGR mutation favored M_{long} or M_{short}, but was used to validate the binding of M_{short} antibody as confirmed via structural studies of the antibody.

In addition, one panel from the SARS-CoV-2 infection figure appears to be duplicated in Supplementary Fig. 12.

Response: Thank you for pointing this out. We have added a note to both figure legends (Fig. 5a and Supplementary Figure 12) to state that one panel is duplicated for comparison’s sake across the different replicates shown in Supplementary Figure 12.

Scale bars in fluorescence microscopy images should be consistent throughout the manuscript, including the supplementary figures. Units should be written as μm. Furthermore, scale bars appear to be missing from Fig. 1.

Response: We have done our best to have the same scale bar and label on as many figures as possible. Keep in mind at times a different magnification is needed so it isn't always practical to use the same size scale bar. Further, data in this paper were collected on more than 3 different microscopes over the course of nearly six years so keeping all figure scale bars the same is not always practical. For clarity, we have updated any figure legend that clearly didn't state the scale bar size. We have put in new scale bars in Figures 1, 5, and 6.

Referee #4 (Remarks to the Author):

Overall, the changes made by the authors have addressed most of my original concerns. Namely presentation of the HDXMS data draws key points forward, and the brief but useful discussion about what factors in VLP may lead to enrichment of M-long are helpful.

The one remaining point I would suggest is that I would like to see more clarity in presentation of how each approach treats membrane organization and discussion of limitations associated with each. The study is somewhat loose in how membrane is discussed. It should be noted that how membrane is represented in MD simulations, cryo-EM and HDX-MS structures, and in VLP are all different with apparently symmetrical leaflets composed of what is described as ERGIC-like composition, detergent micelles, and virus-like particle (VLP) membranes produced by mammalian cells. Given that the central focus is on the contribution of lipid (specifically C1P) to M conformation and SARS-CoV-2 assembly, having as much overlap of these conditions as possible to understand how each finding complements the other seems like it would be important.

Response: We thank the reviewer for this insightful comment and agree that greater clarity regarding membrane representation across the different experimental and computational approaches would strengthen the manuscript. We have revised the Discussion to more explicitly describe how membrane organization is treated in each platform and to discuss the associated limitations and complementarity of these approaches.

Specifically, we now clarify that the molecular dynamics (MD) simulations employ symmetric bilayers with ERGIC-like lipid compositions designed to isolate lipid-dependent effects on M protein conformational dynamics under controlled conditions. While these bilayers do not fully capture the compositional asymmetry and lateral heterogeneity expected in native ERGIC membranes, they provide a tractable framework to examine how specific lipids, including ceramide-1-phosphate (C1P), influence M protein structure and membrane interactions.

We further note that the cryo-EM structures were determined in detergent micelles, which provide stabilization of membrane protein architecture but cannot fully recapitulate the biophysical properties of intact viral or ERGIC membranes. Similarly, HDX-MS experiments were performed using detergent-solubilized or membrane-associated protein preparations that preserve important conformational features but do not fully reproduce native membrane organization.

Finally, we clarify that the virus-like particles (VLPs) are assembled in mammalian cells and therefore likely possess substantially more complex and asymmetric membrane compositions than the simplified membranes used in simulations or biochemical assays. Although the precise lipid composition of VLP membranes is not experimentally defined here, these systems provide an important cellular context for validating the functional relevance of the observed M conformational states.

Importantly, despite these methodological differences, all approaches converged on a consistent model in which C1P stabilizes a specific M protein conformational ensemble linked to viral assembly. We agree with the reviewer that exact overlap between systems is inherently difficult to achieve across structural, computational, and cellular platforms. However, we believe the convergence of these orthogonal approaches strengthens the biological significance of the findings rather than detracts from them.

To improve clarity for readers, we have added additional discussion of these points in the revised manuscript and explicitly outline the membrane context and limitations associated with each methodology

Specific items:

It also wasn't clear to me, though I may have overlooked this, the description of whether the lipid bilayer in the MD simulations was constructed with the same composition in both leaflets or accounted for asymmetry in lipid distributions. This likely would significantly influence M behavior, and while it may be beyond the scope of the present study to compare symmetrical vs asymmetric lipid distributions, addressing that factor seems important.

Response: We thank the reviewer for raising this important point. In the present study, the lipid bilayer in the MD simulations was constructed as a symmetric membrane, with identical lipid composition in both leaflets.

We acknowledge that lipid asymmetry is a fundamental feature of biological membranes and can significantly influence membrane protein behavior. However, the leaflet-specific lipid composition of the ERGIC membrane remains incompletely characterized, particularly with respect to quantitative lipid distributions and the localization of signaling lipids such as C1P. Therefore, we employed a symmetric ERGIC-like membrane composition to provide a controlled framework for isolating lipid-dependent effects on M-protein conformation.

Figure 4f blow-up panels are highly pixelated and structures are washed out.

Response: Thanks for noting this. There appears to have been an issue in converting our original images for the reviewed pdf. This has been corrected.
